# Supplementary figures and images for: Meis1 isoform diversity orchestrates neural progenitor differentiation by regulating ATOH1 degradation at distinct subcellular compartments
Source: PLoS Biol. 2026 Jul 13;24(7):e3003897. doi: 10.1371/journal.pbio.3003897 (PMC13379096; doi:10.1371/journal.pbio.3003897)

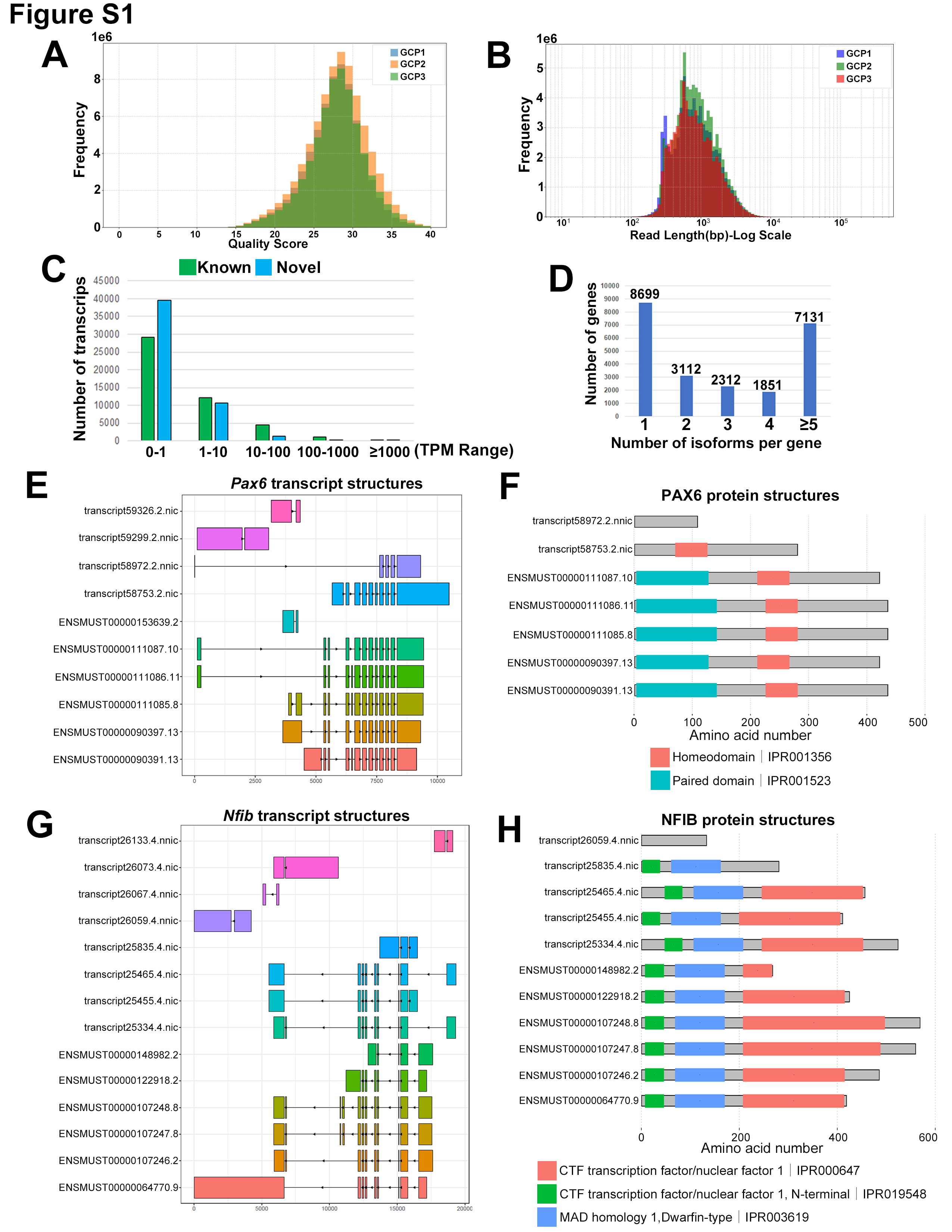

Supplement: S1 Fig — A. Histogram showing the distribution of read quality scores (Q-scores) for Nanopore long-read cDNA sequencing data from GCP samples. B. Histogram showing the distribution of read lengths for Nanopore long-read cDNA sequencing data from GCP samples. C. Histograms showing the distribution of expression levels (Transcripts Per Million, TPM) for known transcripts (left) and novel transcripts (right). D. Distribution of the number of isoforms per gene, including all identified transcripts without TPM filtering. The graph shows the percentage of genes binned by their isoform count (1, 2, 3, 4, or ≥5). E. Transcript isoform structures of Pax6, generated using ggtranscript. F. Predicted protein domain structures of coding PAX6 isoforms. Protein sequences were obtained via Sqanti3, with domains predicted by InterPro and visualized using drawProteins. Key conserved domains are shown. Some isoforms lack the paired domain but retain the homeodomain, and a short isoform lacks all canonical domains. G. Transcript isoform structures of Nfib, generated using ggtranscript. H. Predicted protein domain structures of coding Nfib isoforms. Protein sequences were obtained via Sqanti3, with domains predicted by InterPro and visualized using drawProteins. Some isoforms contain large deletions, including a short variant that lacks all predicted domains. The data underlying this figure can be found in S5 Data. (TIF) [file pbio.3003897.s001.tif]

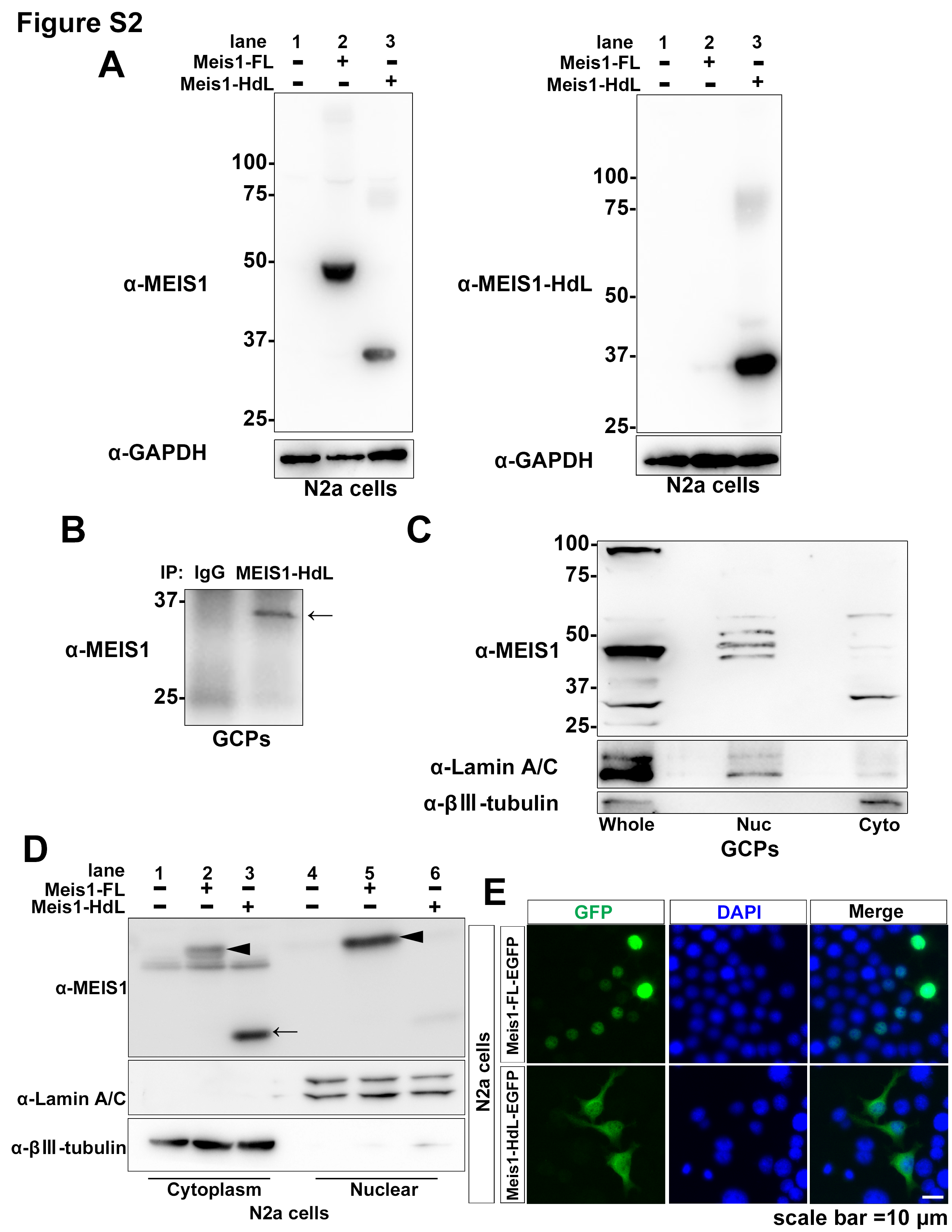

Supplement: S2 Fig — A. Validation of the MEIS1-HdL-specific antibody by immunoblot. Lysates from untransfected N2a cells or cells expressing MEIS1-FL or MEIS1-HdL were analyzed. The left panel, probed with a pan-MEIS1 antibody, detects both isoforms. The right panel shows that the newly generated antibody specifically recognizes MEIS1-HdL and not MEIS1-FL. B. Immunoprecipitation of endogenous MEIS1-HdL from P7 GCP lysates using the MEIS1-HdL-specific antibody, followed by immunoblotting with a pan-MEIS1 antibody. A single band corresponding to endogenous MEIS1-HdL was detected. C. Subcellular localization of endogenous MEIS1 isoforms in P7 GCPs, analyzed by immunoblotting of subcellular fractions. The blot shows that MEIS1-FL is predominantly nuclear, while MEIS1-HdL is primarily cytoplasmic. Lamin A/C and βIII-tubulin were used as nuclear and cytoplasmic fraction markers, respectively. D. Immunoblot analysis showing the subcellular localization of overexpressed MEIS1 isoforms in N2a cells. Following transfection with untagged MEIS1-FL or MEIS1-HdL, cytoplasmic and nuclear fractions were blotted for MEIS1. The results confirm the predominantly nuclear localization of MEIS1-FL (arrowheads) and cytoplasmic localization of MEIS1-HdL (arrows). Lamin A/C and βIII-tubulin serve as nuclear and cytoplasmic fraction markers, respectively. E. Representative immunofluorescence images showing subcellular localization of MEIS1-FL-EGFP and MEIS1-HdL-EGFP in N2a cells. Cells were transfected with MEIS1-FL-EGFP or MEIS1-HdL-EGFP expression vectors and stained for GFP (green) and DAPI (blue, for nuclei). The data underlying this figure can be found in S5 Data. (TIF) [file pbio.3003897.s002.tif]

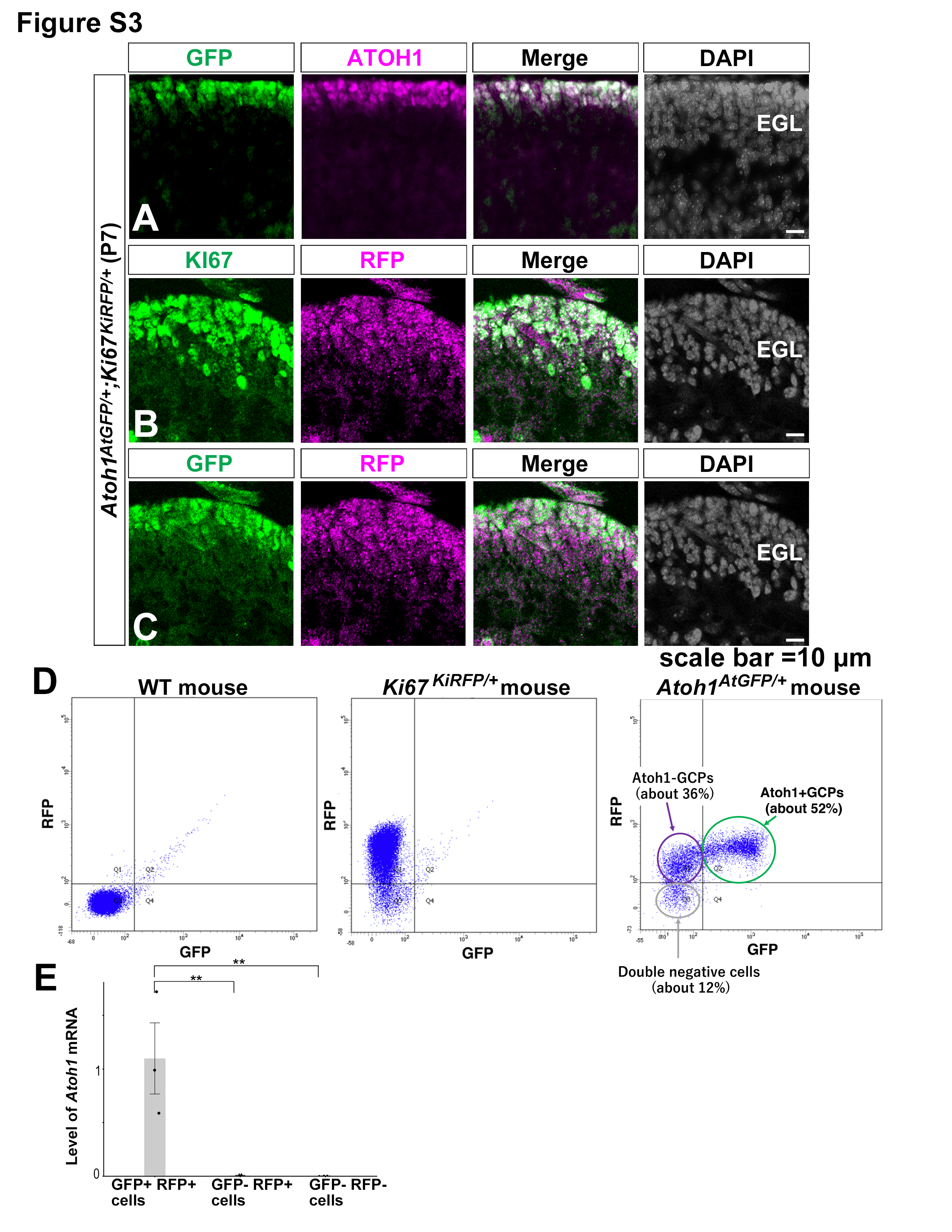

Supplement: S3 Fig — A. Validation of the Atoh1-GFP reporter in the P7 cerebellum of an Atoh1AtGFP/+; Ki67KiRFP/+ mouse. Representative images show co-localization of the GFP signal (green) with immunostaining for the endogenous ATOH1 protein (magenta). B. Validation of the Ki67-RFP reporter in the P7 cerebellum of an Atoh1AtGFP/+; Ki67KiRFP/+ mouse. Representative images show co-localization of the RFP signal (magenta) with immunostaining for the endogenous KI67 protein (green). C. Combined expression patterns of ATOH1-GFP (green) and KI67-RFP (magenta) in the P7 cerebellum. The images show that while both reporters are expressed predominantly in the outer external granular layer (oEGL), ATOH1-GFP expression is more tightly restricted to the outermost cell layer compared to the broader expression of KI67-RFP. D. Representative FACS plots demonstrating the gating strategy for isolating distinct granule cell lineage populations from purified GCPs of Atoh1AtGFP/+; Ki67KiRFP/+ mice. Wild-type (WT) GCPs are used for negative control gating. E. Relative expression levels of Atoh1 transcripts, estimated by qRT-PCR, in FACS-sorted GCP populations purified from P6 cerebella. Atoh1 expression is exclusively detected in the GFP+RFP+ fraction, confirming the specificity of this highly proliferative population. (n = 3 samples from 3 mice). The data underlying this figure can be found in S5 Data. (TIF) [file pbio.3003897.s003.tif]

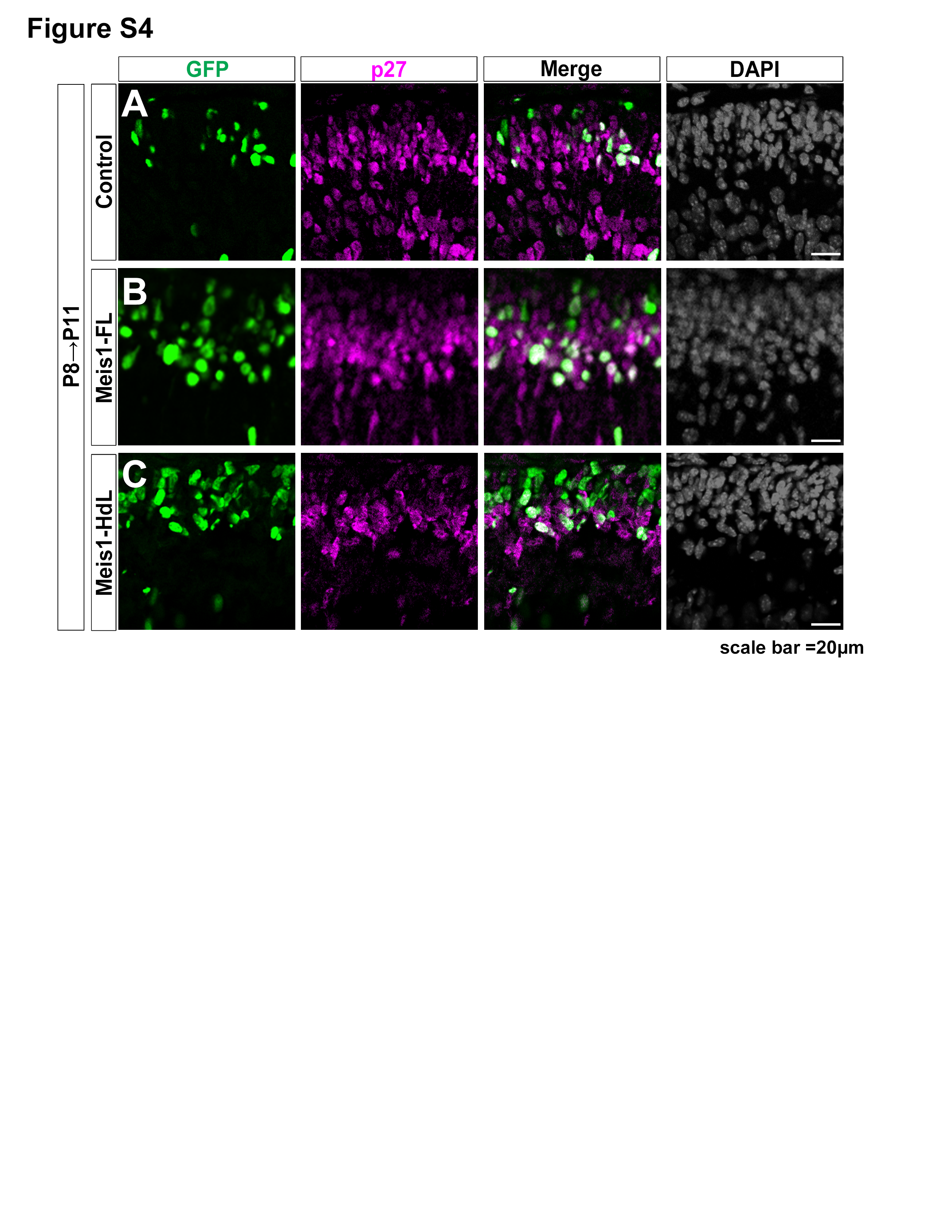

Supplement: S4 Fig — A–C. Representative immunofluorescence images of P11 cerebella following in vivo electroporation at P8. Cerebellar sections were immunostained for p27 (magenta). Electroporated cells, identified by co-electroporated H3.1-EGFP (green), were transfected with a control vector (A), MEIS1-FL (B), or MEIS1-HdL (C). The data underlying this figure can be found in S5 Data. (TIF) [file pbio.3003897.s004.tif]

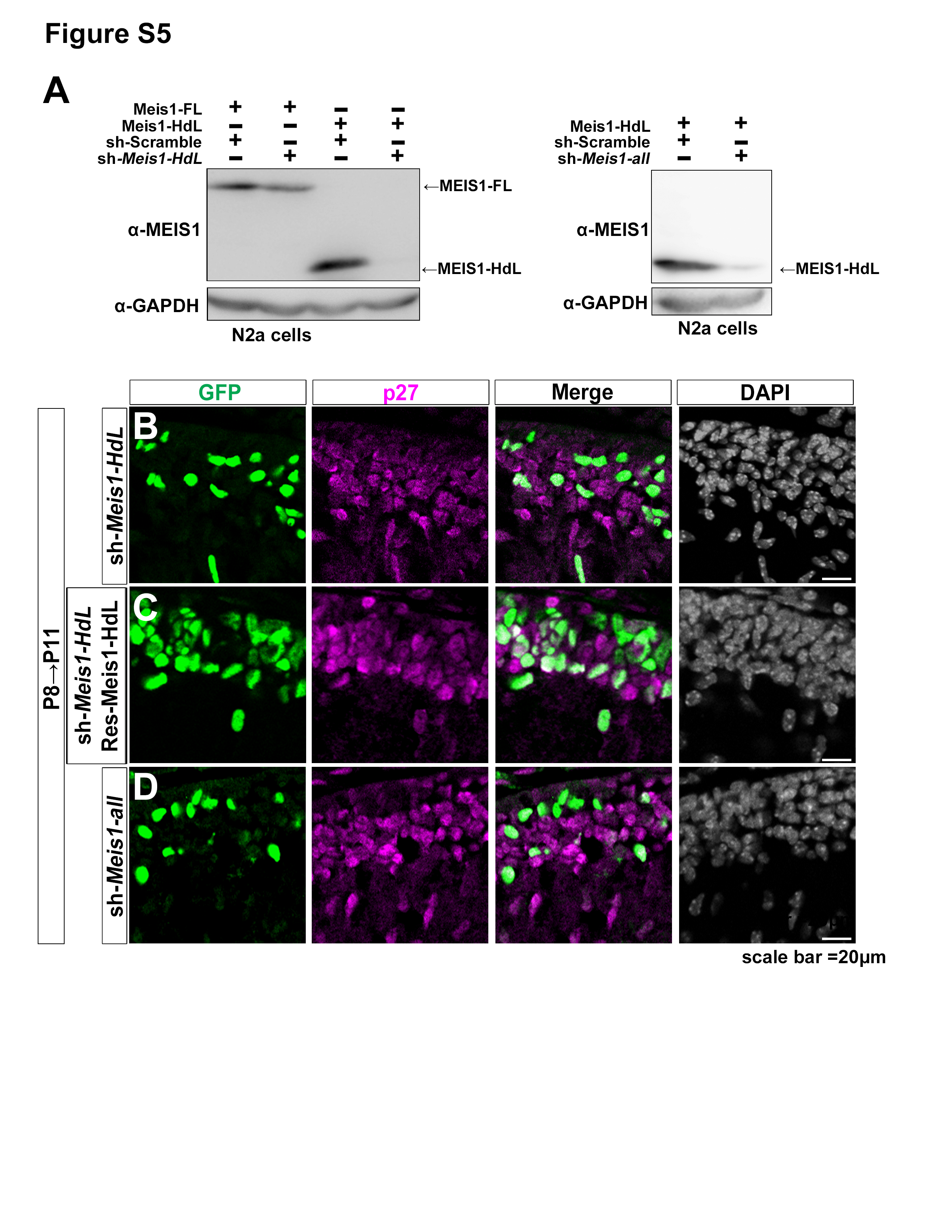

Supplement: S5 Fig — A. Immunoblot analysis validating the specificity and efficacy of the indicated shRNAs in N2a cell lysates. Left panel: Specificity test for sh-Meis1-HdL. The blot shows that sh-Meis1-HdL targets Meis1-HdL for knockdown but does not affect Meis1-FL. Right panel: Efficacy test for sh-Meis1-all. The blot confirms that sh-Meis1-all effectively knocks down Meis1-HdL. B–D. Representative immunofluorescence images of P11 cerebella following in vivo electroporation at P8. Cerebellar sections were immunostained for p27 (magenta). Electroporated cells, identified by co-electroporated H3.1-EGFP (green), were transfected with (B) sh-Meis1-HdL, (C) sh-Meis1-HdL plus a knockdown-resistant rescue construct (Res-Meis1-HdL), or (D) sh-Meis1-all. The data underlying this figure can be found in S5 Data. (TIF) [file pbio.3003897.s005.tif]

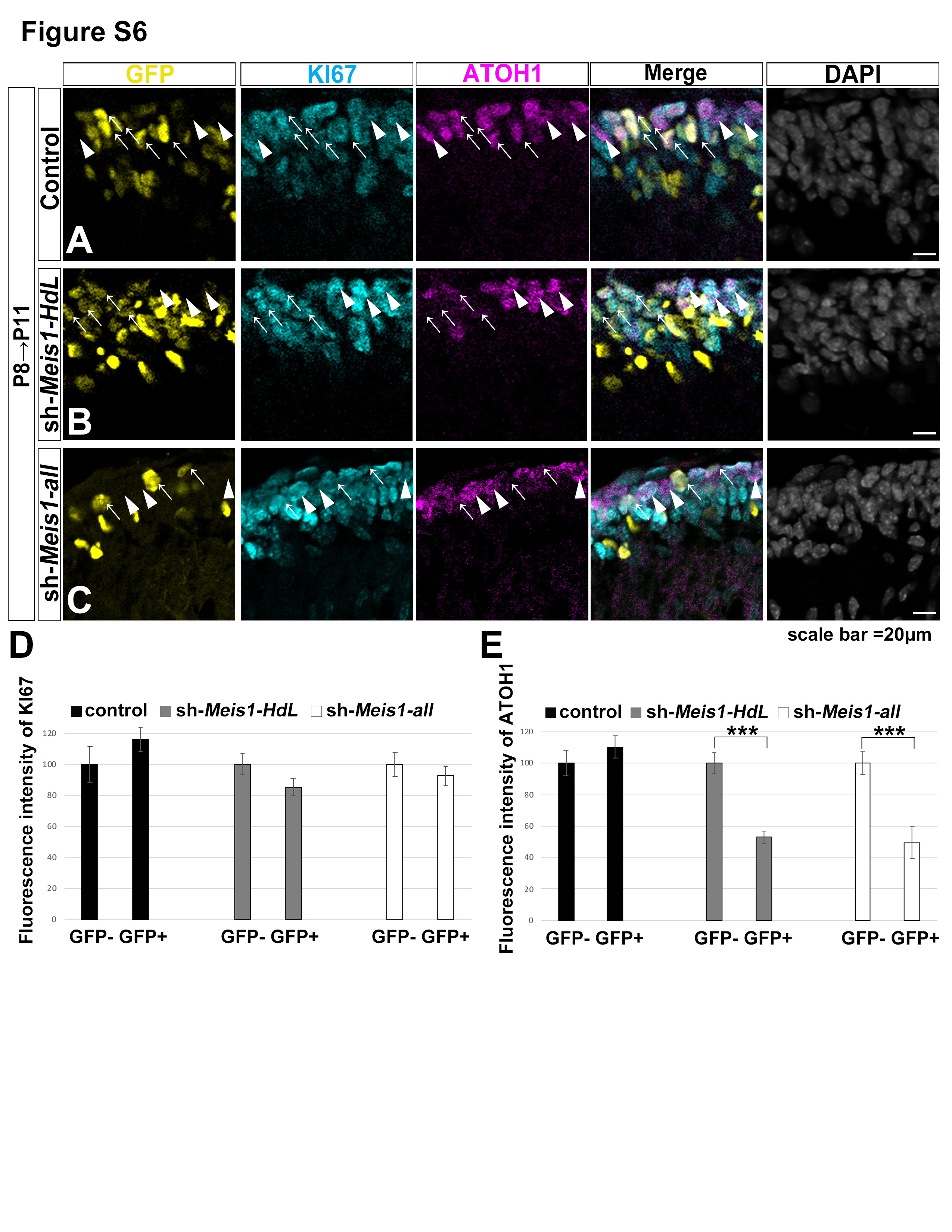

Supplement: S6 Fig — A–C. Representative immunofluorescence images of P11 cerebella following in vivo electroporation at P8. Sections were immunostained for ATOH1 (magenta) and KI67 (cyan). Electroporated cell nuclei are identified by H3.1-EGFP (yellow). The images show cells transfected with a control vector (A), sh-Meis1-HdL (B), or sh-Meis1-all (C). Arrows indicate electroporated (GFP+) cells, and arrowheads indicate surrounding non-electroporated (GFP−) cells. D. Quantification of KI67 fluorescence intensity in electroporated (GFP+) and neighboring non-electroporated (GFP−) cells from the outer EGL, as shown in (A–C). The analysis indicates no significant change in KI67 levels following Meis1 knockdown. E. Quantification of ATOH1 fluorescence intensity in electroporated (GFP+) and neighboring non-electroporated (GFP−) cells from the outer EGL, as shown in (A–C). The results show a significant reduction in ATOH1 levels in cells with Meis1 knockdown. (n = 4 mice for Control and sh-Meis1-HdL; n = 3 mice for sh-Meis1-all). The data underlying this figure can be found in S5 Data. (TIF) [file pbio.3003897.s006.tif]

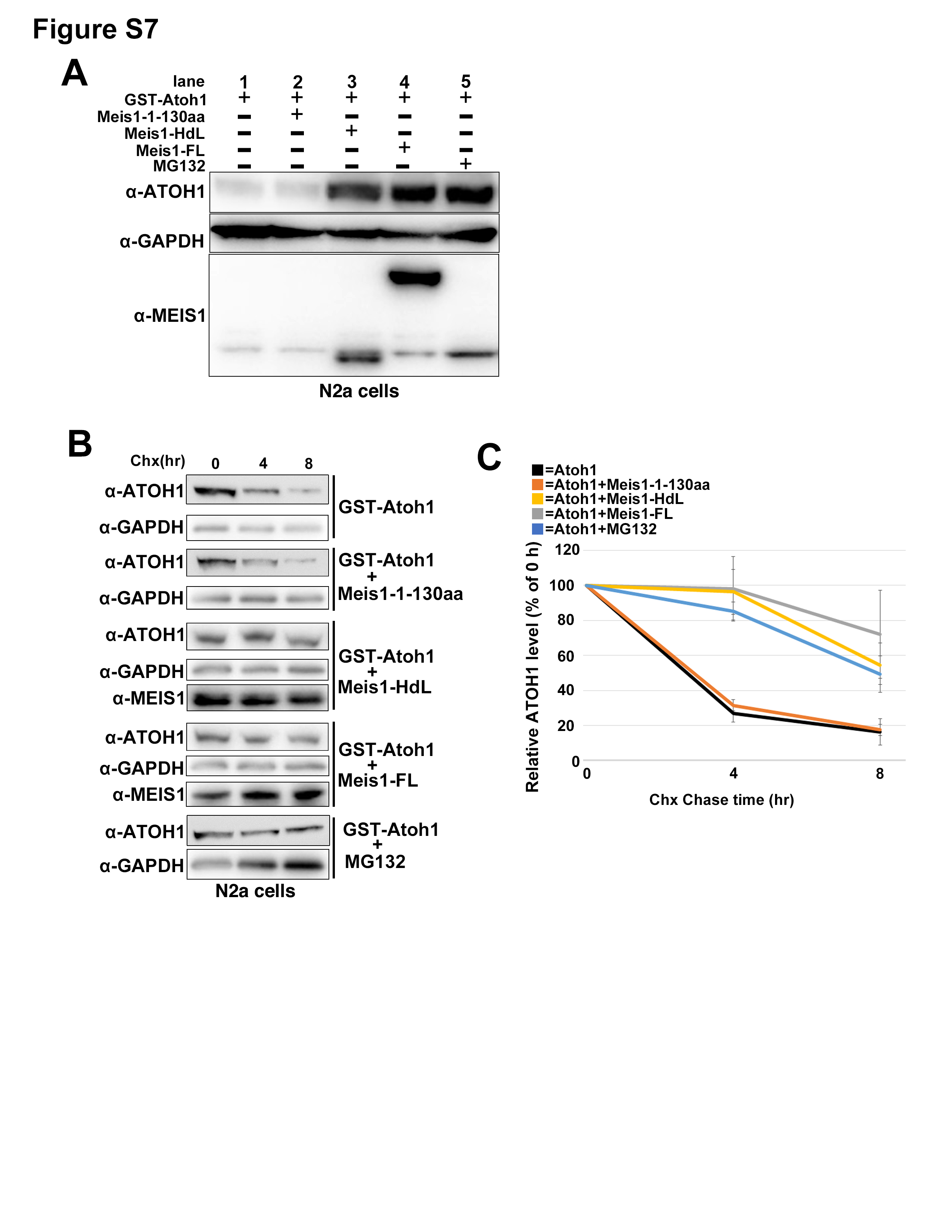

Supplement: S7 Fig — A. Immunoblotting analysis of N2a cell lysates. Cells were transfected with GST-ATOH1 and either no additional plasmid (control), Meis1-1–130aa fragment, Meis1-HdL, or Meis1-FL expression plasmids. Where indicated, cells were treated with MG132 for 6 hours prior to lysis. ATOH1 protein levels were determined using an anti-ATOH1 antibody, with GAPDH as a loading control. Expression of the transfected MEIS1 constructs was confirmed using an anti-MEIS1 antibody. B. Immunoblotting analysis of N2a cell lysates following CHX chase assay. Cells were transfected with GST-ATOH1 and either Meis1-1–130aa fragment, Meis1-HdL, or Meis1-FL expression plasmids. Where indicated, cells were pre-treated with MG132 for 6 hours before CHX administration. Lysates were collected at 0, 4, and 8 hours after CHX treatment. ATOH1 protein levels were determined using an anti-ATOH1 antibody, with GAPDH as a loading control. MEIS1 expression was confirmed by immunoblotting with an anti-MEIS1 antibody. C. Quantification of ATOH1 protein levels from (B), normalized to GAPDH. (n = 4 independent experiments). The data underlying this figure can be found in S5 Data. (TIF) [file pbio.3003897.s007.tif]

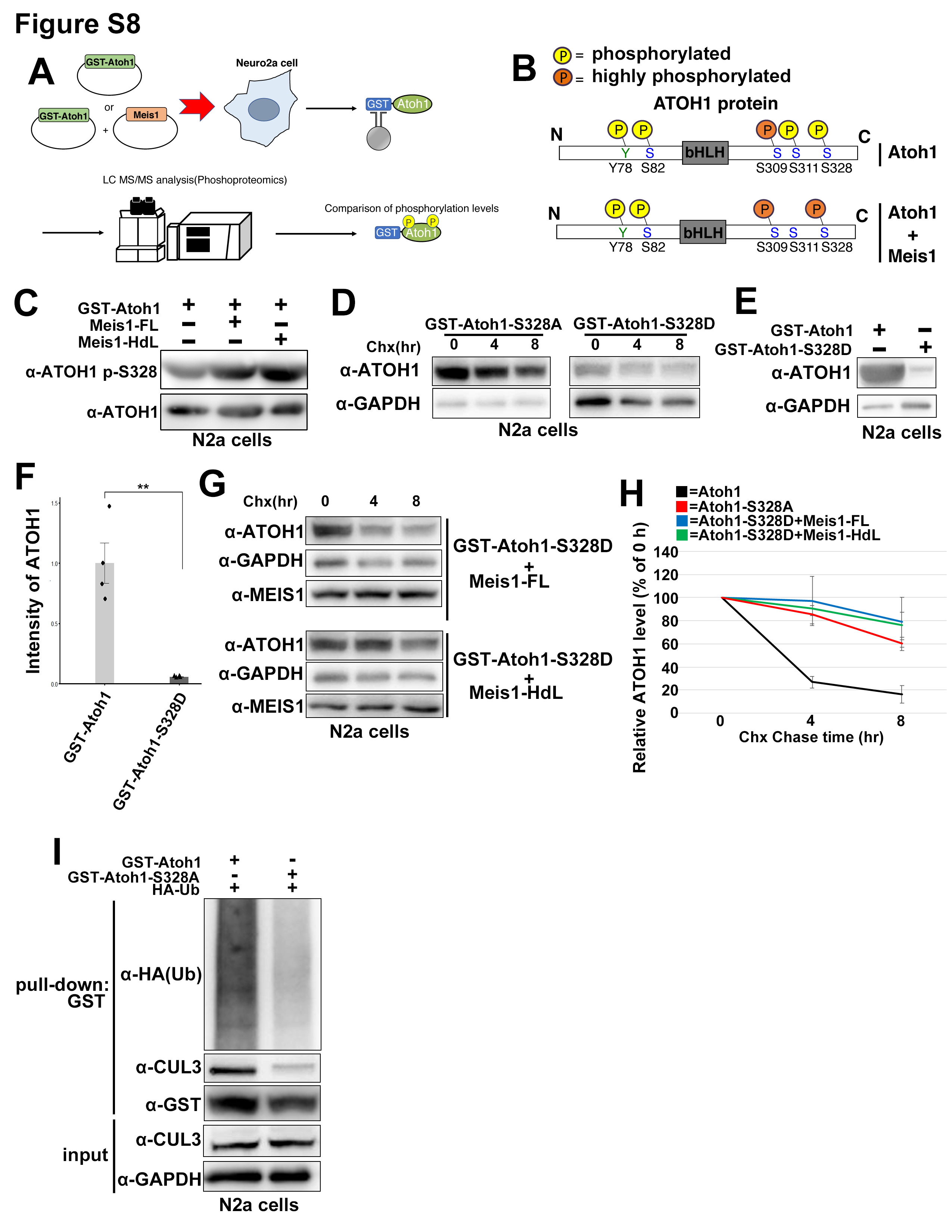

Supplement: S8 Fig — A. Schematic illustration of the proteomic analysis strategy used to identify phosphorylation sites of ATOH1 in N2a cells in the presence or absence of MEIS1. B. Schematic diagram summarizing ATOH1 phosphorylation sites identified by LC–MS/MS analysis. GST-ATOH1 was purified from N2a cells expressing it either alone or with MEIS1. The analysis identified five phosphorylation sites and revealed a marked increase in phosphorylation at the S328 residue in the presence of MEIS1. See S4 Data. C. Immunoblotting analysis of N2a cell lysates. Cells were transfected with GST-ATOH1 alone or in combination with MEIS1-FL or MEIS1-HdL. Samples were loaded to contain equal amounts of total ATOH1. ATOH1-S328 phosphorylation levels were determined by Western blotting with an anti-ATOH1 p-S328 antibody. D. Immunoblotting analysis of N2a cell lysates following CHX chase assay. Cells were transfected with GST-ATOH1-S328A (non-phosphorylated form) or GST-ATOH1-S328D (phosphomimic form) plasmids and harvested at 0, 4, and 8 hours after treatment with CHX. ATOH1 protein levels were detected with an anti-ATOH1 antibody, with GAPDH as a loading control. E. Immunoblotting analysis comparing the baseline steady-state protein levels of WT ATOH1 and ATOH1-S328D. N2a cells were transfected with equal amounts of GST-ATOH1 (wild-type) or GST-ATOH1-S328D plasmids. The blot demonstrates that the phosphomimetic S328D mutant exhibits a markedly lower baseline protein level compared to WT ATOH1. F. Quantification of baseline ATOH1 protein levels from (E), normalized to GAPDH. G. Immunoblotting analysis of N2a cell lysates following CHX chase assay. Cells were transfected with GST-ATOH1-S328D in combination with MEIS1-FL or MEIS1-HdL plasmids and harvested at 0, 4, and 8 hours after treatment with CHX. ATOH1 protein levels were detected with an anti-ATOH1 antibody, with GAPDH as a loading control. MEIS1 expression was confirmed by immunoblotting with an anti-MEIS1 antibody. H. Quantification of ATOH1 p [file pbio.3003897.s008.tif]

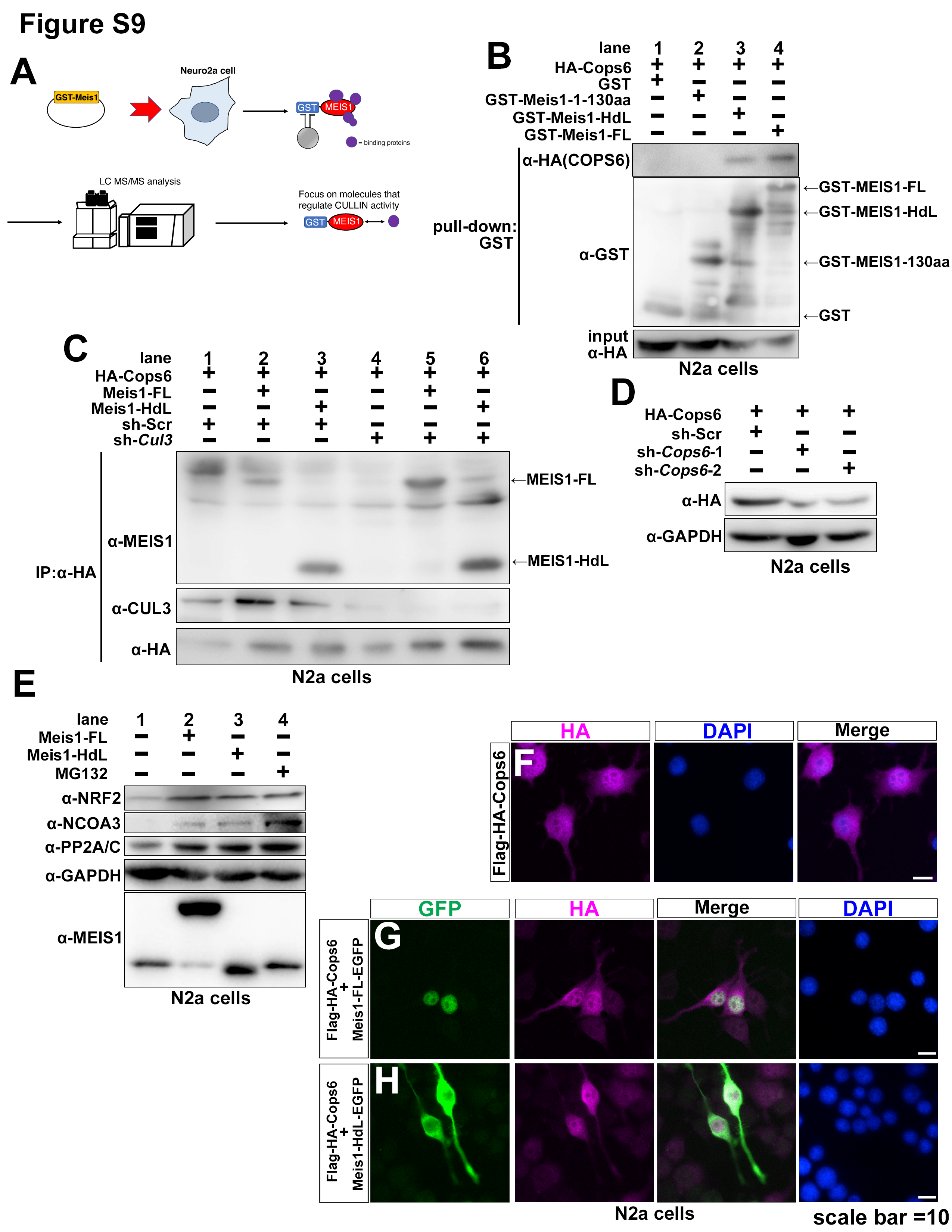

Supplement: S9 Fig — A. Schematic illustrating the proteomic analysis strategy used to identify MEIS1-binding molecules. B. Immunoblotting analysis of GST pull-down fractions from N2a cells transfected with GST, GST-MEIS1-1–130aa fragment, GST-MEIS1-HdL, or GST-MEIS1-FL. All cells were also co-transfected with HA-COPS6. The blot, probed for HA, shows that COPS6 binds to MEIS1-FL and MEIS1-HdL, but not to the N-terminal (1–130aa) fragment or the GST control. C. Co-immunoprecipitation assay demonstrating that the MEIS1–COPS6 interaction is independent of CUL3. HA-COPS6 was immunoprecipitated from N2a cells co-expressing a MEIS1 isoform along with either a control scramble shRNA or an shRNA targeting Cul3. The immunoblots show that the amount of co-precipitated MEIS1 is unaffected by the knockdown of Cul3. D. Immunoblotting analysis of N2a cell lysates. Cells were co-transfected with HA-COPS6 and either a control scramble shRNA or one of two shRNAs targeting Cops6 (sh-Cops6-1 and sh-Cops6-2). The blot was probed with an anti-HA antibody and shows that both shRNAs effectively knock down HA-COPS6 expression compared to the control. E. Immunoblot analysis showing that MEIS1 isoforms stabilize known CUL3 target proteins. N2a cells were transfected with the indicated MEIS1 constructs. Treatment with the proteasome inhibitor MG132 was used as a positive control. The blots show that overexpression of either MEIS1-FL or MEIS1-HdL increases the protein levels of CUL3 targets (NRF2, NCOA3, PP2A/C), similar to the effect of MG132. MEIS1 expression was confirmed by immunoblotting with an anti-MEIS1 antibody. F–H. Representative immunofluorescence images showing the subcellular localization of HA-COPS6 in N2a cells. Cells were transfected with HA-COPS6 alone (F), or co-transfected with HA-COPS6 and MEIS1-FL-EGFP (G), or MEIS1-HdL-EGFP (H). Cells were immunostained for HA (magenta), GFP (green, to visualize MEIS1-EGFP fusion proteins), and DAPI (blue, for nuclei). The data underlying this figure can be [file pbio.3003897.s009.tif]

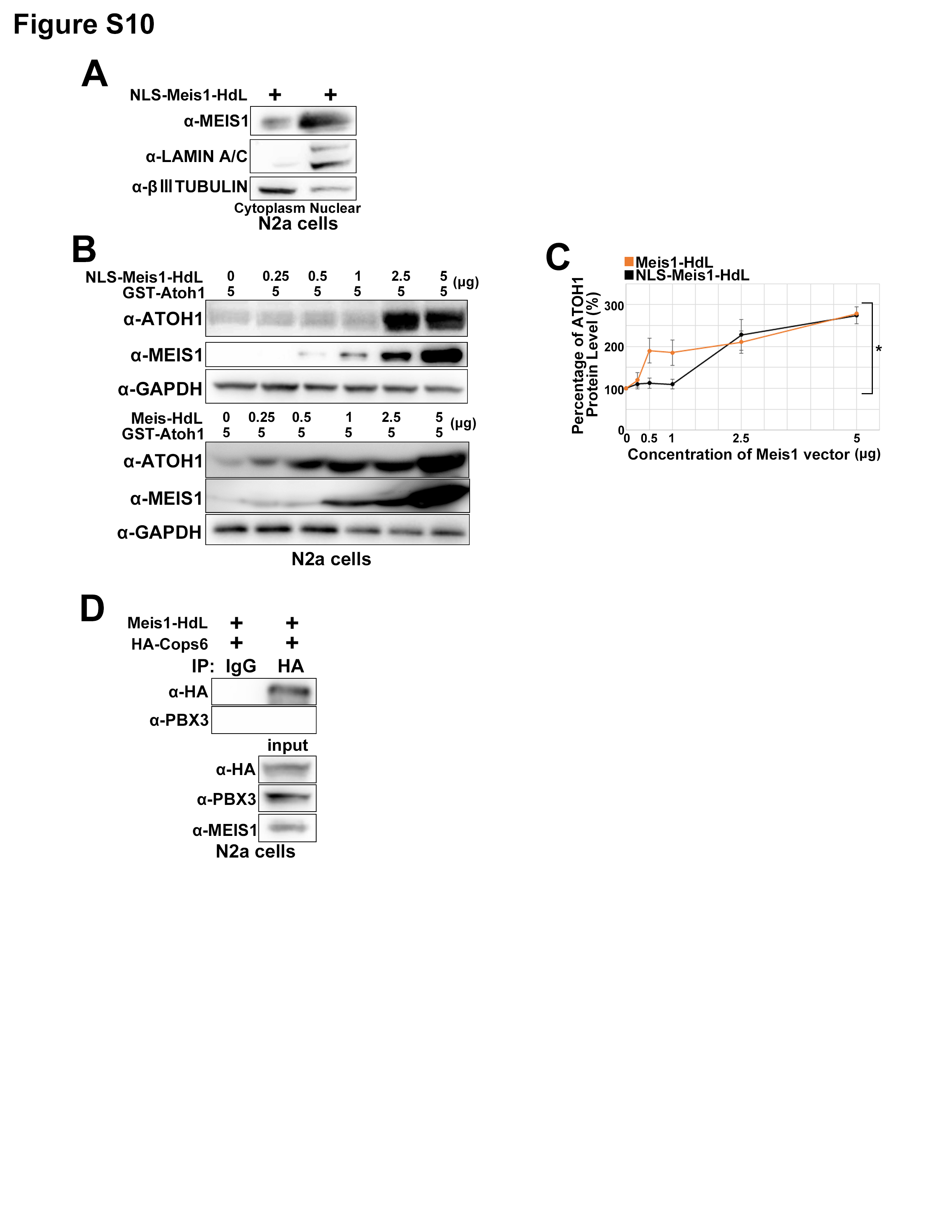

Supplement: S10 Fig — A. Immunoblot of cytoplasmic and nuclear fractions from N2a cells transfected with NLS-MEIS1-HdL. The blot shows that while NLS-MEIS1-HdL is strongly localized to the nuclear fraction, some protein is also detectable in the cytoplasmic fraction. Lamin A/C and βIII-tubulin serve as markers for the nuclear and cytoplasmic fractions, respectively. B. Dose-dependent stabilization of GST-ATOH1 by non-NLS MEIS1-HdL and NLS-MEIS1-HdL. N2a cells were co-transfected with a constant amount of GST-ATOH1 and increasing amounts (0–5 µg) of the MEIS1-HdL or NLS-MEIS1-HdL expression plasmid. Cell lysates were immunoblotted for the indicated proteins. The non-NLS MEIS1-HdL blot is reproduced from Fig 6H for direct comparison with NLS-MEIS1-HdL. C. Quantification of ATOH1 protein levels from (B). To evaluate the impact of subcellular localization, the stabilization efficacy of NLS-MEIS1-HdL was compared with that of non-NLS MEIS1-HdL. The non-NLS MEIS1-HdL quantification is reproduced from Fig 6I for direct comparison. Statistical significance between the overall dose-dependent stabilization effects of non-NLS MEIS1-HdL and NLS-MEIS1-HdL was determined using two-way ANOVA. D. Co-immunoprecipitation assay in N2a cells expressing HA-COPS6 and MEIS1-HdL. HA-COPS6 was immunoprecipitated using an anti-HA antibody, and the resulting precipitates were then immunoblotted for HA and PBX3 to detect a potential interaction. Note that PBX3 was not detected in the precipitates, indicating its lack of interaction with the MEIS1-HdL/CSN complex. (n = 4 independent experiments). The data underlying this figure can be found in S5 Data. (TIF) [file pbio.3003897.s010.tif]

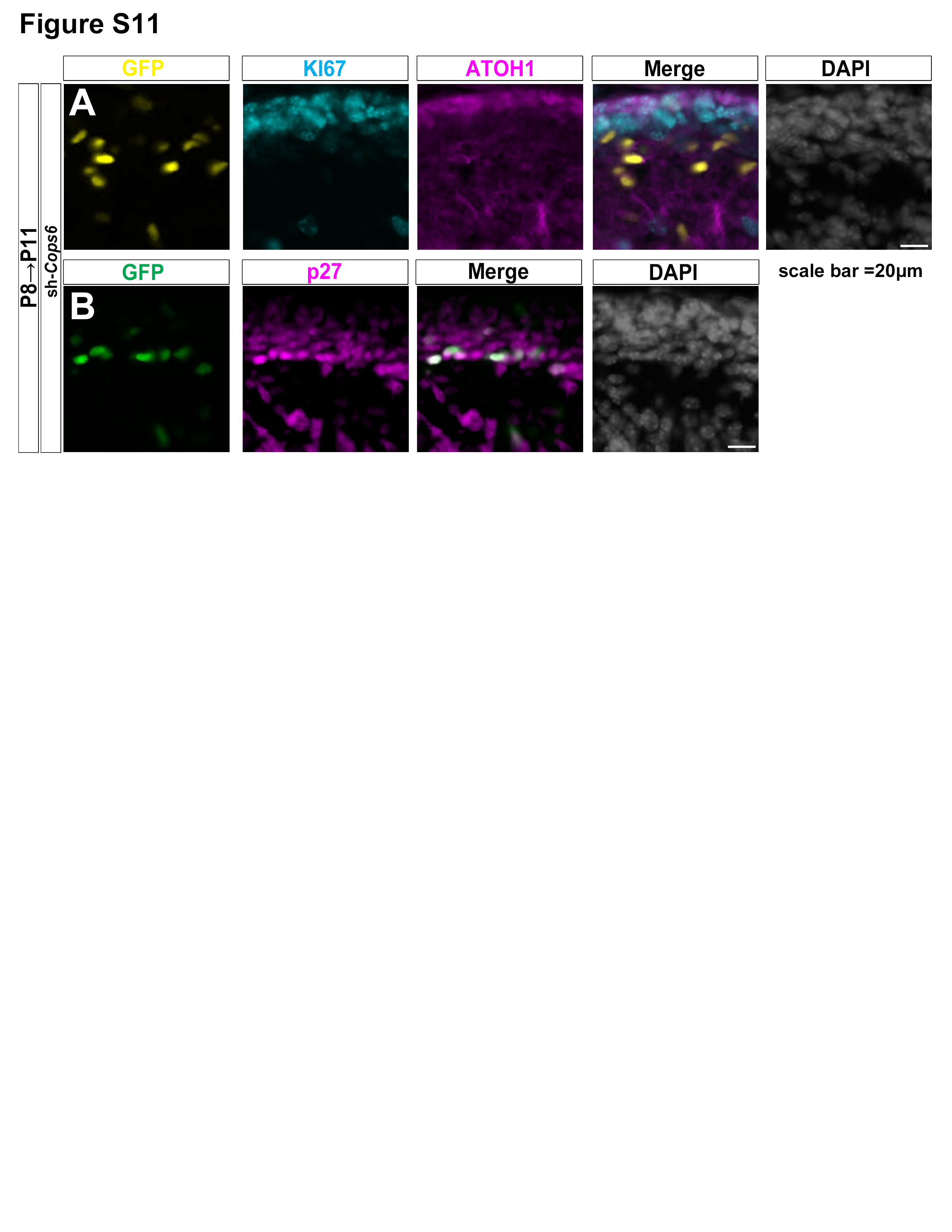

Supplement: S11 Fig — A. Representative immunofluorescence images of P11 cerebella following in vivo electroporation at P8. Sections were immunostained for ATOH1 (magenta) and KI67 (cyan). Electroporated cells are identified by co-electroporated H3.1-EGFP (yellow). These images show GCPs electroporated with sh-Cops6. B. Representative immunofluorescence images of P11 cerebella following in vivo electroporation at P8. Sections were immunostained for p27 (magenta). Electroporated cells are identified by co-electroporated H3.1-EGFP (green). These images show GCPs electroporated with sh-Cops6. The data underlying this figure can be found in S5 Data. (TIF) [file pbio.3003897.s011.tif]
