# Supplementary material for: Meis1 isoform diversity orchestrates neural progenitor differentiation by regulating ATOH1 degradation at distinct subcellular compartments
Source: PLoS Biol. 2026 Jul 13;24(7):e3003897. doi: 10.1371/journal.pbio.3003897 (PMC13379096; doi:10.1371/journal.pbio.3003897)

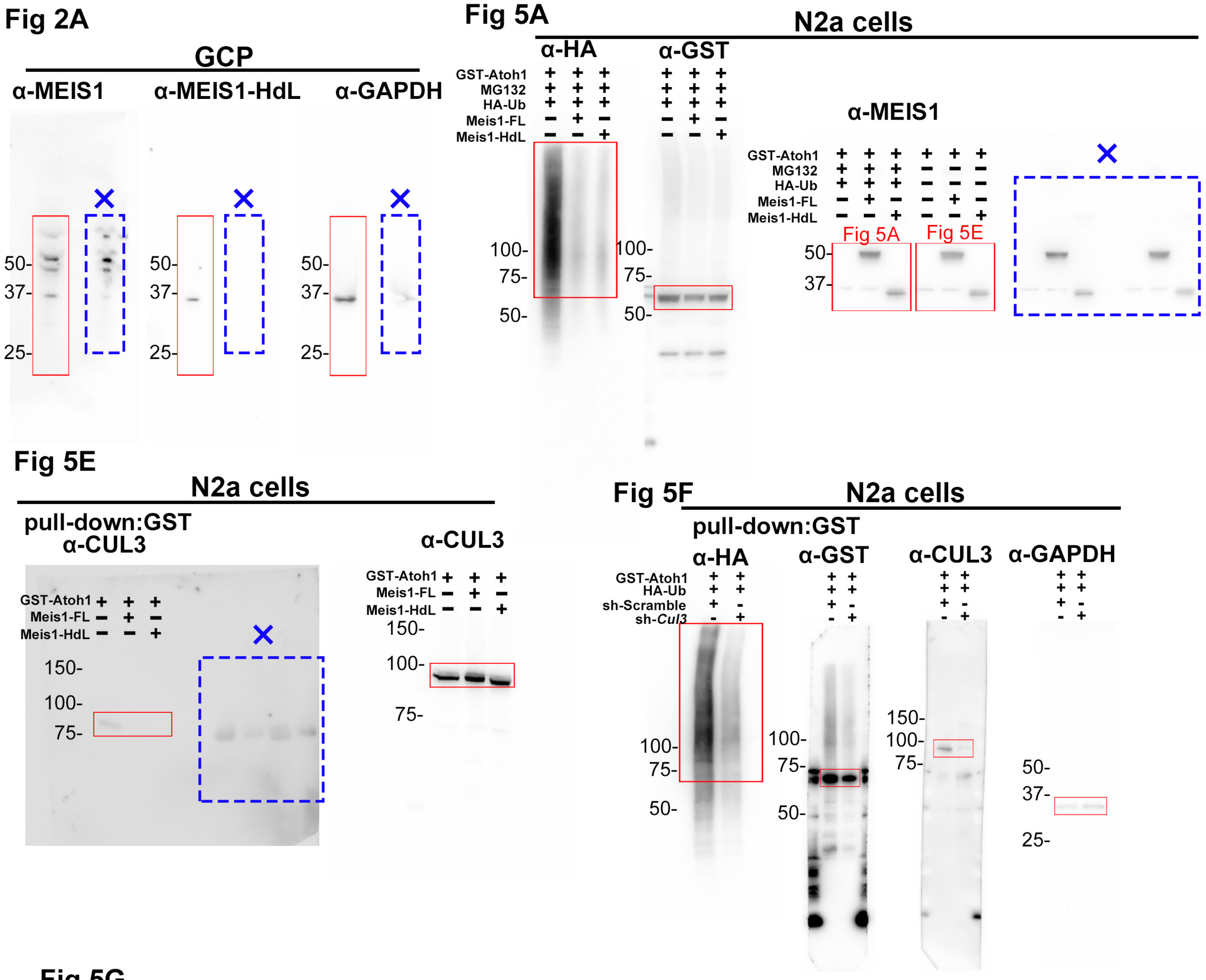

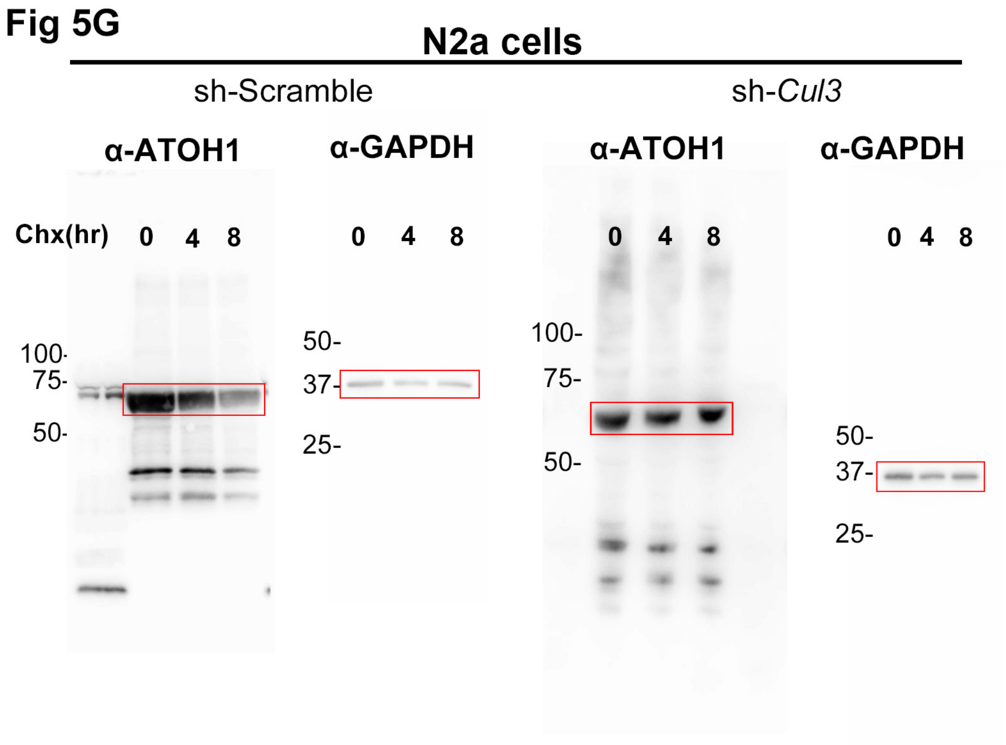

Red boxes indicate the regions used in the final figure.

Blue dashed boxes with X indicate lanes or regions not used in the final figure.

Fig 6B

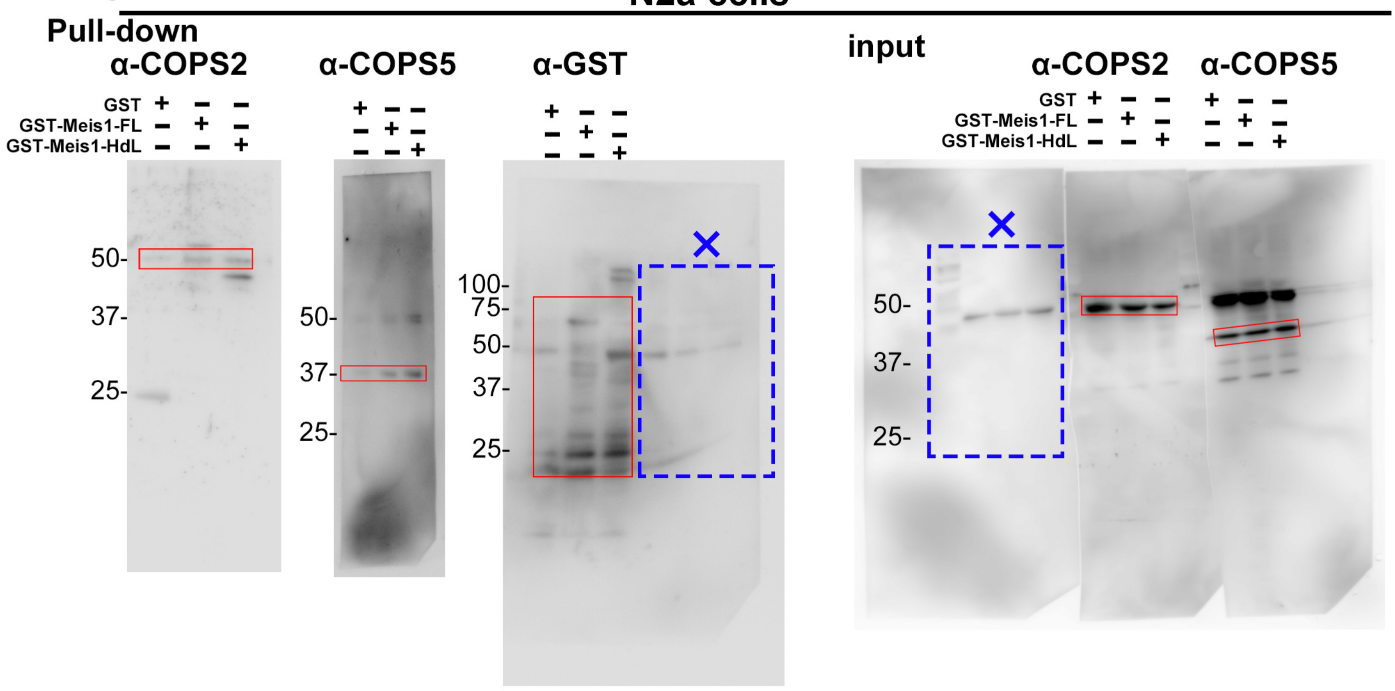

Fig 6C

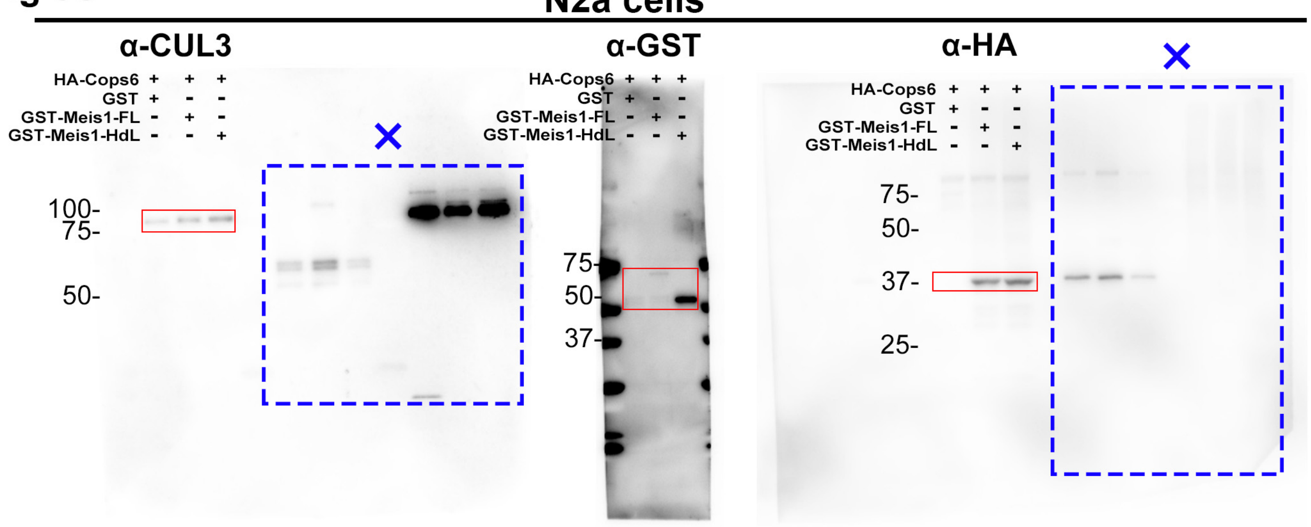

Fig 6D

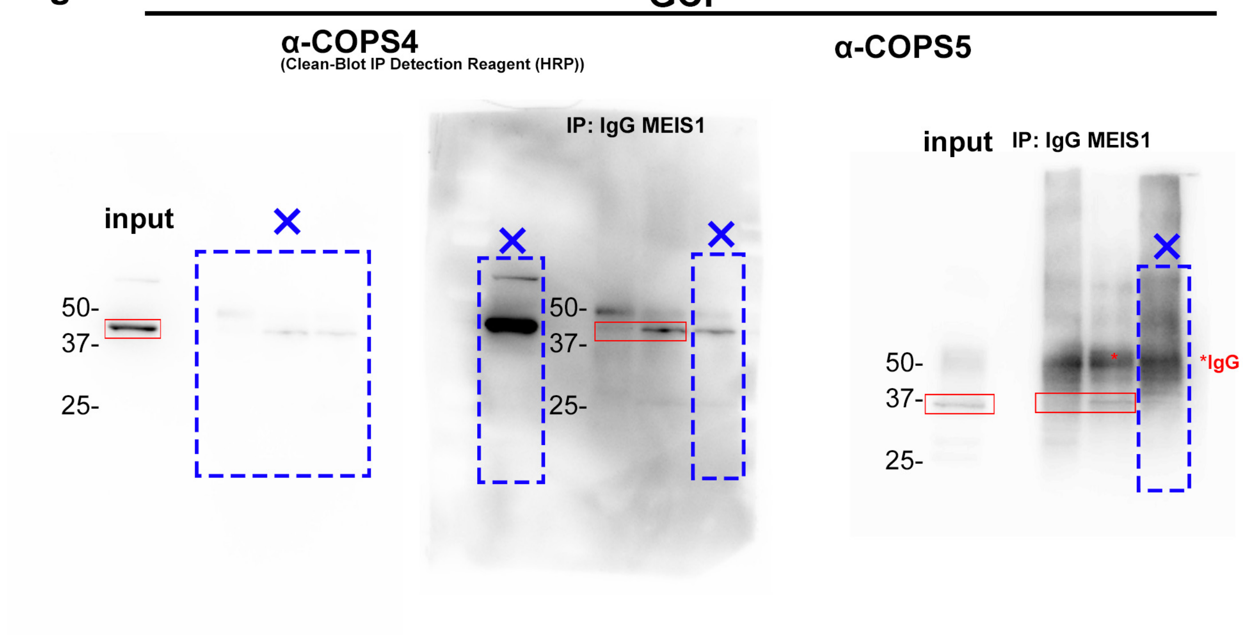

Fig 6E

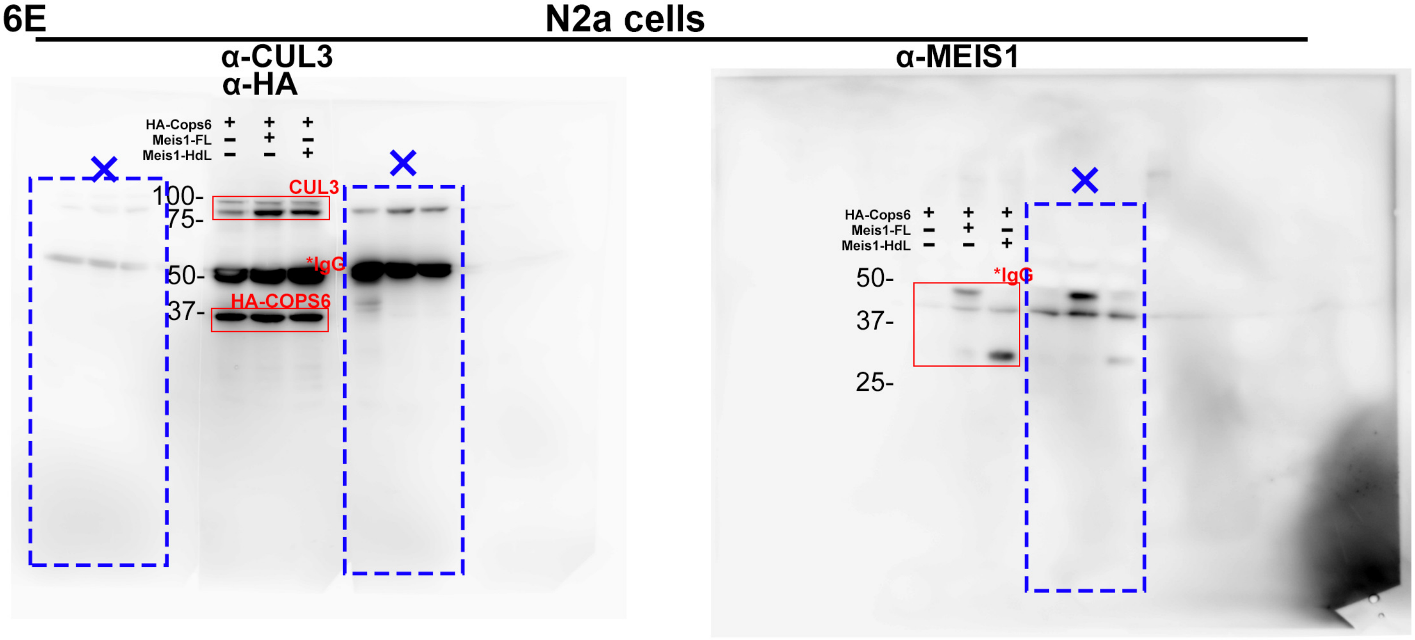

Fig 6G

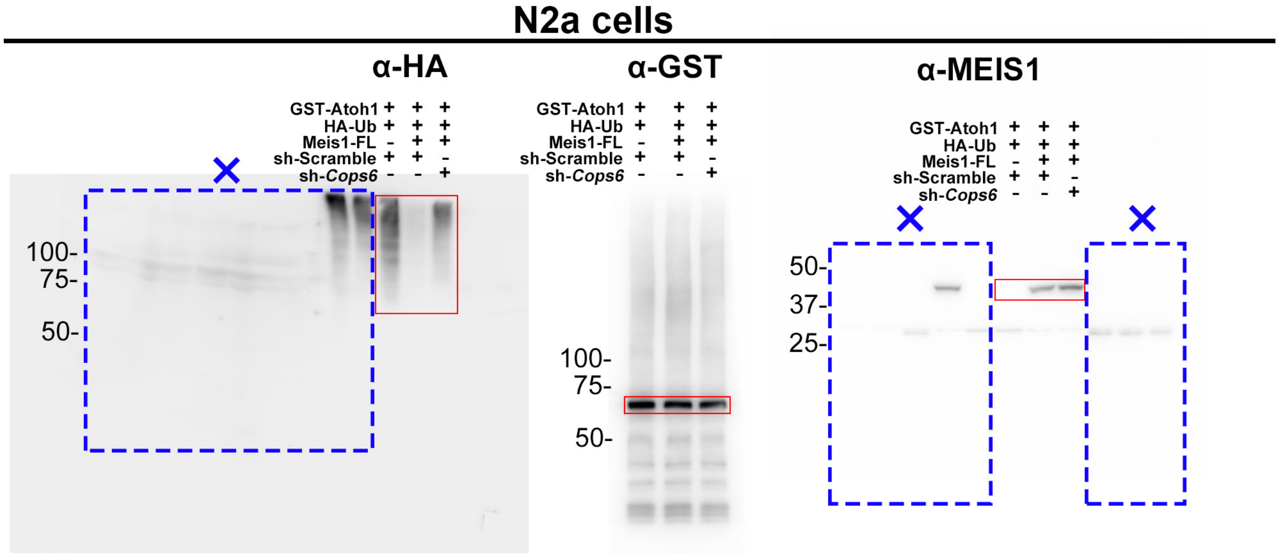

Fig 6H

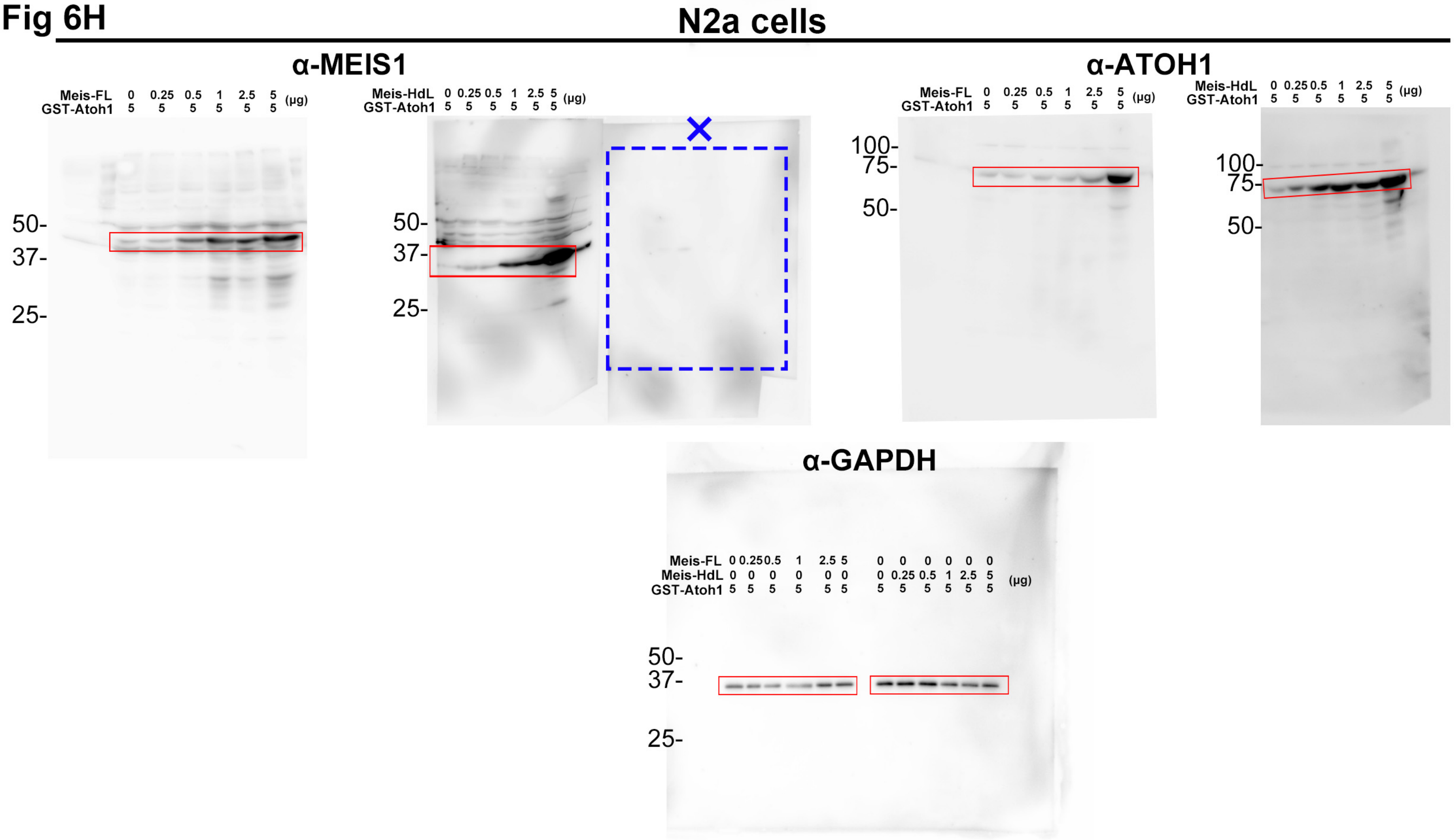

Fig 6J

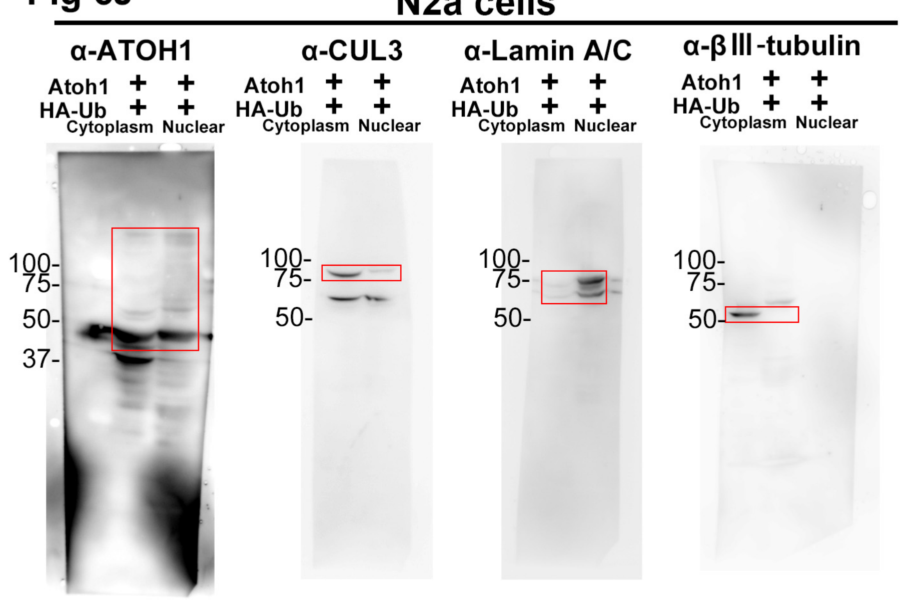

Fig S2A

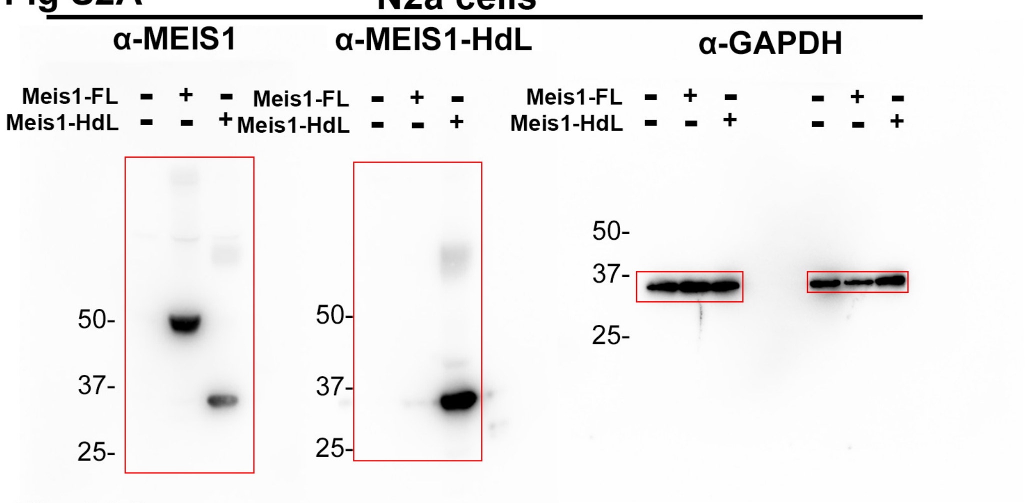

Fig S2B

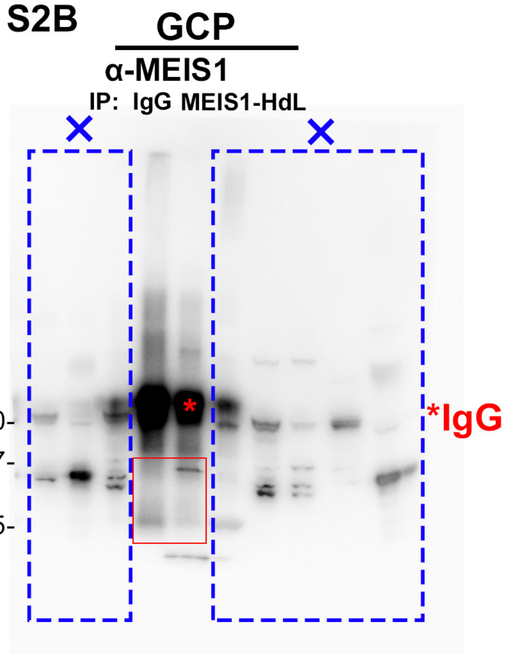

Fig S2C

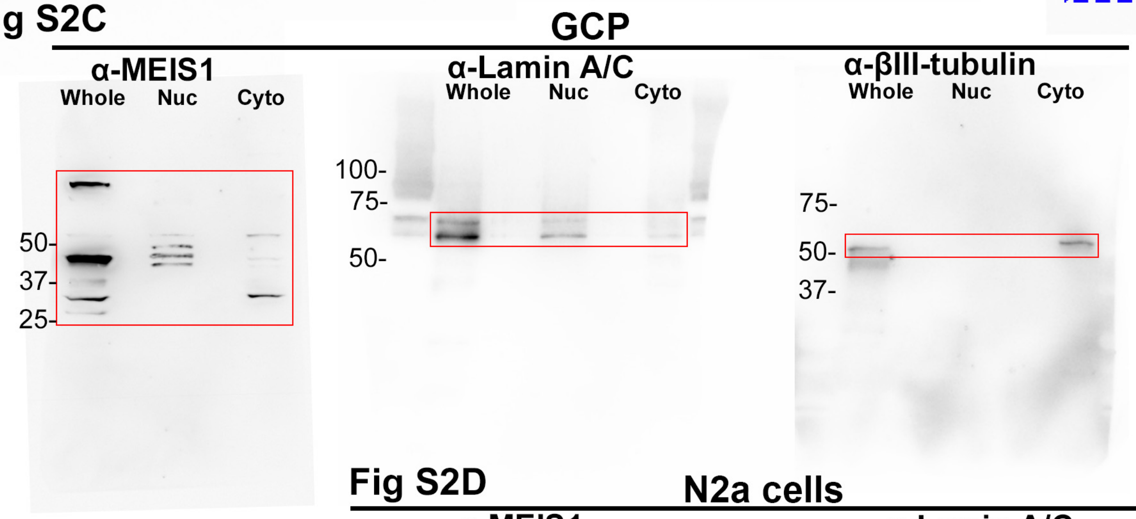

Fig S2D

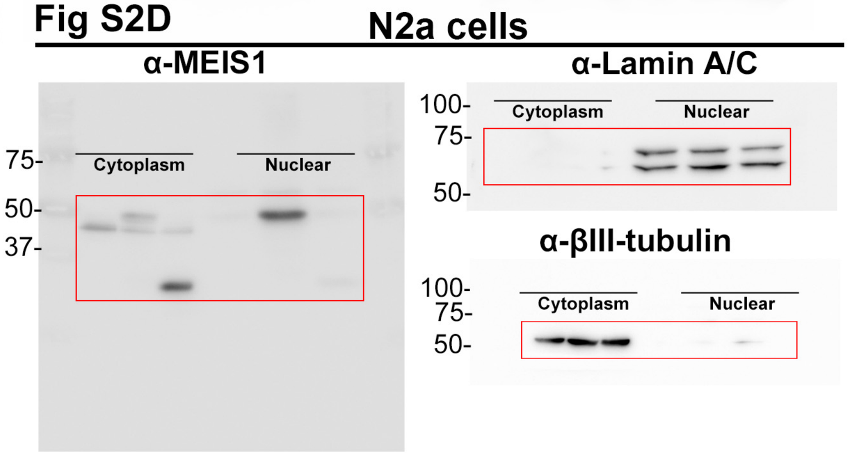

Fig S5A

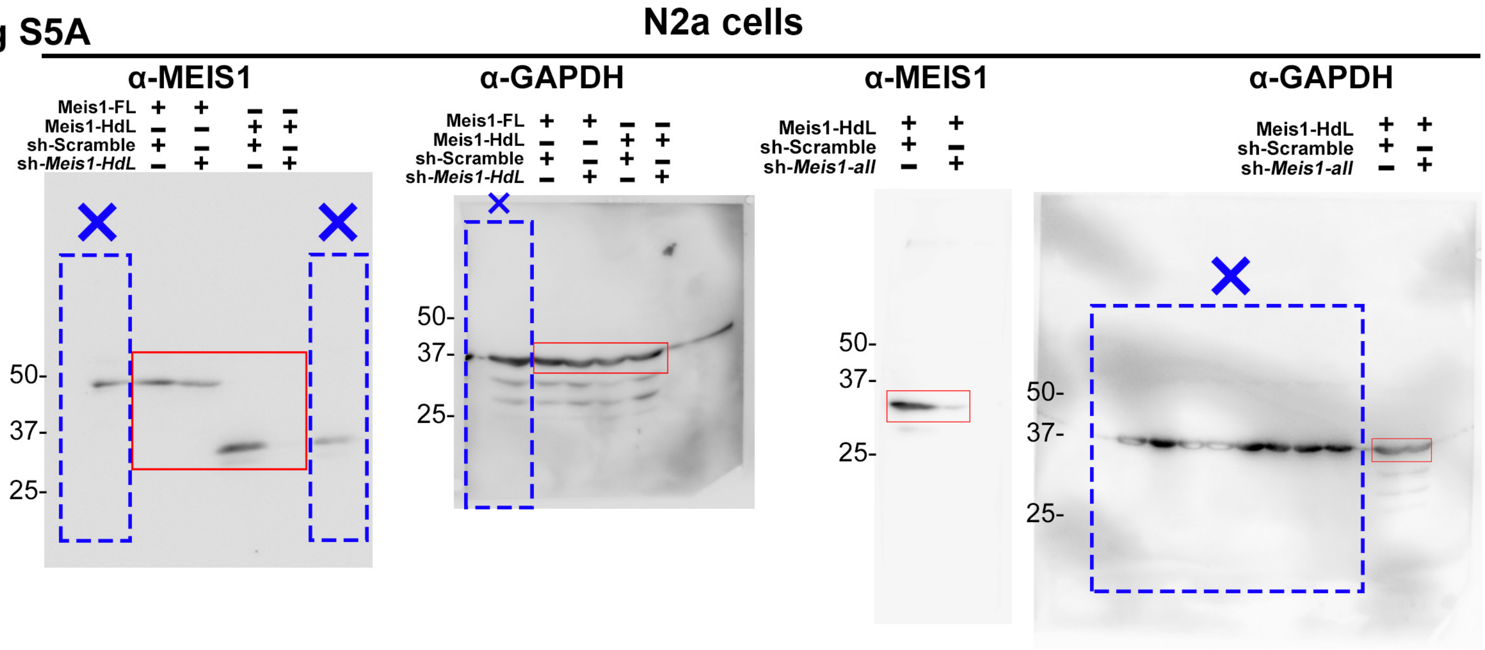

Fig S7A

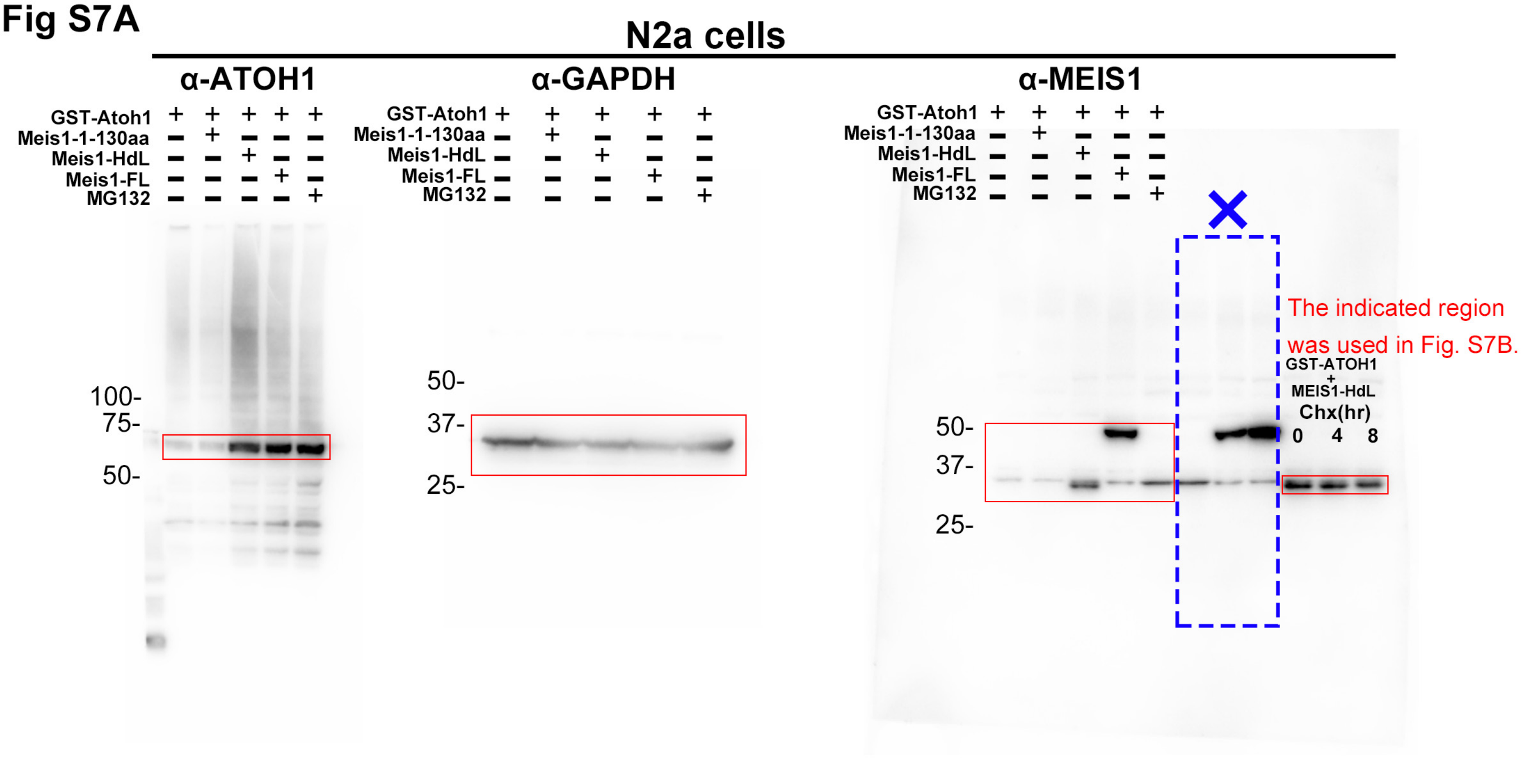

Fig S7B

N2a cells

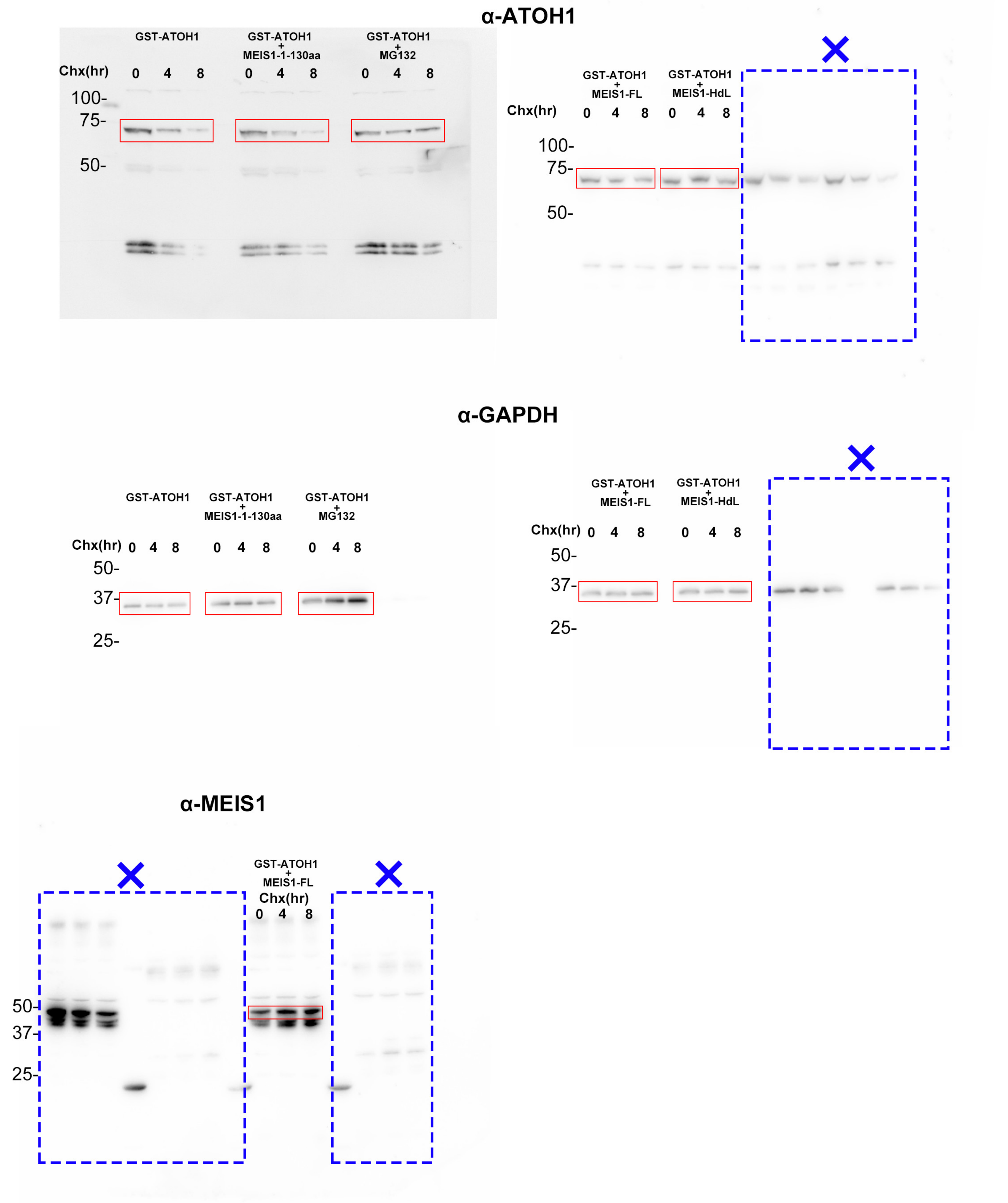

Fig S8C

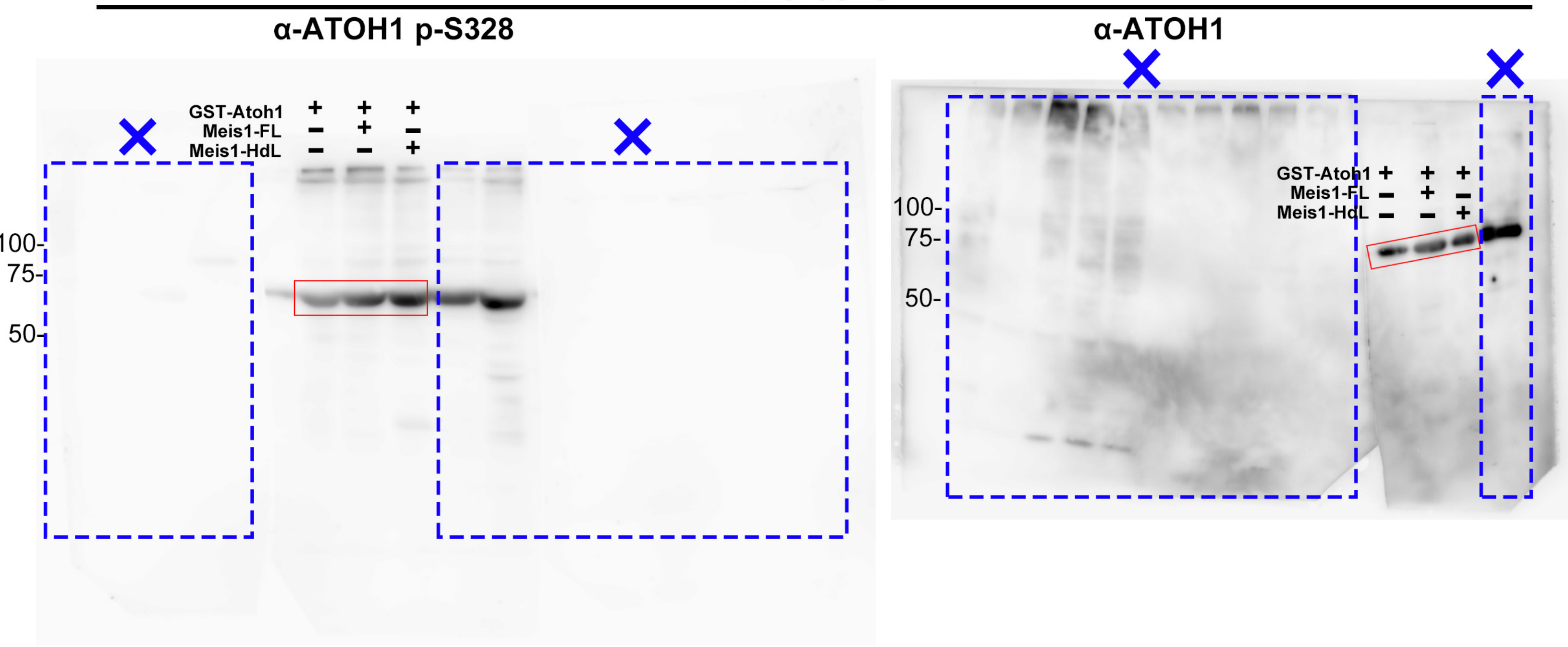

Fig S8D,G

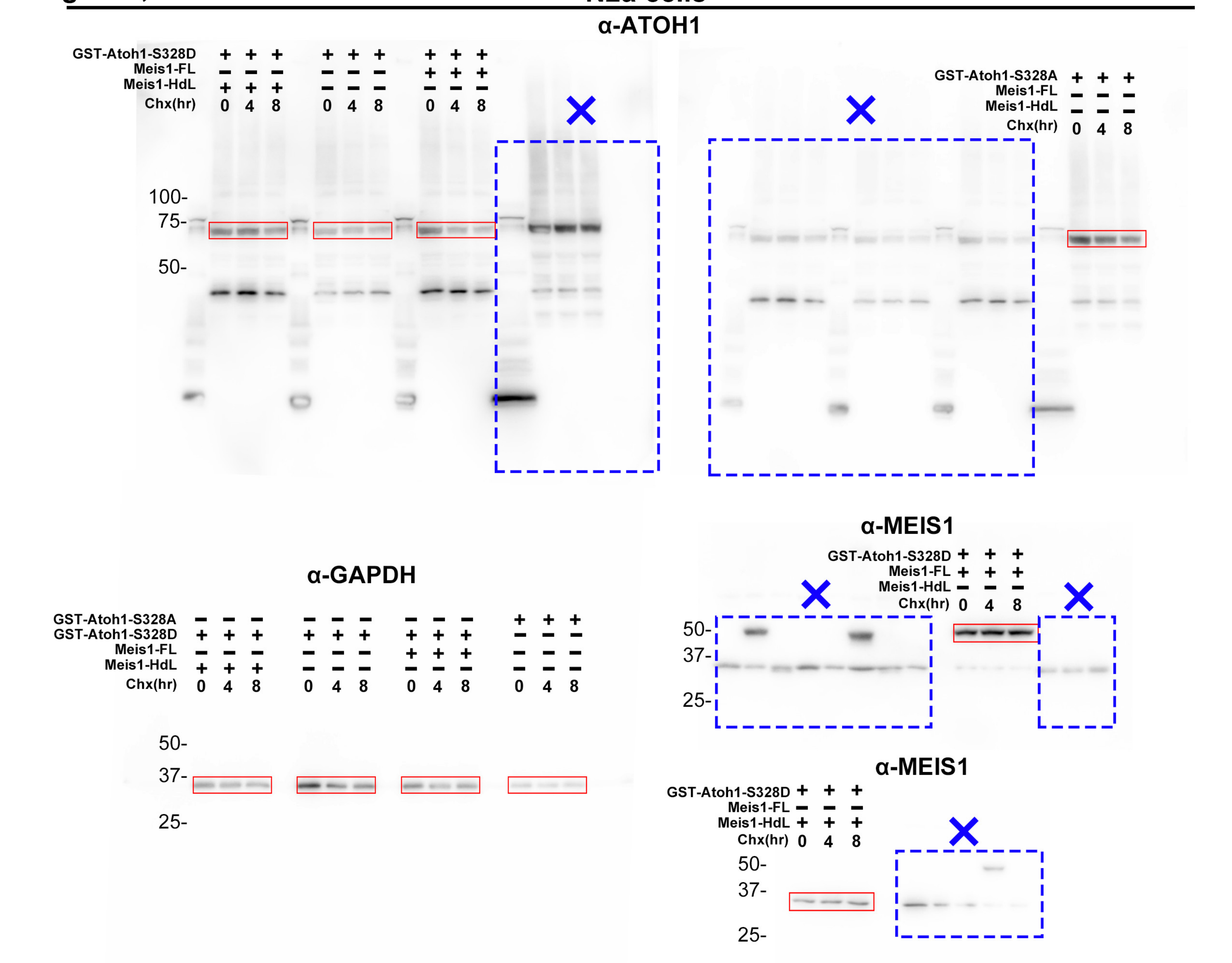

Fig S8E

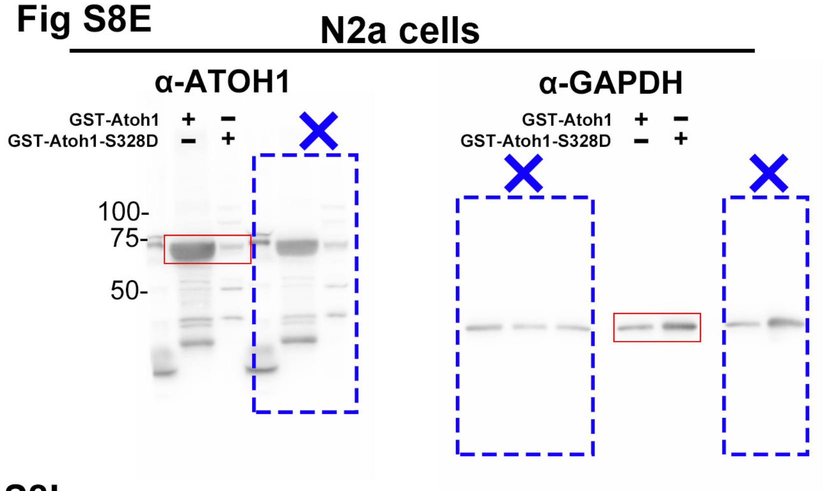

Fig S8I

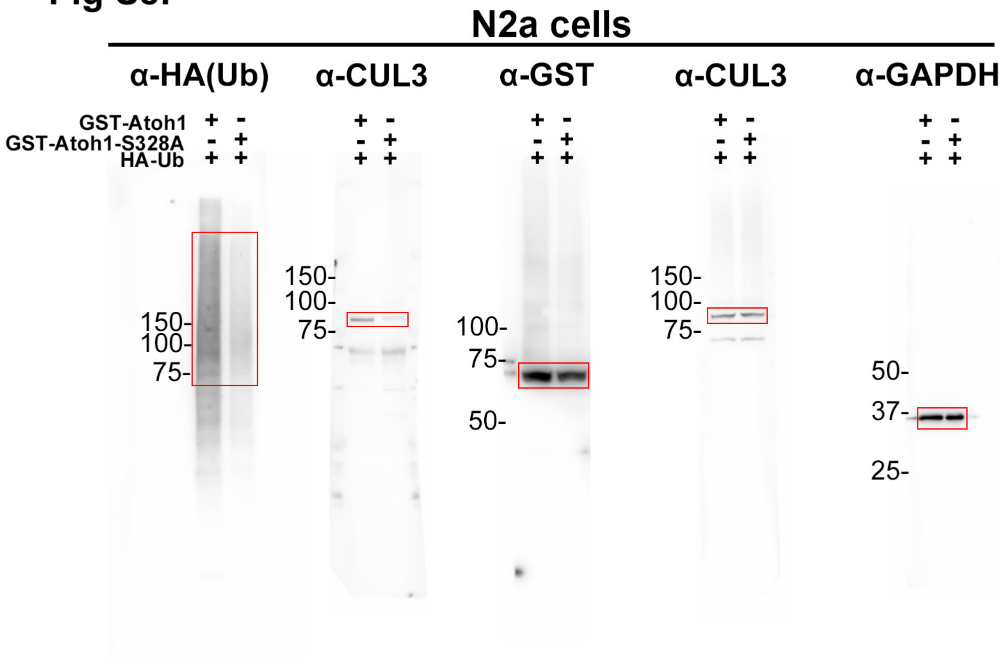

Fig S9B

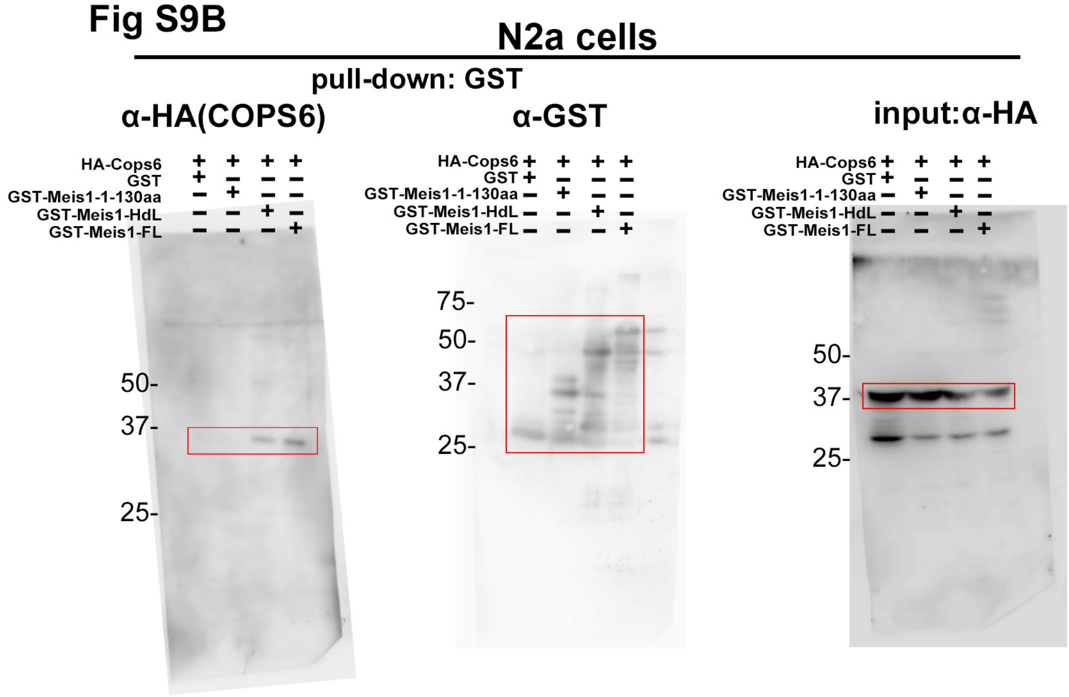

Fig S9C

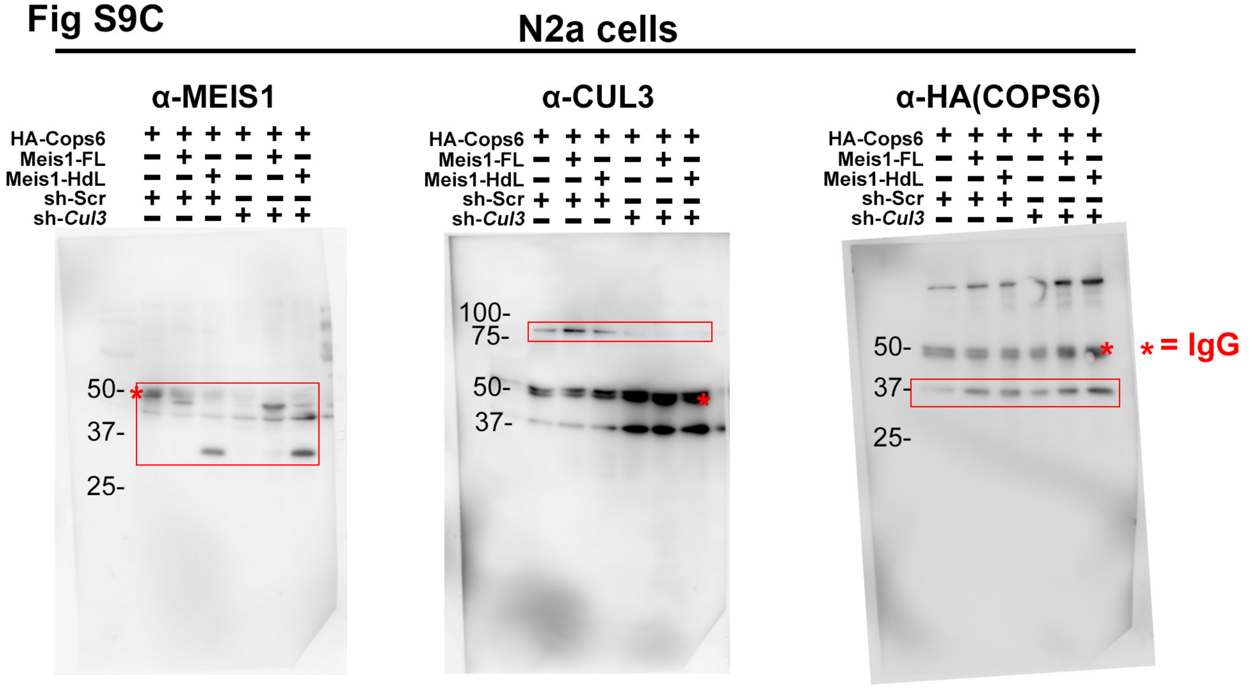

Fig S9D

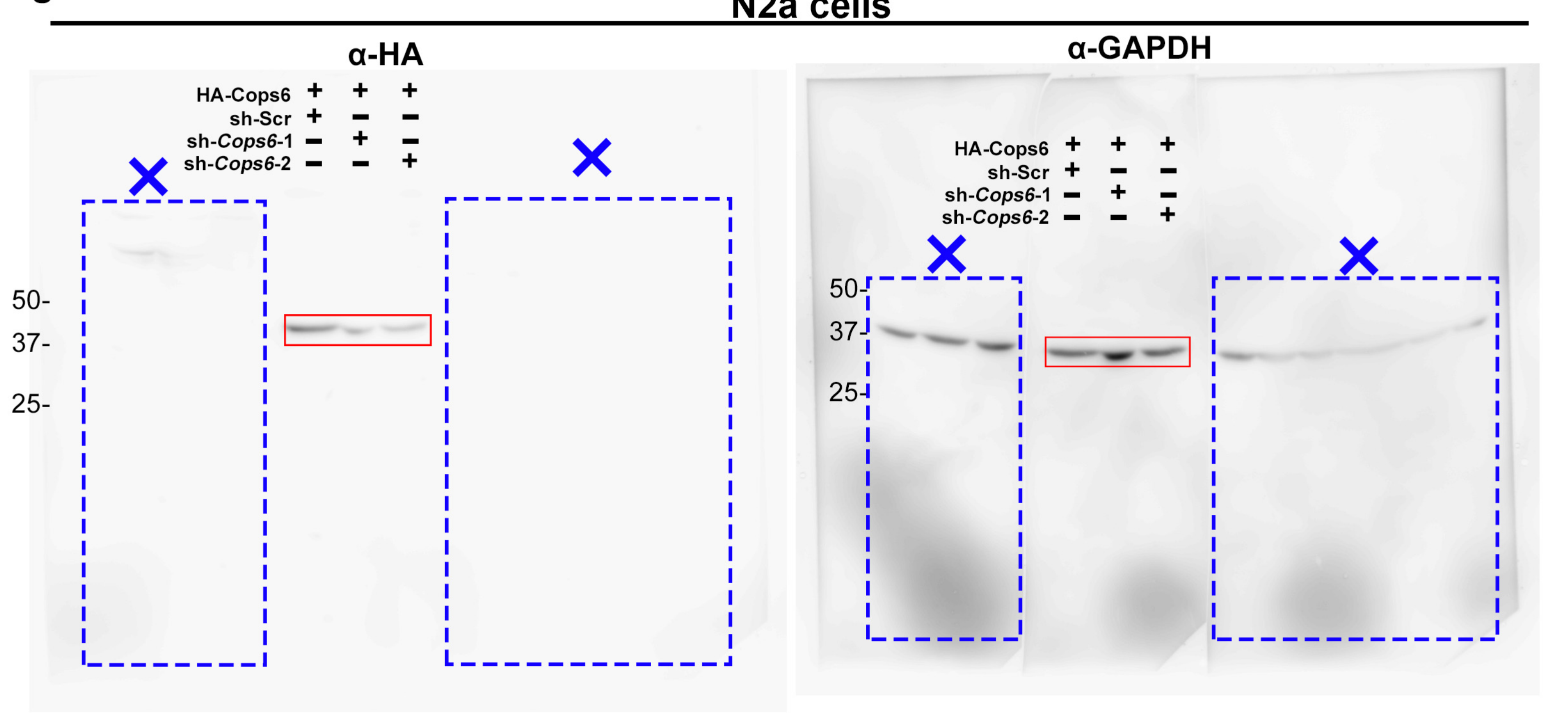

Fig S9E

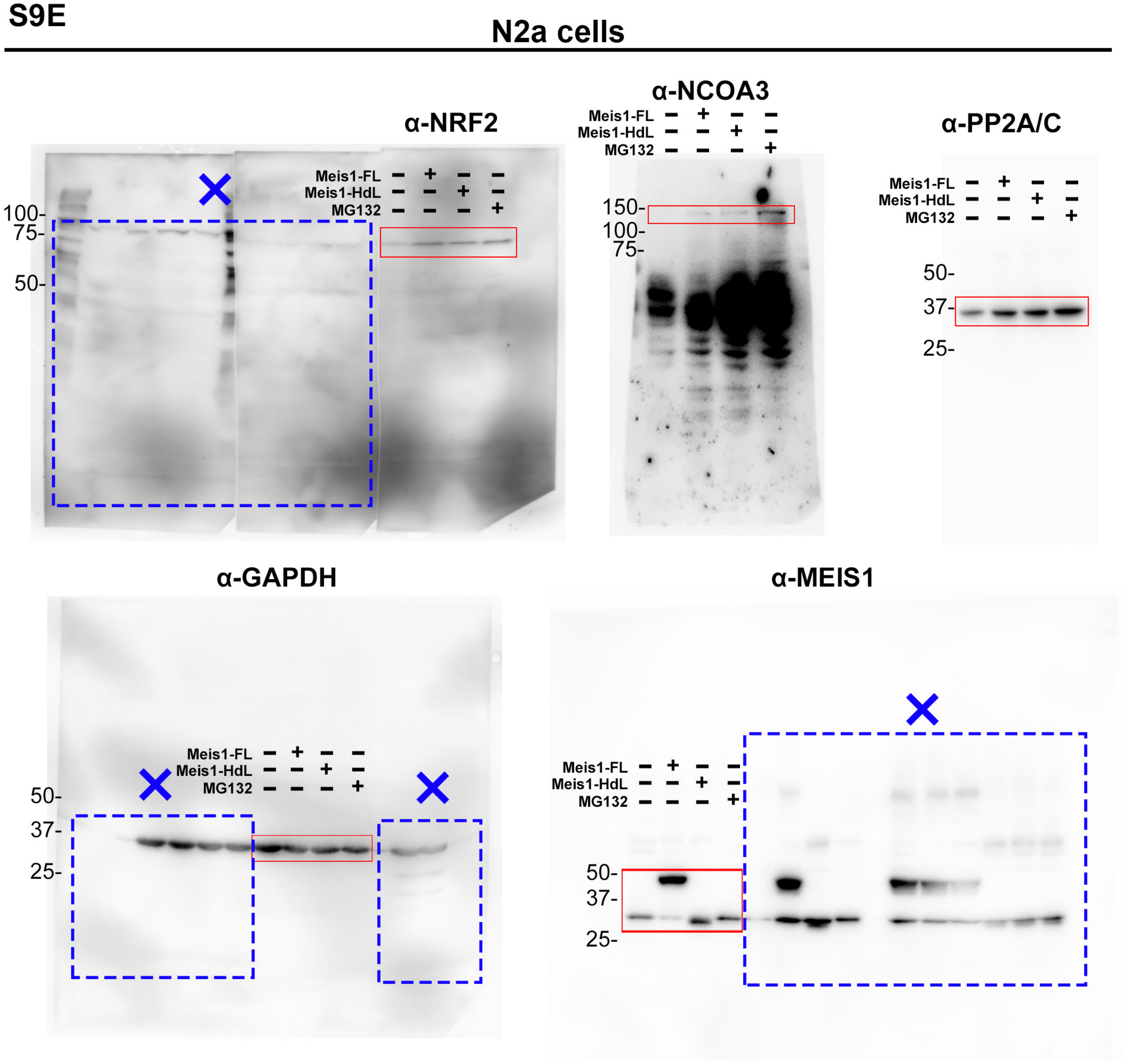
N2a cells
α-NRF2
Meis1-FL − + − −
Meis1-HdL − − + −
MG132 − − − +
100-
75-
50-
α-NCOA3
150-
100-
75-
α-PP2A/C
50-
37-
25-
α-GAPDH
50-
37-
25-
α-MEIS1
50-
37-
25-

Fig S10A

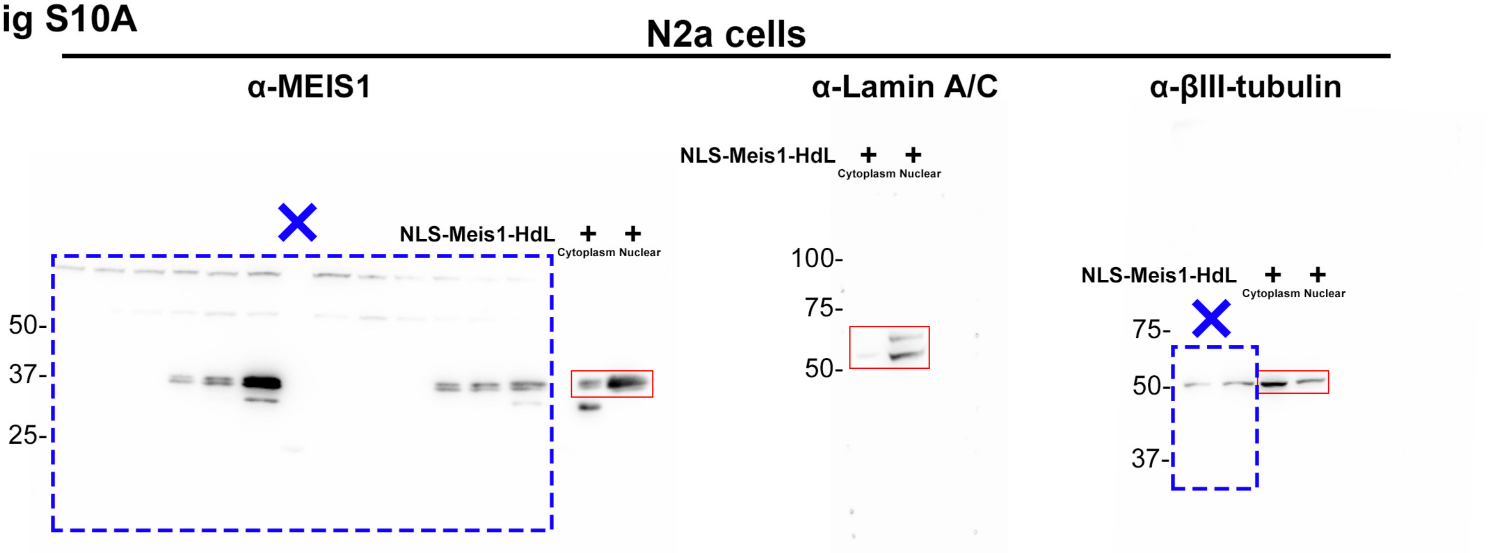

Fig S10B

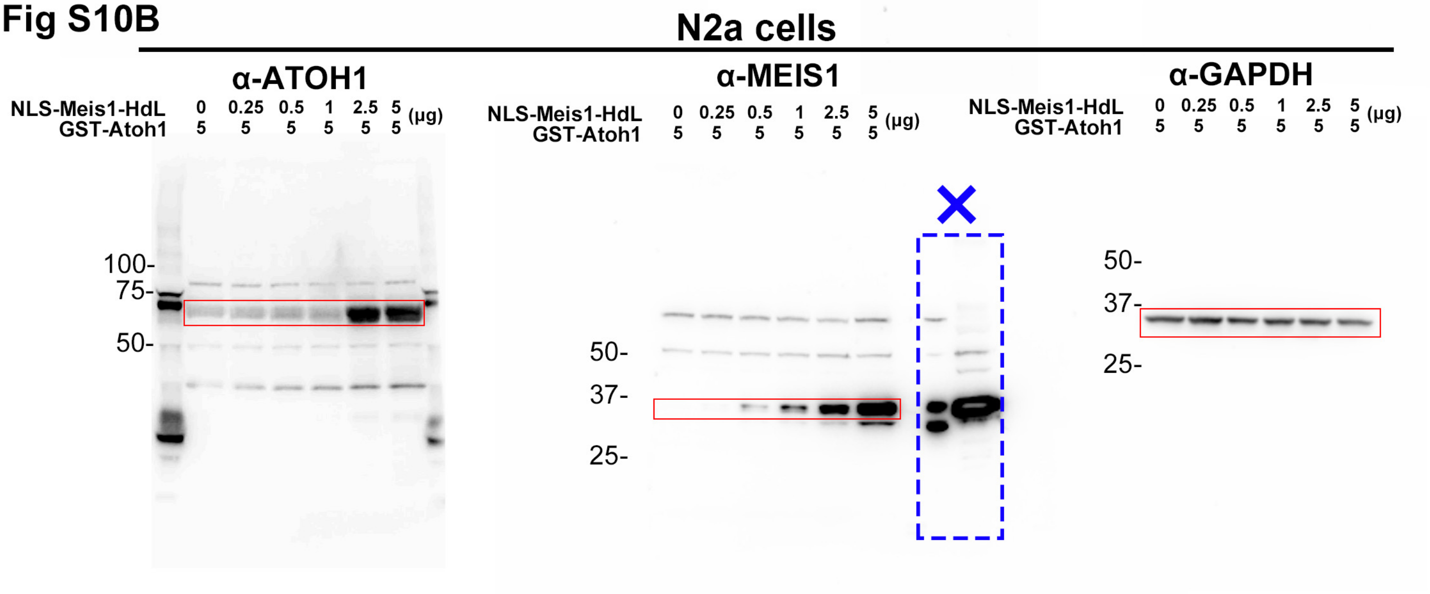

Fig S10D

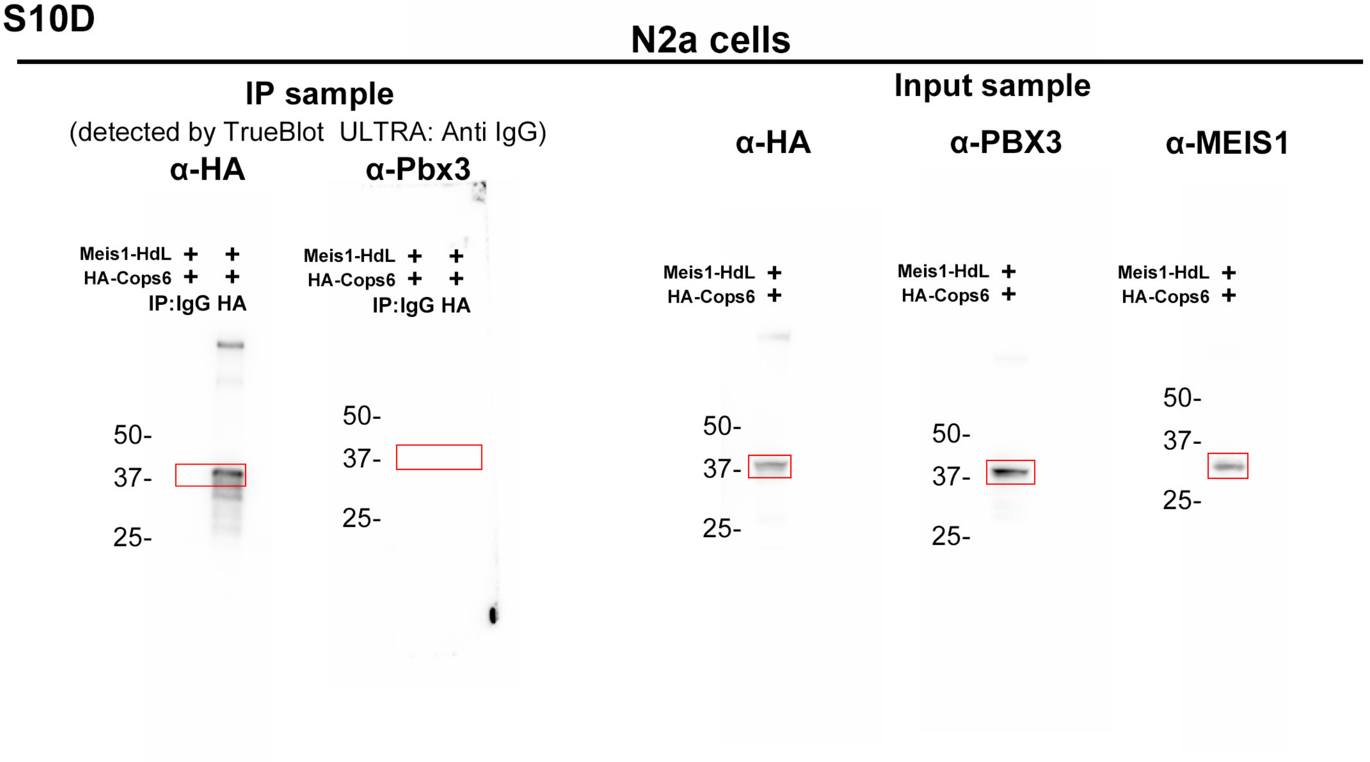

Supplement: S1 Raw Images — This file contains the uncropped blot and gel images underlying the western blot and gel panels presented in the main and supporting figures. Lanes or membrane regions not included in the final figure panels are marked where applicable. (PDF) [file pbio.3003897.s017.pdf]
