# Supplementary material for: Meis1 isoform diversity orchestrates neural progenitor differentiation by regulating ATOH1 degradation at distinct subcellular compartments
Source: PLoS Biol. 2026 Jul 13;24(7):e3003897. doi: 10.1371/journal.pbio.3003897 (PMC13379096; doi:10.1371/journal.pbio.3003897)
Supplement: Attachment — Submitted filename: Point-by-Point Response to Reviewers.docx [file pbio.3003897.s018.docx]

**Overview of the Revised Manuscript**

We wish to express our sincere gratitude to the editors and reviewers for their insightful and constructive comments on our previous manuscript. We apologize for the delay in resubmitting this revised manuscript. The time was necessary because we took the reviewers’ concerns—particularly regarding the existence and physiological relevance of the MEIS1 homeodomain-less (HdL) isoform—very seriously.

To fundamentally address these concerns, we moved beyond standard methods and implemented long-read cDNA sequencing (Nanopore sequencing) and generated a custom isoform-specific antibody. These extensive new analyses not only confirmed the existence of MEIS1-HdL but also enabled us to refine the manuscript’s concept from a study of protein degradation to a broader narrative about isoform diversity orchestrating neural progenitor differentiation, as exemplified by the *Meis1* gene.

We believe these major revisions have significantly strengthened the manuscript and fully addressed the points raised. Our point-by-point responses are listed below.

**Point-by-Point Response to Reviewers**

**Response to Reviewer #1**

We sincerely thank the reviewer for their thorough evaluation of our manuscript and for providing detailed, constructive feedback. Your comments, particularly regarding the validation of the HdL isoform and the consistency of our data, have guided us to perform critical new experiments that have greatly strengthened our conclusions. We have addressed each of your points below.

**Comment 1**

Fig1B+1C and Fig1E are not consistent. The former states that Meis1-HdL is not in GCP, but the latter shows it is enriched in one of the two GCP populations. My guess is the experiments were performed at two different stages. The authors mentioned Fig1B+1C were done in P6 (does it mean postnatal day 6? If so, it helps to define it here, because later in manuscript, other experiments were done at P7, P8 and P11 etc). Has GC differentiation not yet started at this stage? If so, it helps to clearly state it. In Fig1E, there are already differentiated GC, so probably the experiment was done at a later stage? This should be clearly stated in text. Also FACS details should be added to Methods. I also feel Fig1B and 1C may not be necessary, as it only adds confusion. It can probably be removed without affecting the manuscript. In addition, the authors should give a brief introduction to ATOH1+ GCP and ATOH- GCP cells, either in introduction, or here when they first mention them.

**Response:** We appreciate your insightful comments regarding the inconsistencies in the previous Figures 1B/1C and 1E. We agree that the previous PCR and immunoblot data were confusing. Following your suggestion, we have removed those panels and replaced them with comprehensive new analyses using long-read sequencing and specific antibodies to definitively demonstrate the expression of MEIS1 isoforms.

**1. Identification of Meis1-HdL by Nanopore sequencing (New Figure 1):** To unambiguously identify the isoform landscape in GCPs, we performed long-read cDNA sequencing (Nanopore sequencing) on purified GCPs. This analysis successfully identified the Meis1-HdL transcript, which lacks the homeodomain coding sequence. We have presented these results in the New Figure 1.

**2. Detection of endogenous MEIS1-HdL protein (New Figure 2A):** To rigorously verify protein expression, we screened multiple antibodies and generated a custom polyclonal antibody specifically targeting the unique C-terminus of MEIS1-HdL. Using this specific antibody alongside a pan-MEIS1 antibody, we confirmed the robust expression of endogenous MEIS1-HdL protein in GCP lysates (New Figure 2A). The validation of this specific antibody is provided in New Figure S2A. These data clearly resolve the ambiguity of the previous blots.

**3. Clarification of FACS sorting (New Figure 2E):** Regarding the FACS analysis, we have added a schematic diagram to New Figure 2E to visually explain our sorting strategy. We have also updated the Figure Legend and Methods section to explicitly define the isolated populations: ATOH1-expressing GCPs (GFP+RFP+), ATOH1-nonexpressing GCPs (GFP-RFP+), and postmitotic GCs (GFP-RFP-).

**4. Introduction of GCP subpopulations:** Regarding your request for a brief introduction to ATOH1+ and ATOH1- GCPs, we have added a description of the "two-step amplification model" in the Results section (Lines 227) to clearly define these populations when they are first mentioned. Specifically, we stated: *"Our previous work has shown a two-step amplification model for GCP differentiation, where highly proliferative, ATOH1-expressing GCPs give rise to an intermediate, transit-amplifying population that is ATOH1-negative and NEUROD1-positive, before finally exiting the cell cycle to become GCs."* This clarification provides the necessary context for interpreting the differential expression profiles of MEIS1 isoforms.

We believe these revisions provide clear and consistent evidence for the expression of MEIS1 isoforms in the developing cerebellum.

**Comment 2**

Fig 1F-H. The author should confirm the subcellular localization in vivo, probably by electroporating the same GFP tagged two Meis1 isoforms. Different cellular environment can affect protein subcellular localization. Also in Fig 1H, the short Meis1 isoform seems to be uniform in the cells, and not really being excluded from the nucleus. Actually the blot in Fig 1F shows a faint band in the nuclear fraction. The authors should probably modify the text in line 108-115 to make it more precise.

**Response:** We appreciate this important suggestion. We agree that verifying subcellular localization *in vivo* is crucial.

**1. *In vivo* confirmation by electroporation (New Figure 2C, D):** As suggested, we electroporated expression vectors encoding MEIS1-FL-EGFP and MEIS1-HdL-EGFP into GCPs of the P8 cerebellum. As shown in New Figure 2C, MEIS1-FL-EGFP signal was strongly localized to the nucleus, whereas MEIS1-HdL-EGFP signal was robustly observed in the cytoplasm. Quantification of the signal intensity confirms this differential localization (New Figure 2D).

**2. Confirmation of endogenous protein localization (New Figure S2B):** To further validate this finding under physiological conditions, we performed subcellular fractionation of lysates from P7 GCPs and immunoblotted for endogenous MEIS1. Consistent with the electroporation results, endogenous MEIS1-FL was detected in the nuclear fraction, while endogenous MEIS1-HdL was enriched in the cytoplasmic fraction (New Figure S2B).

**3. Refinement of the text:** Regarding your observation about the faint bands (suggesting the separation is not absolute), we agree. Particularly for MEIS1-FL, a fraction of the protein can be detected in the cytoplasm. Accordingly, we have modified the text in the Results section to describe the localization more precisely (Lines 216): *"MEIS1-FL was largely nuclear but also present in the cytoplasm, whereas MEIS1-HdL was predominantly cytoplasmic."*

**Comment 3**

line 178-180. Based on the authors' results, this is true in the N2a cells. But related to point 2 above, this may not be true in vivo, unless they authors show that the Meis1 short isoform is not nuclear in vivo.

**Response:** We fully agree with this point. As detailed in our response to Comment 2, we have addressed this concern by performing subcellular fractionation of lysates from P7 GCPs (New Figure S2B). This analysis confirmed that endogenous MEIS1-HdL is predominantly localized in the cytoplasmic fraction and is largely excluded from the nuclear fraction in GCPs, validating that our observations in N2a cells reflect the physiological state *in vivo*.

**Comment 4**

Fig5E-H. This is one place where in vivo confirmation is lacking. The authors should electroporate control or sh-Cul3, and examine for ATOH1 expression, as well as GCP and GC cell counts.

**Response:** We strictly followed this suggestion and performed *in vivo* electroporation of sh-*Cul3* into P8 cerebella to examine ATOH1 expression and GCP/GC counts. However, contrary to the *in vitro* results, we did not observe a significant increase in ATOH1-positive cells or a blockade of differentiation (please see Figure R1, presented at the end of this response).

We believe this discrepancy is due to the kinetics of shRNA-mediated knockdown relative to the rapid differentiation speed of GCPs at this developmental stage.

1. **Time lag of shRNA:** Unlike overexpression vectors (which produce protein rapidly), shRNA vectors require time for transcription, processing, and the turnover of the pre-existing target protein. It is generally established that significant protein reduction by shRNA often takes 48–72 hours or more.
2. **Rapid developmental window:** P8 GCPs are programmed to differentiate rapidly. During the lag period of CUL3 depletion, many GCPs likely proceed to differentiate driven by the physiological developmental program before CUL3 levels drop below the threshold required to inhibit ATOH1 degradation.

Indeed, we encountered the same issue with sh-*Meis1-FL* (knockdown of the degradation-promoter), which also failed to show a "maintenance of undifferentiated state" phenotype in this specific *in vivo* window, despite MEIS1-FL’s clear role in promoting degradation. In contrast, overexpression of MEIS1-HdL provides an immediate supply of the stabilizer protein, allowing us to detect the phenotype (undifferentiated state) clearly.

Given these technical limitations of *in vivo* knockdown for observing "differentiation arrest" in this rapid system, we believe our extensive *in vitro* biochemical analyses (Figure 5 & 6)—which consistently show CUL3 binds and ubiquitinates ATOH1 and that MEIS1 blocks this—provide strong and sufficient evidence for the mechanism.

**Figure R1. Evaluation of *in vivo* knockdown efficiency and phenotypic outcomes (Data for Reviewer #1).**

**A.** Validation of *sh-Meis1-FL* specificity. Immunoblotting of N2a cells co-transfected with MEIS1-FL or MEIS1-HdL and the indicated shRNAs confirmed that *sh-Meis1-FL(sh-Meis1-exon8)* specifically targets the FL isoform.

**B.** Representative immunofluorescence images of P11 cerebella electroporated at P8 with *sh-Cul3* or *sh-Meis1-FL*. Sections were analyzed using two staining combinations: **(1)** Triple staining for ATOH1 (magenta), Ki67 (cyan), and GFP (yellow); and **(2)** Double staining for p27 (magenta) and GFP (green).

**C.** Quantification of differentiation markers (ATOH1, Ki67, p27) in GFP+ electroporated cells. Despite the validated shRNA efficacy *in vitro*, neither *sh-Cul3* nor *sh-Meis1-FL* elicited significant changes in differentiation or ATOH1 levels in this rapid developmental window (P8–P11), likely due to the slow kinetics of shRNA-mediated protein depletion relative to cell differentiation speed. (n = 4 mice per group; N.S., not significant).

**Comment 5**

Lines 235-237. This is another place where in vivo confirmation is not performed. Can the authors use electroporation to overexpress or knock down ERK1, and examine the same readouts as above? Since the authors put these results in supplementary figures, the in vivo confirmation here, although desired, may be optional.

**Response:** We have carefully considered this suggestion regarding the *in vivo* validation of ERK1. In the process of extensively revising the manuscript, we decided to remove the section regarding the upstream kinase (ERK) identification. With the incorporation of the new Long-read sequencing data and the discovery of the broad "isoform diversity" landscape, we felt that delving into the upstream kinase signaling (ERK) distracted from the manuscript's core message: the functional antagonism between MEIS1-FL and MEIS1-HdL isoforms.

Therefore, we have streamlined the story to focus on the MEIS1-HdL–CSN–CUL3 axis, which is the direct machinery responsible for the isoform-dependent regulation of ATOH1 stability. We believe this focused narrative significantly improves the clarity and impact of the study.

**Comment 6**

Figure 6D. I believe the authors' state that both Meis1 isoforms are likely interacting with COPS5, but the logic here is not very tight. This experiment was done one day later than in Fig1B+C, so in theory the expression of Meis1 isoforms may not be the same as in Fig1B+C. It helps to determine the expression of the two Meis1 isoforms at P7, simply blot the input with Meis1 antibody should serve this purpose. In addition, the authors should use, whenever feasible, in vivo materials to repeat several key in vitro biochemical results, such as the interaction between Cul3 and ATOH1, and the interaction between different COPS and Cul3? The manuscript would be much stronger if these in vivo experiments show the same conclusions as the in vitro results.

**Response:** We appreciate this precise suggestion regarding the developmental timing. In the revised manuscript, we standardized the developmental stage for biochemical analyses to P7. To address your concern, we performed Western blotting on P7 GCP lysates (the same stage used for the IP experiment) as shown in New Figure 2A. Using both the pan-MEIS1 antibody and our new HdL-specific antibody, we clearly detected both MEIS1-FL and MEIS1-HdL bands at P7. This confirms that both isoforms are expressed in P7 GCPs and are available to interact with the CSN complex.

Furthermore, regarding your suggestion to verify the CUL3-ATOH1 interaction using *in vivo* materials:

**1. Confirmation of MEIS1-CSN interaction (Figure 6D):** First, we prioritized validating the most novel part of our finding. We successfully co-immunoprecipitated endogenous MEIS1 with CSN components (COPS4 and COPS5) using P7 GCP lysates (Figure 6D). This confirms that the MEIS1-CSN complex indeed exists in the developing cerebellum.

**2. Difficulty in detecting CUL3-ATOH1 interaction:** Regarding the endogenous CUL3-ATOH1 interaction, detecting robust signals proved technically challenging. We attribute this to two reasons:

- **Transient nature:** E3 ligase-substrate interactions are inherently transient, and endogenous ATOH1 is rapidly degraded.
- **Theoretical consistency:** Crucially, our study proposes that MEIS1-HdL functions to inhibit the binding of CUL3 to ATOH1. Since we have demonstrated that MEIS1-HdL is expressed in P7 GCPs (as shown in Figure 2A), it is theoretically expected that the interaction between CUL3 and ATOH1 is strongly suppressed in these cells. Therefore, the difficulty in detecting the CUL3-ATOH1 complex *in vivo* is actually consistent with our conclusion that MEIS1-HdL acts as an inhibitor of this interaction.

Instead, we provided functional genetic evidence *in vivo* (Figure 7), showing that *Cops6* knockdown completely abolishes the effect of MEIS1-HdL, which we believe serves as strong support for the pathway.

**Comment 7 (originally numbered 6):**

Fig 6H, DNA quantity used in transfection is very weak evidence for the claim that the short Meis1 isoform has a stronger effect in blocking ATOH1 degradation. More evidence is needed. Isoform-specific regulation of ATOH1 degradation is a major conclusion the authors want to draw, and this evidence alone is far from sufficient. Again I think some in vivo electroporation experiments will help. Can the authors overexpress or knockdown the two isoforms in vivo and examine ATOH1 protein levels? FigS4 has some knockdown experiments (will be better if there is full-length specific knockdown), but overexpression would be needed here to confirm the in vivo observation.

**Response:** We appreciate this comment. We agree that DNA titration in N2a cells alone is insufficient. Therefore, we focused on the in vivo functional validation.

**1. In vivo Overexpression (Figure 3):** The reviewer asked to "overexpress the two isoforms in vivo and examine ATOH1 protein levels." We have indeed performed this analysis in the original manuscript (Figure 3).

- **MEIS1-FL overexpression:** Significantly reduced the proportion of ATOH1-positive cells and promoted differentiation (Figure 3B, F). This mirrors our previous finding that *Meis1* conditional knockout mice (which lack the homeodomain-containing FL isoform) exhibit delayed differentiation (Owa et al., 2018).
- **MEIS1-HdL overexpression:** Conversely, significantly increased the proportion of ATOH1-positive cells and maintained the undifferentiated state (Figure 3C, F). These in vivo results clearly demonstrate that the two isoforms exert opposite effects on ATOH1 stability (maintenance of ATOH1+ population) and GCP fate.

**2. In vivo Knockdown of MEIS1-FL (Figure R1):** Regarding the knockdown of MEIS1-FL, we performed the experiment using a specific shRNA (*sh-Meis1-FL*). However, unlike *sh-Meis1-HdL*, *sh-Meis1-FL* did not show a significant phenotype regarding ATOH1 levels or differentiation (please see Figure R1, which also contains the *sh-Meis1-FL* data). We interpret this result based on the developmental context and kinetics:

- **Loss of "Brake" (*sh-Meis1-HdL*):** Removing the stabilizer (HdL) in rapidly differentiating P8 GCPs accelerates ATOH1 loss (Figure 4).
- **Loss of "Accelerator" (*sh-Meis1-FL*):** Removing the accelerator (FL) has little effect because the cells are already driven toward differentiation. Furthermore, the time lag of shRNA efficacy makes it difficult to detect a "slowed differentiation" phenotype in this rapid window. Importantly, the physiological role of MEIS1-FL in promoting differentiation has already been established in our previous study using genetic *Meis1* conditional knockout mice (Owa et al., 2018), which supports our model. Therefore, the clear opposite overexpression phenotypes (Figure 3) and the *sh-Meis1-HdL* phenotype (Figure 4) collectively support our conclusion.

**Comment 8 (originally numbered 7):**

Fig 6J. ATOH1 is a transcription factor, why is there so much signal in the cytoplasm? This needs to be explained.

**Response:** We appreciate this careful observation. While ATOH1 is a nuclear transcription factor, we detect signals in the cytoplasm in this experiment (Figure 6J) for the following reasons:

1. **Overexpression:** High-level overexpression in N2a cells can saturate the nuclear import machinery, resulting in cytoplasmic accumulation.
2. **Site of Regulation (Synthesis and Degradation):** Biologically, ATOH1 is synthesized in the cytoplasm and must traverse it to enter the nucleus. Crucially, we identified that CUL3 (the E3 ligase for ATOH1) is predominantly cytoplasmic (Figure 6J). This implies that the cytoplasmic pool of ATOH1—likely immediately after synthesis or before nuclear import—is the specific target for CUL3-mediated ubiquitination. Therefore, the presence of ATOH1 in the cytoplasm is not merely an artifact but represents the subcellular compartment where the critical regulation (ubiquitination) occurs.
3. **Long exposure:** To visualize the poly-ubiquitinated smear (high-molecular-weight species), we used long-exposure imaging, which naturally enhanced the signal of the unmodified cytoplasmic monomer.

We have added a brief explanation regarding this cytoplasmic signal in the Figure Legend of Figure 6J.

**Comment 9 (originally numbered 8):**

Fig 7A-E, the authors should overexpress and knockdown cops6 without manipulating Meis1, and examine the effects on ATOH1 expression level, as well as GCP and GC cell counts.

**Response:** We appreciate this suggestion to clarify the independent role of COPS6 in GCPs.

**1. *In vivo* Knockdown of *Cops6* (Figure 7C-E, Figure S10):** We performed the requested knockdown experiment by electroporating sh-*Cops6* alone into P8 cerebella.

- **Results:** As shown in Figure S10A-B (representative images) and Figure 7C-E (quantification), *Cops6* knockdown significantly reduced the proportion of proliferative GCPs (Ki67+, ATOH1+) and increased differentiated GCs (p27+).
- **Conclusion:** This phenotype mirrors the loss of MEIS1-HdL (Figure 4), confirming that the physiological function of the CSN complex is essential for maintaining the undifferentiated state of GCPs, consistent with our model.

**2. Regarding Overexpression:** Regarding the overexpression of COPS6, we reasoned that since the COP9 Signalosome (CSN) is a multi-subunit complex consisting of eight components (COPS1–8), overexpressing a single subunit (COPS6) would unlikely result in increased holocomplex activity due to the stoichiometric limitation of other subunits. Therefore, we focused on the loss-of-function (knockdown) approach, which clearly demonstrated the necessity of COPS6.

**Response to Reviewer #1, Minor Points**

**[Minor Points & Text Clarifications]**

**Point 1:** Line 49-51 and 86-87. It is helpful to more clearly state where different cells are found. Which cells are in the oEGL? Only GCP, or also some differentiated GC? How about the iEGL? Are there only GC in the iEGL? Or the iEGL also has some GCP?

**Response:** We have revised the Introduction and Results sections to clearly define the cellular composition of the EGL layers. Specifically, we stated that the oEGL contains highly proliferative ATOH1-expressing GCPs, while the iEGL contains ATOH1-nonexpressing (NEUROD1+) GCPs and postmitotic GCs that are beginning to migrate (Lines 69-75).

**Point 2:** line 68-71. Meis1 homeodomain conditional knockout was specific to the already differentiated GC cells, how can this delay GCP to GC differentiation? The authors need to word this part more clearly.

**Response:** We apologize for the confusing phrasing in the previous manuscript. We have rewritten this section in the Introduction to accurately describe our previous findings (Owa et al., 2018). We clarified that *Meis1-FL* functions to promote the transition from progenitors to differentiated cells, and its loss delays this process (Lines 87-90).

**Point 3:** Line 166, briefly introduce the function of cycloheximide

**Response:** We have added the phrase "(CHX, a protein synthesis inhibitor)" to the text to clarify its function for general readers (Lines 334).

**Point 4:** Line 262, "Immunoblotting followed by LC-MS/MS did not reveal the presence of COPS5". I'm confused, is there something wrong here? Western blot clearly shows COPS5 signal, similar or stronger than COPS2.

**Response:** We apologize for the confusing phrasing in the previous manuscript. What we intended to convey was that while COPS5 was not initially detected in the comprehensive LC-MS/MS screening (likely due to low abundance or peptide ionization efficiency), the subsequent targeted Western blotting clearly confirmed its robust interaction.

In the revised manuscript, we have rewritten this section to avoid confusion. We first list the CSN components identified by MS (COPS2, 3, 4, 6, 7a, and 8) to establish the interaction with the complex, and then present the Western blot data (Figure 6B) which definitively validates the interaction with COPS5 (Lines 424-435).

**Point 5:** Line 308, change "intracellular regions" to cytoplasm and nucleus.

**Response:** We have corrected this phrase to "both the cytoplasm and nucleus" as suggested (Lines 484).

**Other 1:** FigS5E, loading controls have different intensities, which makes the authors' claims weaker.

**Response:** We have replaced these blots with new, high-quality images in the revised manuscript (New Figure S8). We ensured equal loading and provided proper loading controls (GAPDH) to support our claims robustly.

**Other 2:** Fig S6A, the right most lane seems to show increased phosphorylation, maybe indirect effect? Probably should explain at least in legend.

**Response:** As mentioned in our Response to Comment 5, we have removed the section and figures regarding the upstream kinase (ERK) signaling (including the previous Fig S6A) to streamline the manuscript and focus on the core MEIS1-HdL mechanism.

**Response to Reviewer #2**

We sincerely thank the reviewer for the positive assessment of our study and for raising critical points regarding the isoform-specific mechanisms. We have addressed the concerns by adding in vivo localization data and by clarifying the mechanism of functional divergence between the two isoforms in the revised manuscript.

**Comment 1**

One key point of this study of the differential localization of MEIS1-HdL and MEIS1-FL, which influences the differential stabilizing action on ATHO1 protein and links to the proposed mechanism of protein degradation inhibition. While evidence for the differential localization is provided in Fig1 F-H in N2A cells, this is not shown for the granule cell lineage. However, this evidence is important to support the proposed model.

**Response:** We fully agree with this point. Demonstrating differential localization in the GCP lineage is essential for our model. In the revised manuscript, we provided two lines of *in vivo* evidence:

1. **In vivo electroporation:** We electroporated EGFP-tagged MEIS1-FL and MEIS1-HdL into P8 cerebella. As shown in New Figure 2C and D, MEIS1-FL is predominantly nuclear, whereas MEIS1-HdL is predominantly cytoplasmic in GCPs.
2. **Endogenous fractionation:** We performed subcellular fractionation of P7 GCP lysates, confirming that endogenous MEIS1-HdL is enriched in the cytoplasmic fraction (New Figure S2B).

**Comment 2**

The authors show that both 'MEIS1-HdL and full-length MEIS1 form a trimeric complex with CUL3 and COP9 signalosome that inhibits ATOH1 ubiquitination and degradation'. How is this finding compatible with the observation that '(line 125) MEIS1-FL overexpression decreased GCP counts and increased GC counts, whereas the opposite trend was observed when MEIS1-HdL was overexpressed (Fig.2B-E, Fig.S2B, C)?

**Response:** This apparent paradox is resolved by the differential subcellular localization validated in Comment 1.

- **Physiological State (In vivo):** MEIS1-FL is confined to the nucleus (Figure 2C). Therefore, it acts primarily as a transcription factor (promoting *Pax6* and differentiation) and is physically separated from the cytoplasmic CUL3-ATOH1 degradation machinery. Thus, it cannot stabilize ATOH1 *in vivo*, and its pro-differentiation transcriptional effect dominates.
- **Overexpression State (In vitro):** In our biochemical assays using N2a cells (Figure 5 & 6), high-level overexpression causes some MEIS1-FL to leak into the cytoplasm (as seen in Figure S2C). This leaked FL can interact with cytoplasmic CUL3/CSN, artificially stabilizing ATOH1.

The opposite phenotypes observed *in vivo* (Figure 3) confirm that under physiological conditions, the nuclear (FL) vs. cytoplasmic (HdL) separation dictates their distinct functions. MEIS1-HdL is the dedicated cytoplasmic isoform that stabilizes ATOH1.

**Comment 3**

The authors should clarify this point and more deeply discuss potential divergent actions of the isoforms in relation to their amount, localization, mode of action etc.

**Response:** We appreciate this suggestion. We have significantly revised the Discussion section (Line 558) to explicitly address the mechanisms underlying the divergent actions of the isoforms.

Specifically, we clarify that while MEIS1-FL possesses the biochemical capability to bind CSN/CUL3 (as observed in overexpression assays where cytoplasmic leakage occurs), its physiological function is distinct due to strict spatial confinement.

We have added the following text to the Discussion to precisely describe this mechanism: *"In line with this notion, although MEIS1-FL shows partial cytoplasmic localization when overexpressed in vitro, it is largely absent from the cytoplasmic fraction of GCPs in vivo. This spatial separation likely largely restricts MEIS1-FL from accessing the cytoplasmic CUL3-ATOH1 machinery under physiological conditions, indicating that MEIS1-HdL serves as the major cytoplasmic isoform responsible for ATOH1 stabilization."*

This revision clarifies that the subcellular localization (nuclear vs. cytoplasmic) serves as the primary determinant for the functional divergence of these isoforms *in vivo*.

**Response to Reviewer #2, Minor Points**

**[Minor Points & Text Clarifications]**

**Point 1:** The meaning of the following sentence remains unclear to this reviewer: line 348 If this is the case, the localization of Cullin family proteins to the cell nuclei, such as CUL1 and CUL4B, may be inactivated by MEIS1-FL, which localizes preferentially to the nuclei.

**Response:** We agree that the original sentence was speculative and unclear. In the revised Discussion, we have removed the specific hypothesis regarding the nuclear inactivation of CUL1/CUL4B by MEIS1-FL. Instead, we now limit our comment to stating that CUL1 and CUL4 were identified as binding partners in our proteomic analysis (Line 590), suggesting a potential broad role for MEIS1 in protein stability, without drawing premature conclusions about their isoform-specific regulation based on localization.

**Point 2:** Lines 373-379 The authors seem to introduce a parallelism between MYC-nick and MEIS1-Hd. However, this appears poorly linked to the presented findings and proposed mechanism.

**Response:** We accept this criticism. We have removed the paragraph regarding MYC-nick from the revised Discussion to ensure the text remains tightly linked to our presented findings.

**Point 3:** Potential regulatory mechanisms of Meis1-HdL expression which appear to vary within the GC lineage at least at the mRNA level should be discussed.

**Response:** We have addressed this by linking our FACS data to the insights gained from our long-read sequencing.

1. **Stage-Specific Switching:** Our FACS-qPCR data (Figure 2F) confirms that *Meis1-HdL* expression is strictly confined to the proliferative ATOH1+ stage, while *Meis1-FL* expression persists in later stages.
2. **Regulatory Machinery (Fig 1D):** Importantly, our Nanopore sequencing revealed that genes with high isoform diversity in GCPs are significantly enriched for "mRNA splicing" and "RNA processing" functions (Figure 1D). This suggests that the splicing machinery itself is highly complex and active in GCPs.
3. **Discussion:** In the revised Discussion, we propose that this sophisticated regulation of splicing factors likely orchestrates the precise stage-specific generation of *Meis1* isoforms (HdL vs. FL), ensuring the correct timing of GCP differentiation.

**Point 4:** Number of analysed samples (cerebella/slices/blots etc) need to be indicated for quantified measurements.

**Response:** We have ensured that the number of samples (n), representing independent biological replicates (e.g., number of mice or independent cultures), is clearly indicated in each Figure Legend.

**Point 5:** Fluorescence intensities in Figure S4 cerebella - this seems to be measured by cell. The authors should add more methodological information to grant reproducibility. Such measurement (fluorescence intensity in nuclei) could vary if nuclear areas are affected by the manipulations. Can the authors exclude this?

**Response:** We have addressed both the methodological detail and the potential artifact concern.

**1. Methodological Information:** We have added a detailed description of the quantification method to the Materials and Methods section. Specifically, we used ImageJ to define the nuclear region of interest (ROI) based on DAPI staining. Crucially, to strictly exclude artifacts caused by potential changes in nuclear area or chromatin condensation, we normalized the fluorescence intensity of the target protein (ATOH1, Ki67) to the DAPI intensity of the same nucleus.

**2. Exclusion of Nuclear Size Artifacts:** By normalizing to DAPI intensity, any fluctuation in signal density caused by nuclear volume changes is mathematically corrected. Furthermore, as an internal biological control shown in Figure S6, the DAPI-normalized ATOH1 signal significantly decreased in knockdown cells, whereas the DAPI-normalized Ki67 signal remained unchanged. This confirms that the observed reduction is specific to the ATOH1 protein and is not an artifact of nuclear morphology.

**Point 6:** Full length blots need to be included in all instances. Also, protein molecular weights need to be shown.

**Response:** We have complied with this requirement. We have provided uncropped full-length blot images in the source data files. In the main and supplementary figures, we have indicated the molecular weights (kDa) on the blots.

**Response to Reviewer #3**

We sincerely thank the reviewer for the thorough assessment of our manuscript and for recognizing the potential impact of our proposed mechanism. We deeply appreciate your critical comments regarding the existence of the homeodomain-less (HdL) MEIS1 isoform. We took your skepticism very seriously. To address your concerns "once and for all," we implemented long-read Nanopore sequencing and generated a custom isoform-specific antibody, providing definitive evidence that the MEIS1-HdL isoform is indeed expressed and functional in cerebellar granule cell progenitors (GCPs).

**Comment 1**

Much of the present study deals with MEIS1-HdL, a C-terminally truncated form of MEIS1 that lacks the DNA-binding homeodomain (HD). However, evidence for this is weak. First, it is still a matter of debate in the field whether HD-less MEIS isoforms actually exist. Neither NCBI nor ENSEMBLE list any such HD-less transcript variants as protein-coding isoforms. Until a few years ago, a C-terminally truncated isoform termed Meis1e was listed in NCBI, but this seems to have been removed. At present, all protein coding murine MEIS1 transcripts in NCBI or ENSEMBLE contain the HD, and all transcripts that lack the HD are considered to be subject to nonsense mediated decay.

Evidence for HD-lacking MEIS-protein variants in the scientific literature is also inconclusive. Geerts et al., Cancer Letters 2003 (cited by the authors) discuss a HD-less MEIS1 variant (termed MEIS1e) but provide no experimental evidence whatsoever for its existance. Instead, they cite Yang et al., JCB 2000, who had cloned a C-terminally truncated variant of MEIS2 (termed MEIS2d) from a commercial cDNA library in the course of a 2-hybrid screen. Because of these discrepancies, additional efforts are needed to clarify once and for all whether a HD-less MEIS1 protein exists. One approach the authors may want to take is to interrogate the many RNA-seq or proteomic data that exist in the public domain for the presence of a HD-less MEIS1. For more direct tests, Crist et al., PLoSone 2011 reported on an antibody specifically targeting the short C-terminus of HD-less MEIS1. The authors should try to access this antibody and test it on their samples. Not to be misunderstood, I am

not suggesting that a HD-less MEIS1 transcript variant does not exist. I just think that the evidence for it is rather weak at the moment. The theory proposed by the authors that an HD-less form of MEIS1 controls the stability of ATOH1, as beautiful as it may be, still lacks the experimental basis. Or in other words, although the authors provide good experimental arguments for a novel, HD-independent function of MEIS1, they do not prove beyond doubt that the observed effects indeed stem from a C-terminally truncated protein. Please see also my argument 10 below.

**Response:** We sincerely appreciate this critical comment. We fully agree that establishing the physical existence of the HD-less MEIS1 isoform is the fundamental prerequisite for our study. We acknowledge that this isoform is not currently listed in standard databases like RefSeq, and that previous literature has lacked definitive molecular proof.

To settle this debate "once and for all," as suggested, we performed decisive experiments to prove the existence of MEIS1-HdL at both the transcript and protein levels:

**1. Definitive Transcript Identification (New Figure 1 & RT-PCR cloning):** We reasoned that the ambiguity in public databases stems from the limitations of short-read RNA sequencing. To overcome this, we utilized long-read Nanopore sequencing on purified GCPs.

- **Nanopore Result:** This analysis successfully and unambiguously identified the full-length transcript of *Meis1-HdL*, which lacks the homeodomain coding sequence due to alternative splicing (New Figure 1).
- **Validation by Cloning:** To further verify this result, we performed RT-PCR cloning from GCP total RNA. We successfully isolated and cloned the cDNA corresponding to *Meis1-HdL*. Sanger sequencing of this clone perfectly matched the sequence identified by Nanopore sequencing, confirming that this transcript physically exists and can be isolated from GCPs, independent of database annotations.

**2. Definitive Protein Identification via Custom Specific Antibody (New Figure 2):** To address the concern about Nonsense-Mediated Decay (NMD) and prove that this transcript is translated into a stable protein, we focused on protein-level detection. Instead of relying on existing antibodies that may be unavailable or of uncertain specificity, we generated a custom polyclonal antibody specifically targeting the unique C-terminal peptide of MEIS1-HdL (which is distinct from MEIS1-FL due to the frameshift).

- **Result:** Using this specific antibody, we detected a robust endogenous band of the predicted size in GCP lysates (New Figure 2A).
- **Validation:** We further validated the specificity of this antibody using overexpression samples (Figure S2A).

**Conclusion:** These data provide incontrovertible evidence that MEIS1-HdL is not an annotation error or an NMD target, but is expressed as a stable, functional protein in developing GCPs. We believe these results resolve the long-standing debate in the field and provide the solid experimental basis requested by the reviewer.

**Comment 2**

Along the same line: the PCR test shown in Fig.1 only confirms the presence of MEIS1-transcripts that lack exon8, not the existence of transcripts that correspond to the cDNA that the authors cloned and overexpressed as MEIS1-HdL.

**Response:** We completely agree with the reviewer that the previous RT-PCR data was limited to detecting local exon skipping and could not verify the full-length transcript structure.

However, this concern has been fully resolved by the Nanopore long-read sequencing presented in New Figure 1. Unlike standard PCR or short-read sequencing, Nanopore sequencing captures full-length transcripts. As detailed in our Response to Comment 1, our Nanopore analysis definitively identified the exact full-length transcript corresponding to the MEIS1-HdL cDNA we cloned and used for overexpression. This confirms that the HdL isoform we studied is not a PCR artifact or a theoretical construct, but a genuine transcript expressed in GCPs.

**Comment 3**

An unfortunate weakness of the present study is that it relies almost exclusively on overexpression experiments. Nearly all biochemical experiments involve ectopic expression of GST- or HA-tagged proteins in N2A cells. Likewise, the in vivo experiments shown in Fig.2 and Fig.7 also rely on electroporation of expression plasmids. To reduce gene function, shRNA knockdown experiments were done, but these also rely on massive overexpression of the respective pasmids. Such approaches give a good first indication of whether a particular protein-protein interaction is possible in principle but are highly unphysiological. It would be desirable if overexpression approach, at least in the in vitro setting, was backed up by genetic evidence, e.g. by CRISPR/Cas-mediated removal of exon 8 from the MEIS1 gene to mimic MEIS1-HdL.

**Response:** We appreciate this constructive suggestion regarding the limitations of overexpression and the need for genetic evidence.

**1. Genetic evidence from *Meis1* conditional knockout (Owa et al., 2018):** Regarding your suggestion to "remove exon 8," we have, in fact, already performed this exact genetic experiment in our previous study (Owa et al., 2018). We utilized a conditional knockout mouse line targeting *Meis1* Exon 8 (which encodes the homeodomain). In these mice, the loss of the homeodomain (functionally eliminating MEIS1-FL) resulted in the accumulation of ectopic ATOH1-positive GCPs in the inner EGL. At the time (2018), we interpreted this primarily as a transcriptional defect. However, in light of our current findings, this genetic phenotype is consistent with our proposed model: the loss of MEIS1-FL (the degradation promoter) shifts the balance, leading to ATOH1 stabilization and the maintenance of the undifferentiated state. Thus, the "genetic evidence" requested by the reviewer already exists and strongly supports our conclusion that the MEIS1 homeodomain is critical for terminating the ATOH1+ progenitor state.

**2. Physiological interaction (Endogenous Co-IP, New Figure 6D):** To further address the concern about "unphysiological" overexpression artifacts in the current study, we have added Endogenous Co-Immunoprecipitation (Co-IP) data using P7 GCP lysates (New Figure 6D). Instead of using tagged overexpression vectors, we immunoprecipitated endogenous MEIS1 (using an antibody that recognizes both isoforms) and successfully detected its interaction with endogenous CSN components (COPS4 and COPS5). While this IP pulls down both isoforms, our in vitro data (Figure 6B) confirmed that both FL and HdL possess the structural capacity to bind the CSN. Given that MEIS1-HdL is expressed in these P7 GCP lysates (as shown in New Figure 2A), this result strongly supports that the interaction between MEIS1 (including HdL) and the CSN complex occurs under physiological conditions in the developing cerebellum, and is not an artifact of overexpression.

**Comment 4**

The authors make the argument that expression of a C-terminally truncated MEIS1, which differs from that of MEIS1-FL on cellular and subcellular level, affects the balance between cell proliferation and differentiation in the developing cerebellum. Yet, the only evidence for the presence of such a truncated MEIS1-HdL in granule cell progenitors is given in Fig.1C. The protein band corresponding to MEIS1-HdL in this figure is very weak, less than 5-10% of the protein load of MEIS1-FL. With this in mind, how do the authors justify that they overexpressed seemingly equal amounts of MEIS1-FL and -HdL in their functional studies? Along the same line, the argument that MEIS1-HdL is cytoplasmic is solely based on the subcellular localization of the overexpressed protein (Fig.1F). Since this lacks the basic amino acids at the beginning of the HD and thus the putative nuclear import signal, its cytoplasmic localization is not very surprising.

**Response:** We appreciate this critical analysis. We agree that the immunoblot data in the previous manuscript (using a suboptimal antibody) showed weak signals, raising legitimate concerns about the stoichiometry and physiological relevance of MEIS1-HdL. However, we have fundamentally addressed this concern by screening multiple commercial antibodies and establishing a robust detection system.

1. **Robust Expression Confirmed by Optimized Pan-MEIS1 Antibody (New Figure 2A):** To demonstrate that MEIS1-HdL is a significant protein species, we tested several commercial anti-MEIS1 antibodies (recognizing all isoforms) and identified one with superior sensitivity and specificity. Using this optimized pan-MEIS1 antibody (alongside our custom HdL-specific antibody), we replaced the blot in New Figure 2A. The new data clearly show that MEIS1-HdL is expressed as a robust, distinct band in P7 GCPs, far more substantial than the "5-10% weak band" observed with the previous poor-quality antibody. This confirms that HdL is a genuine, stable isoform expressed at physiologically relevant levels.
2. **Stoichiometry and Justification based on Local Concentration (New Figure S2B):** Regarding the reviewer's point that MEIS1-FL appears more abundant than HdL in whole cell lysates: We agree that in *total* lysates, the FL signal is stronger (reflecting the large nuclear pool). However, we argue that physiological relevance depends on the local concentration in the specific subcellular compartment where the function (ATOH1 degradation) occurs.
   - Cytoplasmic Dominance: We performed subcellular fractionation of P7 GCP lysates and probed them with the pan-MEIS1 antibody. As shown in New Figure S2B, MEIS1-FL is largely sequestered in the nucleus. Crucially, in the cytoplasmic fraction—where CUL3-mediated ubiquitination occurs—MEIS1-HdL is the predominant isoform. Since MEIS1-HdL is the major species available to interact with the degradation machinery in the cytoplasm, treating it as a key functional regulator is physiologically justified.

**Comment 5**

Many of the Western Blots are of poor quality. (1) All blots are heavily cropped. An extreme example can be seen in Fig.S7C, but the problem of overly trimmed images applies essentially to all immunoblots. The blot in Fig.S7C is cropped so tightly that the MEIS1-FL-specific band is barely visible at the top of the image. Suspiciously, a weak band can also be seen in the first lane, where MEIS1-FL should not be expressed. (2) All blots lack information on protein standards / marker lanes. Therefore, the reader cannot judge the quality of the blots and the separation of protein bands. (3) In many panels individual lanes, which are supposedly generated by stripping and re-probing of the same membrane, do not match. See for example Fig.1F, Fig.5E, G, Fig.6D, E, H, Fig.S3A, Fig.S7C.

**Response:** We sincerely apologize for the suboptimal quality and presentation of the immunoblot data in the previous manuscript. We fully accept the reviewer's criticism regarding the cropping, lack of markers, and lane consistency. We have taken the following rigorous steps to address these issues:

1. **Replacement with High-Quality Data:** We have repeated the experiments for figures where image quality was particularly compromised and replaced them with new, high-resolution images.
2. **Uncropped Source Data & Molecular Weights:** To guarantee transparency and allow for independent verification, we have provided uncropped, full-length blot images for all Western blotting data in the Source Data files. We have explicitly indicated the molecular weight markers (kDa) within these files and figures.
3. **Rigorous Alignment and Consistency:** For all figures, we have carefully reviewed the original data and reconstructed the panels to ensure that all lanes (including Input controls) are properly aligned and accurately represent the experimental results.

**Comment 6**

In all figures that show pulldown or IP experiments the input(s) are missing. To give an example: In Fig.5 GST-ATOH1, MEIS1-FL and MEIS1-HdL were transfected into N2A cells but only the input of (endogenous) CUL3 is shown. How do the amounts of the three ectopically expressed GST-ATOH1, MEIS1-FL and MEIS1-HdL compare to the CUL3 protein band? If the misexpressed proteins are significantly more abundant than endogenous CUL3, how can this be physiological?

**Response:** We have addressed both the data presentation and the physiological concern.

**1. Inclusion of Inputs:** We apologize for the omission. We have now included the Input blots for all transfected proteins in every relevant figure to clearly demonstrate the expression levels and loading controls.

**2. Physiological Relevance:** Regarding the reviewer's concern about the physiological relevance of the stoichiometry in overexpression experiments: We agree that overexpression systems do not strictly recapitulate physiological protein ratios. These *in vitro* experiments were primarily intended to dissect the molecular mechanism and binding domains. However, to address the core question—"Does this interaction occur under physiological conditions?"—we performed Endogenous Co-Immunoprecipitation using P7 GCP lysates (New Figure 6D). As discussed in our response to Comment 3, we successfully detected the interaction between endogenous MEIS1 and the CSN complex (COPS4/5) at native expression levels. Since the CSN complex is the canonical regulator of CUL3 ubiquitin ligases, demonstrating the physiological MEIS1-CSN interaction provides strong evidence that MEIS1 engages the endogenous CUL3 machinery in the developing cerebellum, validating the biological relevance of our model.

**Comment 7**

Why was MG132 added to some experiments (e.g. Fig.5A) but not to others (e.g. Fig.5F, Fig.S5B)?

**Response:** We appreciate the reviewer’s attention to detail regarding the experimental conditions. We agree that the experimental protocols should be consistent. To address this, we re-performed the ubiquitination assay for Figure 5A without adding MG132, ensuring the conditions are identical to those in other figures (e.g., Figure 5F). The new results, presented in the revised Figure 5A, consistently confirm that overexpression of both MEIS1-FL and MEIS1-HdL suppresses ATOH1 ubiquitination compared to the control. We have updated the figure and the corresponding text to reflect these consistent findings.

**Comment 8**

Mass spec. experiments, lines 186/187: The authors state that they 'identified several molecules (Fig.5D) that bind ATOH1 only in the absence of MEIS1.' and list 7 proteins that are linked to ubiquitination and protein degradation in Fig.5D. Strictly spoken, except CUL3 these proteins do not fulfil the criterion of 'binding ATOH1 only in the absence of MEIS1'. In addition, how were the proteins selected? Were they among the top % of proteins that were most strongly enriched in the absence of MEIS1 or did the authors simply search the >1500 proteins they had identified for candidates with a known link to protein ubiquitination or -degradation?

**Response:** We appreciate this accurate observation. We agree that the phrase "only in the absence" was an overstatement and have clarified our selection criteria.

**1. Correction of Text:** We acknowledge that many candidates showed quantitative reduction rather than exclusive binding. Accordingly, we have revised the text in the Results section (Line 358) to describe the data more precisely: *"...identifying several ATOH1-binding molecules whose interaction with ATOH1 was disrupted or much reduced in the presence of MEIS1-FL."*

**2. Selection Criteria (PANTHER GO Analysis):** Regarding the selection of the reported proteins, we utilized a hypothesis-driven approach to identify regulators of degradation. Specifically, we screened the list of proteins whose binding was reduced by MEIS1-FL using PANTHER Gene Ontology (GO) analysis. We specifically filtered for proteins annotated with Gene Ontology (GO) terms or Protein Classes related to the ubiquitin-proteasome system. From this objective screening, CUL3 and its partners (UBE2M, CAND1) emerged as top candidates. We have added this information to the Results text and Figure 5D Legend to clarify the selection process.

**Comment 9**

The same issue also applies to the mass spec. experiments discussed in lines 253/254. How were the proteins listed in Fig.6A selected? All in all, it has to be made transparent in the manuscript, how the analysis of the mass spec. results was performed. Also, how often were the MS experiments repeated? Were the data uploaded to a public repository?

**Response:** We have clarified the selection process, experimental repeats, and data availability.

**1. Selection Criteria (Hypothesis-Driven):** The selection of proteins in Figure 6A was hypothesis-driven. Our primary goal was to understand how MEIS1 inhibits CUL3 function. Since the COP9 Signalosome (CSN) is the canonical regulator known to bind and inhibit Cullin-RING ligases, we specifically screened our GST pull-down MS candidate list (1417 proteins) for CSN subunits (COPS proteins). We focused on these proteins not arbitrarily, but because they provided a direct molecular mechanism explaining the MEIS1-dependent inhibition of CUL3. We have ensured the Results text reflects this logic.

**2. Experimental Repeats and Validation:** The LC-MS/MS analysis was performed as a screening experiment (n = 1) to identify potential interacting partners. Crucially, the reliability of this screening was rigorously confirmed by subsequent validation experiments:

- The interaction with CSN components identified by MS was validated by Western blotting (Figure 6B).
- Furthermore, we confirmed the interaction endogenously in GCP lysates (New Figure 6D). These validation steps confirm that the MS results represent genuine biological interactions.

3. Data Availability: Regarding data transparency, we have provided the complete list of all 1417 identified proteins, including their score parameters, as a Supplementary Excel File attached to the manuscript.

**Comment 10**

Lines 316/137. The authors state 'In contrast to MEIS1-FL, of which large amounts were required to suppress ATOH1 degradation, this was achieved using very little MEIS1-HdL (Fig.6H, I), suggesting that MEIS1-HdL suppresses ATOH1 degradation more effectively than MEIS1-FL.'. There are at least two alternative explanations for this difference. First, it can be purely technical. Smaller proteins are more effectively expressed than larger ones. Because MEIS1-HdL is smaller than MEIS1-FL it may be expressed at higher levels and hence have a more profound effect. Since the authors do not show the MEIS1-FL or MEIS1-HdL inputs in any of their figures this cannot be judged. Second, the MEIS2 C-terminus folds back onto the N-terminus, masking the C-terminally located transactivation domain and leading to transcriptional autoinhibition (Hyman-Walsh et al., FEBSJ, 2010). If the N-terminus of MEIS1 plays a role in protein ubiquitination, folding of the C-terminus onto the N-terminus could also affect this function.

**Response:** We deeply appreciate these insightful comments. We fully agree with both alternative explanations provided by the reviewer.

**1. Technical Explanation (Expression Levels) and Text Revision:** We acknowledge that smaller proteins like MEIS1-HdL are often expressed more efficiently than full-length proteins. As mentioned in our Response to Comment 6, we have included Input blots in the revised figures to allow for the direct verification of expression levels in each experiment. Acknowledging the reviewer's point that plasmid dosage does not strictly reflect protein amount, we agree that concluding "HdL is biochemically stronger" was premature. Accordingly, we have removed the claim that "MEIS1-HdL suppresses ATOH1 degradation more effectively" from the Results section. Instead, we have revised the text to strictly describe the observation regarding plasmid dosage: *"This indicates that MEIS1-HdL is capable of suppressing ATOH1 degradation even at low plasmid dosages, whereas MEIS1-FL requires higher dosages to achieve a similar effect in this assay."*

Furthermore, as detailed in our Response to Comment 4, we now emphasize that the differential subcellular localization is the primary determinant of their distinct functions *in vivo*. As shown in New Figure 2C and S2B, MEIS1-HdL is the predominant isoform in the cytoplasm (where CUL3 resides), whereas MEIS1-FL is sequestered in the nucleus. This spatial separation explains why HdL serves as the functional stabilizer *in vivo*.

**2. Structural Explanation (Autoinhibition):** The reviewer’s point regarding the autoinhibitory folding of the C-terminus (as seen in MEIS2) is scientifically fascinating. Since the interaction with CUL3/CSN involves the N-terminal region of MEIS1, it is highly plausible that in MEIS1-FL, the C-terminal Homeodomain might spatially mask the N-terminus, whereas MEIS1-HdL (lacking the C-terminus) remains in an "open" conformation, facilitating the interaction. We have incorporated this valuable insight into the Discussion section (LINE 549), citing Hyman-Walsh et al. (2010), as a potential structural mechanism contributing to the functional difference.

**Comment 11**

MEIS1 is endogenously expressed at high level in many neuroblastoma cell lines. If this were also the case in N2A cells, any pulldown or IP experiments that the authors perform without co-transfection of MEIS1-FL would still occur in the presence of cellular MEIS1. The authors need to assess the expression of endogenous MEIS1 (and potentially other genes that they overexpress) in N2A cells to clarify this issue. If MEIS1 is endogenously expressed in N2A cells, the degree of overexpression after transfection of MEIS1-FL / MEIS1-HdL should also be measured and discussed.

**Response:** We appreciate this important check regarding the cellular background. We have assessed the endogenous MEIS1 expression in our N2a cells.

As demonstrated in Figure S2A (antibody validation), we included a negative control condition where N2a cells were transfected with an Empty Vector. In these lysates, neither the pan-MEIS1 antibody nor the HdL-specific antibody detected any specific bands corresponding to MEIS1-FL or MEIS1-HdL, whereas robust signals were detected in the overexpression lanes. This confirms that the endogenous expression of MEIS1 in the N2a cell line used in this study is negligible or below the detection limit of Western blotting. Therefore, the interactions and functional effects observed in our experiments can be attributed to the exogenously introduced MEIS1 isoforms, without interference from an endogenous pool.

**Comment 12**

The information about the experimental procedures given in the Mat&Meth chapter is not sufficient. (1) In line 412 the authors state 'The expression vectors of Atoh1, Meis1 1-130aa and Meis1-FL were cloned from cDNAs from C57/BL6 mouse cerebellum.' How was this done ? From embryonic or adult tissue? What primers were used? Which polypeptides do the cloned sequences correspond to? It is also not clear how Meis1-HdL was cloned. (2) The GST pull down experiments cannot be reproduced. Where were cell harvesting and lysis described? What is the composition of TED buffer? The reference to Nagai et al, 2016 is missing from the reference list. (3) Lines 271/272: The authors performed HA-IP followed by GST-PD, but the Mat&Meth chapter lacks any reference to this procedure.

**Response:** We apologize for the insufficient description of the experimental procedures. We have significantly expanded the Materials and Methods section to ensure reproducibility.

**1. Cloning Details:** We have specified that cDNAs were amplified from P7 mouse cerebellum. We have provided the specific primer sequences used for cloning Atoh1, Meis1-FL, and Meis1-HdL in the updated Materials and Methods section. We also clarified that Meis1-HdL was cloned via RT-PCR using isoform-specific primers targeting the full-length transcript.

**2. GST Pull-down and Buffer Compositions:** We have added the detailed composition of the buffers to the text. specifically:

- Lysis Buffer: Cell Lysis Buffer (Nacalai Tesque) or Pierce™ IP Lysis Buffer (Thermo Scientific).
- **TED Buffer:** 50 mM Tris-HCl pH 8.0, 10 mM reduced glutathione, 1 mM DTT, 1 mM EDTA. We have also corrected the missing reference to Nagai et al. (2016) in the bibliography.

**3. Sequential Precipitation:** We have added a distinct description for the sequential precipitation (HA-IP followed by GST-PD) procedure. We clarified that lysates were first incubated with anti-HA antibody and precipitated using Protein G Sepharose beads. The complexes were eluted with HA peptide and subsequently captured by glutathione-Sepharose beads.

**Comment 13**

Information on the number of repeat experiments is missing throughout. For example, how many cells per specimen were quantified in Figs.2, 3, 7? How often were the biochemical experiments repeated? How often the mass spec experiments?

**Response:** We apologize for the lack of clarity. We have updated the Figure Legends and the Materials and Methods section to explicitly state the sample sizes and number of replicates.

**1. Quantification in Figures:** We have added the specific n numbers to the Figure Legends for all quantification data.

**2. Biochemical Experiments:** We have explicitly stated in the Materials and Methods section that all Western blotting and co-immunoprecipitation images presented in this study are representative of at least three independent experiments (n ≥ 3).

**3. Mass Spectrometry Experiments:** We have clarified in the Materials and Methods section that the LC-MS/MS analysis was performed as a screening experiment (n = 1 biological replicate). Crucially, the key interactions identified by this screen were rigorously validated by independent Western blotting and Co-IP experiments (n ≥ 3) presented in the main figures.

**Response to Reviewer #3, Minor Points**

**[Minor Points & Text Clarifications]**

**Point 1:** The authors leave open, which of the known MEIS1 isoform they call MEIS1-FL. In line 93 they mention that MEIS1-FL comprises 390aa. This suggest that their MEIS1-FL is isoform B (NP_001180200.1), but this must be clearly stated.

**Response:** We appreciate this request for precision. The reviewer is correct; our "MEIS1-FL" (390 aa) corresponds to mouse Meis1 isoform 2 (NCBI Reference Sequence: NP_001180200.1). In the revised manuscript, we have explicitly stated this accession number in the Results section to avoid ambiguity (Lines 199). Additionally, in New Figure 1, we have indicated the corresponding Ensembl Transcript ID (ENSMUST00000068264.14) derived from our Nanopore sequencing analysis, which confirms the identity of the full-length isoform used in this study.

**Point 2:** Line 202/203: A marked effect of Cul3-knockdown on ATOH1 is not visible in Fig.5G.

**Response:** We apologize that the effect was not sufficiently clear in the previous blot due to suboptimal image quality. We have repeated the experiment and replaced the panel with a high-quality representative blot in the revised Figure 5G. The new data clearly demonstrates the significant accumulation of ATOH1 protein following *Cul3* knockdown, consistent with our conclusion.

**Point 3:** Line 210: The authors state 'Particularly, S328 phosphorylation was markedly increased (Fig.S5B).' Fig.S5B is a schematic drawing and thus cannot be used as evidence for this argument.

**Response:** We apologize for the confusion caused by the figure presentation. The figure in question (now Figure S8B) is not a mere theoretical schematic, but a visual representation based on the analysis of MS signal intensities, where the color intensity reflects the relative increase in phosphorylation levels observed in the experiment. The detailed data supporting this "marked increase" is provided in the Supplementary Excel File. To clarify this in the revised manuscript:

1. We have revised the text to explicitly state that "analysis of MS signal intensities indicated" the increase, citing the Supplementary Data as the primary evidence.
2. We have updated the Figure Legend of S8B to clearly state that it depicts the MS analysis results.
3. Furthermore, we have provided validation data in Figure S8C, where the increase in S328 phosphorylation is confirmed using a specific anti-phospho-S328 antibody.

**Point 4:** Lines 263/264: 'Immunoblotting followed by LC-MS/MS did not reveal the presence of COPS5, a known component of the CSN complex, indicating that both MEIS1 isoforms interacted with endogenous COPS5 (Fig.6B).' The authors need to explain this statement better.

**Response:** We apologize for the confusing and logically flawed phrasing in the original manuscript. The reviewer is correct that the previous statement was misleading. In the revised manuscript, we have removed the confusing sentence and restructured the section to accurately describe the findings (Lines 423-435).

1. We first list the specific CSN components identified by the LC-MS/MS screening (COPS2, 3, 4, 6, 7a, and 8).
2. We then describe the subsequent Western blotting results, which confirmed the interaction with COPS5 (as well as COPS2) (Fig. 6B). This revision eliminates the ambiguity and clearly presents COPS5 detection as a result of the targeted validation step.

**Point 5:** The schematic in Fig.1.B seems to be wrong. The upstream primers for FL and HdL are not identical and are not located in exon6 but in exon 7

**Response:** We apologize for the error in the schematic diagram in the previous manuscript. To address this issue and ensure the highest specificity, we have redesigned the primer sets and re-performed the RT-PCR experiments (now presented in New Figure 2B).

- For MEIS1-FL, we designed a primer targeting Exon 8.
- For MEIS1-HdL, we utilized a primer targeting the specific sequence used for cDNA cloning. The precise sequences of these primers are listed in the revised Materials and Methods section.

**Point 6:** Labeling of the X-axes in Fig. 1E. Please be more specific: Meis1 transcripts were normalized to what?

**Response:** We assume the reviewer refers to the normalization method for the relative expression levels (Y-axis values). In this experiment, the gene expression levels were normalized to 18s rRNA (internal control). To make this clear, we have updated the Figure Legend to explicitly state: "Relative mRNA expression of Meis1-FL and Meis1-HdL (norm. to 18s rRNA)".

**Point 7:** Fig.5B: How exactly was ATOH1 ubiquitination measured?

**Response:** We quantified the ubiquitination levels by measuring the total signal intensity of the high-molecular-weight smear extending above the unmodified ATOH1 band using ImageJ software. The background signal was subtracted from each measurement. Since we ensured that comparable amounts of ATOH1 were immunoprecipitated across samples (as shown in the anti-ATOH1 blot), we directly compared the smear intensities to assess the extent of polyubiquitination.

**Point 8:** Fig.5SF: What is 'maximum level of ATOH1'? and how can this reach values about 100%? Do the authors mean 'band intensitis relative to ATOH1 band intensity at 0h'?

**Response:** Yes, the reviewer is correct. We meant the band intensity relative to the intensity at the 0-hour time point (set as 100%). We apologize for the confusing labeling. We have corrected the Y-axis label in Figure S8F (formerly S5F) to "Relative ATOH1 level (% of 0 h)" to clearly indicate that the values represent the protein stability relative to the starting time point.

**Point 9:** The nomenclature for gene and protein names should be reviewed again.

**Response:** We have thoroughly reviewed the manuscript and standardized the nomenclature for all genes and proteins according to the official guidelines (e.g., *Italics* with the first letter capitalized for mouse genes, and Roman all-caps for proteins).

**Point 10:** The figure captions would benefit from more detailed descriptions.

**Response:** We have carefully reviewed and revised the Figure Legends throughout the manuscript to ensure that the legends are informative and self-explanatory.
