# Supplementary material for: Meis1 isoform diversity orchestrates neural progenitor differentiation by regulating ATOH1 degradation at distinct subcellular compartments
Source: PLoS Biol. 2026 Jul 13;24(7):e3003897. doi: 10.1371/journal.pbio.3003897 (PMC13379096; doi:10.1371/journal.pbio.3003897)
Supplement: Attachment — Submitted filename: Response-to-Reviewes_Revise_v2.docx [file pbio.3003897.s020.docx]

**Overview of the Revised Manuscript**

We thank the editors and reviewers for their careful and constructive evaluation of our manuscript. In this revision, we performed additional experiments and substantially revised the manuscript to address the remaining concerns. In particular, we added a nuclear-targeted NLS-MEIS1-HdL experiment to test the importance of cytoplasmic localization for ATOH1 stabilization, and we added a co-immunoprecipitation analysis examining PBX3 in the MEIS1-HdL/CSN complex. We also strengthened the validation of the MEIS1-HdL-specific antibody using endogenous GCP lysates, clarified the interpretation of the TPM > 1 filter as a screening criterion rather than definitive evidence of function, and revised the Results and Discussion to distinguish more clearly between conclusions directly supported by the data and mechanisms that remain unresolved. In addition, we updated the figures, figure legends, Materials and Methods, and supplementary data files to improve clarity and reproducibility. Below, we provide a detailed point-by-point response to each reviewer comment.

**Point-by-Point Response to Reviewer**

**Response to Reviewer #1**

**Comment 1.1**

The revised manuscript is greatly improved compared to the original submission, and I appreciate the detailed responses to the reviewers comments, the inclusion of many new experiments, and the more thoughtful interpretations of the data. My one remaining concern is how definitively the authors have demonstrated that MEIS1 functions outside of the nucleus, and not as a transcription factor. Subcellular localization studies are not definitive, especially in cases like this where the localization is not black and white – there always seems to be some signal in the nucleus making it impossible to rule out a nuclear (transcriptional) function. Similarly, protein-protein interactions with other cytoplacmic proteins are also not definitive since, even if they exist, they do not prove that these interactions are required for MEIS1 function. What data can the authors point to that definitively rule out a nuclear function for the affects on ATOH1 stability?

**Response:** We agree that localization alone is not sufficient to establish the functional site of action. To test this directly, we generated an NLS-MEIS1-HdL construct that shifts MEIS1-HdL predominantly to the nucleus. In this condition, the ATOH1-stabilizing activity of MEIS1-HdL was attenuated. Because a small cytoplasmic fraction of NLS-MEIS1-HdL remained detectable, we interpret these data conservatively as strong functional evidence that cytoplasmic localization is required for the full ATOH1-stabilizing activity of MEIS1-HdL, while minor nuclear contributions cannot be formally excluded.

　These changes are described in the Results section interpreting the NLS-MEIS1-HdL experiment and the PBX3 analysis (Fig. S10A–D), and in the corresponding Discussion text, where we now explicitly state that minor nuclear contributions cannot be formally excluded.

**Comment 1.2**

For example, can the authors disrupt a key protein interaction with a cytoplasmic protein and show that this abolishes MEIS1 function?

**Response:** We agree that a separation-of-function mutant that selectively disrupts the MEIS1-CSN interaction would be the most direct experiment. Although we have not yet identified such a mutant, our current data support the functional requirement of the CSN pathway for MEIS1 activity in two ways. First, knockdown of Cops6 significantly attenuated the ability of MEIS1 to suppress ATOH1 polyubiquitination. Second, forced nuclear enrichment of MEIS1-HdL reduced its ATOH1-stabilizing activity. Together, these results support a model in which the cytoplasmic MEIS1-HdL-CSN-CUL3 axis is required for the full stabilizing effect on ATOH1, while we acknowledge that the exact binding interface remains to be defined.

These revisions are described in the Results section discussing the *Cops6* knockdown and NLS-MEIS1-HdL experiments, together with the corresponding figure legends for Fig. 6G and Fig. S9D/S10A–C.

**Comment 1.3**

Along the same lines, the authors do not consider interactions between MEIS1 and its well-known transcriptional partner PBX as a potential mechanism for either FL or HdL isoform function. Are PBX isoforms expressed in these cells? Can the authors demonstrate that PBX has no function here?

**Response:** We appreciate this important point. Because MEIS proteins classically act together with PBX factors, we agree that it is important to address PBX expression and potential involvement in this mechanism. We re-examined our dataset and confirmed that PBX family transcripts are detectable in these cells. Among them, Pbx3 was the most abundant PBX family transcript in our long-read dataset, and we therefore selected PBX3 as the most relevant candidate for further analysis.

We have now clarified this point in the revised Results section. We then asked whether PBX participates in the MEIS1-HdL/CSN mechanism described here. In our new co-immunoprecipitation assay, PBX3 was not detected in HA-COPS6 precipitates under conditions in which the MEIS1-HdL/CSN interaction was observed (Fig. S10D). These data indicate that PBX3 is not a detectable component of the MEIS1-HdL/CSN complex. We therefore describe this mechanism more conservatively as PBX3-independent at the level of the detected complex, while not excluding broader PBX-dependent functions in other contexts.

These changes are described in the Results section introducing the PBX3 analysis, in Fig. S10D and its legend, and in the corresponding Discussion paragraph.

**Comment 1.4**

Once the authors address this one remaining concern and discuss alternative mechanisms that are not definitively ruled out by their experiments, this paper will make a valuable contribution to the field.

**Response:** We sincerely appreciate the reviewer’s constructive suggestion. In response, we revised both the Results and the Discussion to more clearly distinguish what is directly supported by our data from what remains unresolved. Specifically, our new NLS-MEIS1-HdL experiment supports the importance of cytoplasmic localization for the full ATOH1-stabilizing activity of MEIS1-HdL, and our new co-immunoprecipitation analysis indicates that PBX3 is not a detectable component of the MEIS1-HdL/CSN complex. At the same time, we now explicitly acknowledge that our current study does not formally exclude additional nuclear or transcription-dependent contributions of MEIS1, nor broader PBX-dependent functions outside of the detected complex. We believe these revisions provide a more balanced interpretation of our findings and strengthen the manuscript.

These revisions were made in both the Results and Discussion sections, particularly in the paragraphs interpreting the NLS-MEIS1-HdL and PBX3 experiments.

**Response to Reviewer #2**

We thank Reviewer #2 for the careful evaluation of our manuscript and are grateful that the reviewer had no additional comments.

**Response to Reviewer #3**

**Comment 1**

Evidence for the existence of an HD-less Meis1 transcript variant: The authors performed nanopore sequencing to demonstrate that multiple, previously unknown transcript variants exist. This is very interesting but information on their frequency is missing. Please provide the number of times each of the transcript variants depicted in Fig. 1F were detected. This is important because very low-frequency splice junctions can be nonfunctional. For instance, it has been estimated that splicing errors occur at a rate of approx. 0.7% per intron. Novel isoforms detected at 0-1 TRM can therefore result from 'noisy splicing'. This concern also applies to the argument made in lines 131-133 ("To focus on robustly expressed isoforms, we applied a stringent filter, retaining only those transcripts with a TPM value greater than 1 in each of our three replicates"). Greater than 1TPM is still a relatively mild cut-off, given that on average 2% of transcripts from an average gene can be mis-spliced. It would strengthen the authors' argument if there was some evolutionary conservation, e.g. with the isoform described for hth by Noto et al., Genes & Dev. 2006 (see also argument 14 below).

**Response:** We thank the reviewer for this important point. We agree that transcript abundance alone does not establish biological relevance and that low-frequency splice products can reflect splicing noise. We therefore revised the manuscript in four ways. First, we now provide the absolute read counts for each Meis1 transcript variant in S2 Data, together with the corresponding nucleotide and predicted amino acid sequences. Second, we have toned down our description of the TPM > 1 filter and now present it as a screening criterion for candidate isoforms rather than definitive evidence of function. Third, we expanded the Discussion to place our findings in the context of previously reported homeodomain-less Meis-family isoforms, including the work of Noro et al. Fourth, because transcriptomic evidence alone is insufficient, we emphasize the orthogonal validation provided by isoform-specific protein detection and the *in vivo* knockdown/rescue experiments. Together, these data strongly argue that MEIS1-HdL is a bona fide expressed isoform rather than a low-level splicing artifact.

These changes are reflected in the Results section describing transcript filtering and Meis1 isoform identification, in Fig. 1 and its legend, in S2 Data, and in the Discussion where we now discuss the relevant homeodomain-less Meis-family literature.

**Comment 2**

Please provide the sequence of the transcript variants identified and the aa sequence of the isoforms translated from them shown in Fig. 1F, G.

**Response:** As requested, we have provided the complete nucleotide sequences of the identified Meis1 transcript variants (shown in Fig. 1F) and their corresponding translated amino acid sequences (shown in Fig. 1G). These sequences are provided in S2 Data, and the Results section describing Fig. 1F, G now explicitly cites S2 Data.

**Comment 3**

Lines 193-194: "To test this, we generated a specific antibody against the unique 5-amino acid C-terminus of the predicted homeodomain-less isoform." Without sequence comparison between MEIS1-FL and MEIS1-HdL as well as information on how the MEIS1-HdL-specific antibody was generated, it remains obscure why the a-MEIS1 antibody (directed against the FL protein) and the a-MEIS1-HdL antibody (according to the authors distinct from MEIS1-FL due to a frameshift, line 564) recognize similar size bands in P7 GCPs (arrows in Fig. 2A). Where do the two proteins differ? Which antigens were used to generate the antibodies? Antibody production, including procedure and antigene used, is not mentioned in the Mat&Meth chapter.

**Response:** We appreciate this request for clarification. To make the relationship between MEIS1-FL and MEIS1-HdL explicit, we have now clarified both the sequence difference between the two proteins and the generation of the isoform-specific antibody. Skipping of exon 8 generates an exon 7–9 junction, which causes a frameshift and replaces the canonical C-terminal homeodomain-containing region with a unique short C-terminus in MEIS1-HdL. We generated the MEIS1-HdL-specific antibody against this frameshift-derived peptide (NH2-C-(Ahx)-EQAPLPF-COOH), and we have now added the antibody-generation and purification procedures to the Materials and Methods subsection “Generation of the MEIS1-HdL specific antibody.” We also revised the Results text around Fig. 2A and the relevant figure presentation to clarify why the commercial pan-MEIS1 antibody detects both isoforms whereas the custom antibody selectively detects the HdL-specific neo-epitope.

**Comment 4**

Fig.2A does not proof beyond doubt that the MEIS1-HdL antibodies do not recognize any unrelated protein(s) in the extracts. Where does the second band come from that is recognized by MEIS1-FL and MEIS1-HdL antibodies? Validating antibodies with the overexpressed polypeptide that was used to immunize the rabbits is also a circular argument (Fig. S2A). Can the authors provide better proof of the specificity of their MEIS1-HdL abs? A simple approach would be to re-probe the blot shown in Fig. S2B with their custom-made antibody.

**Response:** We agree that validation against overexpressed material alone is insufficient. We therefore added two orthogonal validations using endogenous GCP lysates. First, in the revised endogenous blot shown in Fig. 2A, re-probing with the MEIS1-HdL-specific antibody selectively detected a single lower-molecular-weight band, whereas the upper band corresponding to MEIS1-FL was no longer detected. Second, endogenous proteins were immunoprecipitated from P7 GCP lysates with the MEIS1-HdL-specific antibody and detected by immunoblotting with a pan-MEIS1 antibody, yielding a single band at the expected mobility (Fig. S2B). Together, these results strongly support the specificity of the custom antibody for endogenous MEIS1-HdL and indicate that the HdL-specific signal in the revised blot corresponds to a single endogenous lower-molecular-weight species. These additions are now described in the Results section for Fig. 2A and in the legend for Fig. S2B.

**Comment 5**

Lines 585/586: The authors state "Nanopore analysis definitively identified the exact full-length transcript corresponding to the MEIS1-HdL cDNA we cloned and used for overexpression." How does this relate to the statement that MEIS1-HdL contains a frameshift, which enabled the authors to generate isoform-specific antibodies? Is the frameshift contained in the overexpressed cDNA?

**Response:** We apologize for the ambiguity caused by the phrase “full-length transcript.” In our previous response, this term referred to a complete Nanopore long-read covering the entire MEIS1-HdL cDNA sequence, not to the canonical full-length MEIS1-FL protein isoform. The cloned MEIS1-HdL cDNA used for overexpression contains the exon 7–9 junction and therefore includes the same frameshift that generates the unique HdL-specific C-terminus recognized by our antibody.

The wording in the revised Results text has been clarified accordingly so that the Nanopore-defined full-length transcript is not confused with the canonical full-length protein isoform.

**Comment 6**

Line 263: "Overexpression of Meis1-FL significantly decreased the proportion of GCPs". How was the quantification done?

**Response:** We apologize for not describing the quantification method clearly. In all in vivo electroporation experiments, an H3.1-GFP plasmid was co-electroporated to label the nuclei of transfected cells. For each section, the percentage of marker-positive cells was calculated as the number of GFP-positive electroporated cells co-expressing KI67, p27, or ATOH1 divided by the total number of GFP-positive electroporated cells within the analyzed cerebellar layers. Counting was performed using ImageJ on multiple sections from at least three independent animals.

This information has now been added to the Materials and Methods and clarified in the relevant figure legends for the in vivo electroporation experiments.

**Comment 7**

Lines 332/333 and lane 439: MEIS1(1-130) and MEIS1-HdL must be more clearly distinguished from one another. What was the rationale of including MEIS1(1-130)?

**Response:** We agree that this distinction needed to be clarified. MEIS1-HdL is an endogenous homeodomain-less isoform that retains the N-terminal MEIS homology region, including the MH-B domain, whereas MEIS1(1-130) is an artificial truncation generated for domain-mapping purposes and lacks both the MH-B domain and the homeodomain. We included MEIS1(1-130) to test whether the region downstream of amino acid 130 is required for ATOH1 stabilization and COPS6 binding.

These distinctions are now clarified in the Results section describing the stabilization assays and in the corresponding descriptions of the MEIS1(1–130) construct.

**Comment 8**

Line 380, Fig. 5G: The graph in Fig. 5H contains data for GST-ATOH1 w/o CHX, but the respective blot is not shown in Fig. 5G.

**Response:** We thank the reviewer for pointing out this inconsistency. We agree that the previous presentation was not sufficiently clear. We therefore reorganized the CHX chase experiment using sh-Scramble as the matched control and sh-*Cul3* in parallel, and we updated both the representative blot in Fig. 5G and the quantification in Fig. 5H accordingly. The Results text and figure legend have been revised to make this direct comparison explicit.

**Comment 9**

Line 403, Fig. S8D-F: The values for GST-ATOH1S238D (without co-transfection of MEIS1-FL or MEIS1-HdL) are not included in the graph in Fig.S8F. Conversely, the blots for GST-ATOH1 are not shown. The latter is important, because the biological relevance of the data in Fig. S8F depends on the comparison with the baseline values of GST-ATOH1 (black).

**Response:** We thank the reviewer for this important point. We agree that interpretation of the phosphomimetic mutant requires an explicit comparison with the WT baseline. We therefore added new panels comparing the steady-state levels of WT ATOH1 and ATOH1-S328D (Fig. S8E, F), which show the markedly reduced baseline abundance of the S328D mutant. We also clarified in the Fig. S8H legend that the representative WT control blot used for the quantification is shown in Fig. S7B. Because the starting level of ATOH1-S328D alone is very low, a normalized CHX decay curve for this condition would be difficult to interpret and potentially misleading. For this reason, we document its instability primarily by the new baseline comparison in Fig. S8E, F and use the CHX analysis in Fig. S8G, H to test whether MEIS1 can stabilize this unstable phosphomimetic mutant.

These revisions are now incorporated into the Results section describing the S328 phosphorylation experiments and in the updated legends for Fig. S8E–H.

**Comment 10**

Line 435, Fig. 6B: Please comment on why COPS5 was tested in GST-PD assays when it was not identified my LC-MS/MS. Is this a sign that all COP9 signalosome components bind to MEIS1 or that they are generally 'sticky' towards the MEINOX homology domain? Can the authors provide a control TF, fused to GST, that does not bind to one of the COP9 complex subunits?

**Response:** We appreciate this important concern. We selected COPS5 for validation because it is the catalytic core of the CSN holo-complex; therefore, detection of COPS5 binding would support recruitment of the functional CSN complex even though COPS5 was not recovered in our discovery LC-MS/MS dataset, likely owing to peptide-detection limitations. We have clarified this rationale in the revised Results section.

We agree that a GST-fused unrelated transcription factor would be an additional useful specificity control. Although we have not added that exact experiment in this revision, several observations argue against generalized non-specific stickiness: GST alone did not pull down COPS proteins, the artificial MEIS1(1-130) truncation lost COPS6 binding while GST-MEIS1-FL and GST-MEIS1-HdL retained it, and in an orthogonal co-immunoprecipitation assay PBX3 was not detected in HA-COPS6 precipitates. We therefore interpret the current data as supporting a selective interaction between MEIS1 and the CSN complex, while acknowledging that the precise binding interface and broader specificity remain to be defined.

This rationale is now stated in the Results section introducing Fig. 6B, and the interpretation has been moderated accordingly in the revised text.

**Comment 11**

Reference to figure S9D is missing from the text

**Response:** We apologize that this cross-reference was not sufficiently clear. Figure S9D is now explicitly cited in the Results text describing the Cops6 knockdown experiment, and we also added a cross-reference in the Fig. 6G legend to guide the reader to the knockdown validation more easily.

**Comment 12**

Lines 493/494: The authors have softened their claim that "MEIS1-HdL suppresses ATOH1 degradation more effectively than MEIS1-FL" to "MEIS1-HdL is capable of suppressing ATOH1 degradation even at low plasmid dosages". However, this does not fully alleviate the concern that the observed difference may be largely technical. This concern also applies to the statement "However, it was shown that MEIS1-HdL has a much stronger ATOH1-stabilization ability than MEIS1-FL." in the Discussion (lines 548/549).

**Response:** We appreciate this important point and agree that the comparison between MEIS1-FL and MEIS1-HdL in overexpression assays should be interpreted cautiously. We therefore further softened our wording in both the Results and Discussion. Rather than concluding that MEIS1-HdL is intrinsically stronger than MEIS1-FL, we now describe the data more conservatively as an apparent difference in dose responsiveness in this assay. We also note explicitly that construct-dependent technical factors cannot be fully excluded. At the same time, the new NLS-MEIS1-HdL experiment supports the idea that subcellular localization contributes substantially to this apparent difference, because forcing MEIS1-HdL into the nucleus attenuated its ATOH1-stabilizing effect despite only minimal changes in construct size. These revisions are reflected in the Results section discussing Fig. 6H, I and Fig. S10A–C, as well as in the corresponding Discussion paragraph.

**Comment 13**

Lines 503-503: "This difference in intracellular localization between MEIS1-FL and MEIS1-HdL probably accounts for the differential efficacy in suppressing CUL3-dependent ATOH1 degradation." The authors previously argue that "ATOH1 is polyubiquitinated by CUL3 in the cytoplasm, probably just after ATOH1 protein synthesis, while ATOH1 polyubiquitination in nuclei is elicited by other E3 ligases." This is a plausible argument for the idea that ATOH1 degradation in the cytoplasm and nucleus is driven by different processes, but it does not explain why degradation should be more efficient one subcellular compartment than the other. To answer that, one would need to know which E3 ligase regulates ATOH1 ubiquitination in the nucleus and how that differs from CUL3.

**Response:** We agree with the reviewer that our previous wording was overly speculative and could be misread as a comparison of degradation efficiency between the cytoplasm and nucleus. That was not our intention. We have therefore revised the text to remove the unnecessary speculation about distinct nuclear E3 ligases and to frame our interpretation more precisely in terms of spatial access to the cytoplasmic CUL3/CSN machinery. Our conclusion is not that cytoplasmic degradation is inherently stronger than nuclear degradation, but rather that the predominantly cytoplasmic MEIS1-HdL is better positioned to interfere with the CUL3-dependent pathway detected in our assay.

These revisions are reflected in both the Results and Discussion, where we now interpret the difference between the isoforms primarily in terms of differential access to the cytoplasmic CUL3/CSN machinery.

**Comment 14**

Line 551: If the authors want to include the publication by Hyman-Walsh into their argument, they need to consider that MEIS-autoinhibition is regulated by heterodimerization by PBX1. When the contribution of PBX1 to GC differentiation is unknown, this must be stated or the argument dropped. Other published work which the authors may want to consider: 1. Noro et al., Genes & Dev 2006, report a homeodomain-less isoform of hth that is functionally different from the full-length protein, as well as a HD-less isoform of Meis1 from mouse. It would be informative if the MEIS1-HdL isoform described here was compared against the isoform of Noro et al.. 2. MEIS2 is proteolytically cleaved by calpain proteases resulting in an HD-less cleavage product (Muller et al., JCS 2025).

**Response:** We thank the reviewer for these important suggestions. In response, we revised the Discussion in two ways. First, we avoided overextending the argument about MEIS autoinhibition in the absence of direct evidence for PBX involvement in this specific mechanism. Instead, we now describe our PBX result more conservatively: PBX3 was not detected in the MEIS1-HdL/CSN complex in our co-immunoprecipitation assay, which argues against its direct participation in the detected complex but does not exclude broader PBX-dependent functions in GCP biology. Second, we incorporated the suggested literature to better contextualize our findings, including the homeodomain-less hth/Meis-family isoform literature (Noro et al.), the proteolytically generated homeodomain-less MEIS2 product (Muller et al.), MEIS2 splice variants in neuroblastoma, and alternative splicing of Pax6.

These literature and interpretation changes were incorporated into the Discussion, particularly in the paragraph addressing MEIS autoinhibition, PBX, and related homeodomain-less MEIS-family isoforms.

**Comment 15**

Lines 571/572, temporal expression of MEIS1-FL during GC development: Please give citation.

**Response:** We appreciate this suggestion. Because this statement is based on our own data rather than previous literature, we have now added an explicit citation to Fig. 2F in the Discussion.

**Comment 16**

Line 599, MEIS1 enhances the proliferation of hematopoietic stem cells: This sentence is oversimplified. Leukemia arises from differentiation arrest and self-renewal, not simply increased proliferation.

**Response:** We agree and have revised this sentence to more accurately reflect leukemogenic mechanisms. It now states that MEIS1 overexpression promotes aberrant self-renewal and differentiation arrest in hematopoietic stem and progenitor cells, rather than simply increasing proliferation.

　This sentence has been revised in the Discussion section dealing with the broader biological significance of MEIS1.

**Comment 17**

Lines 604-617: Glutamate receptor genes are a good example of how differential splicing of a gene produces functionally distinct isoforms, but this is somewhat of a stretch in the present context. Examples of differential splicing of transcription factors would be more appropriate. For instance, there is extensive literature on alternative splicing of Pax6, which the authors mention at the beginning of their study. There are also examples for functional differences between MEIS-family proteins, such as Noro et al. mentioned above, or MEIS2 in neuroblastoma (Gross et al., Cancer Res 2018).

**Response:** We agree with the reviewer and have removed the glutamate receptor example. We rewrote the final Discussion paragraph to focus instead on transcription-factor isoforms and related regulatory mechanisms, incorporating the suggested examples of hth/Meis-family homeodomain-less isoforms, MEIS2 variants, and Pax6 alternative splicing. We believe these changes place MEIS1-HdL in a more appropriate and relevant conceptual context.

This revision was made in the final paragraph of the Discussion, which now focuses on transcription-factor isoforms and related regulatory mechanisms.

**Comment 18**

The graphs look different. Some show individual data points, others show only error bars; sometimes statistical significance is indicated by a horizontal line, and sometimes only by asterisks above the bar. Please unify this.

**Response:** We apologize for the inconsistent graph formatting. We have now standardized the presentation of quantitative data throughout the manuscript and supplementary figures, including the display of individual data points, error bars, and the style of statistical annotations.

These presentation changes were made throughout the main figures and supplementary figures.

**Comment 19**

Fig. R1 was intended for the review process only, but there are still two points that need to be noted: 1. Panel A: Why does lane 2, but not lane 1 show a band at approximately 50 kDa, even though the same plasmids were transfected? 2. Panel B: Where does the GFP fluorescence come from? None of this is clear from the figure legend. This reflects an issue that was of considerable concern in the initial submission, has improved in the current submission, but has still not been fully resolved: The experimental approach is described so briefly and, in some cases, so superficially that the text becomes difficult to understand.

**Response:** We thank the reviewer for pointing out that the presentation of Fig. R1 was unclear. The issue arose because the lane annotation indicating MEIS1-FL overexpression was inadvertently omitted from the figure. In this experiment, lanes 2–6 correspond to MEIS1-FL overexpression, whereas lanes 7–11 correspond to MEIS1-HdL overexpression. Within these groups, the relevant control and shRNA conditions were compared to demonstrate that the MEIS1-FL-specific shRNA selectively suppresses MEIS1-FL without affecting MEIS1-HdL. We have corrected the lane annotation.

**Figure R1.** Validation of *sh-Meis1-FL* specificity. Immunoblotting of N2a cells co-transfected with MEIS1-FL or MEIS1-HdL and the indicated shRNAs confirmed that *sh-Meis1-FL (sh-Meis1-exon8)* specifically targets the FL isoform.

Regarding the GFP signal, this derives from the co-electroporated H3.1-GFP marker used to identify electroporated cells. To address the reviewer’s broader concern, we also expanded the relevant figure legends and the Materials and Methods to describe the experimental setup and quantification procedures more explicitly.

**Comment 20**

Reference numbers are listed after the period at the end of each sentence throughout.

**Response:** We thank the reviewer for noting this formatting issue. We have revised the reference formatting throughout the manuscript to comply with the journal style.

**Comment 21**

Lines 54-55 of the manuscript: Better say: "Each isoform has a different molecular structure and is produced at different developmental stages, in different cell types, or present in different subcellular locations." Isoforms are protein products, so strictly spoken they are not themselves expressed but generated by expression of a gene and its transcript variants.

**Response:** We thank the reviewer for this important terminological point. We have revised the sentence accordingly. It now reads: “Each isoform has a different molecular structure and is produced at different developmental stages, in different cell types, or present in different subcellular locations…”

This wording was revised in the Introduction and, where relevant, related terminology was adjusted elsewhere in the revised manuscript for consistency.

**Comment 22**

Transcript variants in Fig. 1F are shown in antisense / genomic orientation, which makes it difficult to match the exons shown as boxes with the protein domains they encode. Please indicate which exon codes for which domain or use the same color scheme for the exons in Fig.1F (Fig. S1E, G) and the protein domains in Fig. 1G (Fig. S2F, H).

**Response:** We appreciate this helpful suggestion. We have revised the relevant schematic figure (Figure 1G) to make the relationship between transcript structure and protein domains clearer, including clearer domain correspondence and harmonized color usage between the transcript and protein-domain panels.

**Comment 23**

Better use the terms Table S1, S2. "Supplementary File for list of…." is unnecessarily complicated.

**Response:** We agree and have simplified the terminology for supplementary materials throughout the manuscript. We now refer to these items consistently as S1 Data, S2 Data, S3 Data, and S4 Data, as appropriate.
