# Supplementary material for: Meis1 isoform diversity orchestrates neural progenitor differentiation by regulating ATOH1 degradation at distinct subcellular compartments
Source: PLoS Biol. 2026 Jul 13;24(7):e3003897. doi: 10.1371/journal.pbio.3003897 (PMC13379096; doi:10.1371/journal.pbio.3003897)
Supplement: Attachment — Submitted filename: Response-to-Reviewers_Revise_v3.docx [file pbio.3003897.s021.docx]

**Overview of the Revised Manuscript**

We thank the editors, the Academic Editor, and the reviewers for their careful and constructive evaluation of our revised manuscript. In this revision, we have addressed the remaining reviewer comments and the editorial requests. In particular, we revised Figure S10 to include the relevant non-NLS MEIS1-HdL control in the same figure as the NLS-MEIS1-HdL experiment, corrected the typographical error in Figure S10B, and revised the text to clarify that our data support a predominantly cytoplasmic MEIS1-HdL mechanism while not formally excluding additional nuclear contributions. We also revised the text concerning PBX family members to clarify that our experimental analysis tested PBX3 and that possible contributions from other PBX family proteins cannot be excluded.

In addition, we updated the Abstract to clearly state the model system and species studied, revised the Ethics Statement to include relevant Japanese national regulations and guidelines for animal care and use, added statements to the figure legends pointing readers to the underlying data file, updated the raw blot and gel image file in accordance with PLOS reporting requirements, and updated the Data Availability Statement to include the location of the custom code used in this study. Below, we provide a detailed point-by-point response to the editorial requests and reviewer comments.

**Point-by-Point Response to Editorial Requests**

**Editorial Request 1. ABSTRACT**

Please note that per journal policy, the model system/species studied should be clearly stated in the abstract of your manuscript.

**Response:** We have revised the Abstract to clearly state the model system and species studied. Specifically, we now indicate that the long-read cDNA sequencing was performed using mouse cerebellar granule cell progenitors (GCPs), and that the in vivo electroporation experiments were conducted in the postnatal mouse cerebellum.

**Changes made:** The relevant sentences in the Abstract have been revised as follows:

“Our long-read cDNA sequencing of mouse cerebellar granule cell progenitors (GCPs) identifies more than 700 genes with high isoform diversity.”

“In contrast, our in vivo electroporation experiments in the postnatal mouse cerebellum show that MEIS1-HdL inhibits GCP differentiation.”

**Editorial Request 2. ETHICS STATEMENT**

Please update your ethics statement, in your methods section to include the specific national or international regulations/guidelines to which your animal care and use protocol adhered. Please note that institutional or accreditation organization guidelines (such as AAALAC) do not meet this requirement.

**Response:** We have updated the Ethics Statement in the Materials and Methods section to specify the national regulations and guidelines followed for animal care and use. The revised statement now indicates that all animal experiments were conducted in accordance with relevant Japanese national regulations and guidelines, including the Act on Welfare and Management of Animals, the Standards Relating to the Care and Management of Laboratory Animals and Relief of Pain, and the Guidelines for Proper Conduct of Animal Experiments issued by the Science Council of Japan.

**Changes made:** The Animals section of the Materials and Methods has been revised as follows:

“All animal experiments in this study were approved by the Animal Care and Use Committee of the National Institute of Neuroscience, National Center of Neurology and Psychiatry (Tokyo, Japan; project 2019028R2), and were conducted in accordance with relevant Japanese national regulations and guidelines, including the Act on Welfare and Management of Animals, the Standards Relating to the Care and Management of Laboratory Animals and Relief of Pain, and the Guidelines for Proper Conduct of Animal Experiments issued by the Science Council of Japan. Mice were housed under specific pathogen-free conditions with food and water ad libitum. ICR pups were obtained from SLC (Japan). *Atoh1^AtGFP/+^* mice (The Jackson Laboratory: B6.129S-Atoh1tm4.1Hzo/J, Stock No. 013593) and *Ki67^KiRFP/+^* mice (The Jackson Laboratory: Mki67tm1.1Cle/J, Stock No. 029802) were obtained from The Jackson Laboratory.”

**Editorial Request 3. DATA**

Thank you for providing the underlying data for your study as a supplementary excel file (S5_data). Please add a sentence to every figure legend point readers to this file (ex, you can say ‘The data underlying this figure can be found in S5_data’)

**Response:** We have added a sentence to each figure legend indicating where the data underlying the figure can be found.

**Changes made:** A sentence has been added to each figure legend, for example:

“The data underlying this figure can be found in S5 Data.”

**Editorial Request 4. BLOT AND GEL REPORTING**

Thank you for providing a file with the western blot images related to your study (S1_raw images). It looked to me that some of the images are slightly cropped (although I understand that in some instances you cut the membrane before imaging, which is fine). Please update this file to provide the fully uncropped version of your blot images. Please also take a moment to read our reporting requirements, and to update the images to include relevant information such as loading order, identity of experimental samples, etc. The full details of our policy can be found here: https://journals.plos.org/plosbiology/s/figures#loc-blot-and-gel-reporting-requirements

**Response:** We have updated S1 Raw Images to provide fully uncropped blot and gel images wherever available. We have also updated the file to include relevant information, including the corresponding figure panel, antibody used, loading order, experimental sample identities, molecular weight markers where available, and markings for lanes or membrane regions not included in the final figures. In addition, when multiple regions from the same membrane were used in different figure panels, this is now indicated in S1 Raw Images.

During this update, we also identified and corrected a lane-labeling error in Fig. S8E. Specifically, the labels for GST-ATOH1 and GST-ATOH1-S328D were corrected in both the manuscript figure and S1 Raw Images. This correction affects only the lane annotation and does not alter the underlying data, quantification, or interpretation.

**Changes made:** S1 Raw Images has been updated to include fully uncropped source images, explanatory annotations, loading order, sample identities, and markings for lanes or membrane regions not included in the final figures. We also corrected the lane labels in Fig. S8E in both the manuscript and S1 Raw Images.

Editorial Request 5. **FIG S10**

I was wondering if there is a typo in Figure s10B. Should NLS-Meis1-fl be NLS-meis1-HdL?

**Response:** We thank the editor for pointing this out. This was a typographical error in Figure S10B. We have corrected “NLS-Meis1-FL” to “NLS-Meis1-HdL.”

**Changes made:** The label in Figure S10B has been corrected to “NLS-Meis1-HdL.”

**Editorial Request 6. CODE**

Per journal policy, if you have generated any custom code during the course of this investigation, please make it available without restrictions. Please ensure that the code is sufficiently well documented and reusable, and that your Data Statement in the Editorial Manager submission system accurately describes where your code can be found. More information on our Code Policy, what and how to share can be found here: https://journals.plos.org/plosbiology/s/code-availability

Please note that we cannot accept sole deposition of code in GitHub, as this could be changed after publication. However, you can archive this version of your publicly available GitHub code to Zenodo. Once you do this, it will generate a DOI number, which you will need to provide in the Data Accessibility Statement (you are welcome to also provide the GitHub access information). See the process for doing this here: <https://docs.github.com/en/repositories/archiving-a-github-repository/referencing-and-citing-content>

**Response:** We have made the custom code used for data processing, analysis, and figure generation publicly available. The code has been documented to indicate the required inputs, software dependencies, and the corresponding analyses/figures. In accordance with the journal policy, the corresponding version of the code has been archived in Zenodo, and the Zenodo DOI has been added to the Data Availability Statement and to the Data Statement in the Editorial Manager submission system. We also provide the corresponding GitHub repository information.

**Changes made:** The Data Availability Statement has been updated as follows:

“Custom code used for long-read cDNA-seq quality control, IsoQuant/SQANTI3 processing, isoform structure visualization, protein domain visualization, and gene-set overlap analysis is publicly available at Zenodo: <https://doi.org/10.5281/zenodo.20581171>. The corresponding GitHub repository is available at <https://github.com/Hoshino-lab/meis1-isoform-code>.”

**Point-by-Point Response to Reviewers**

**Response to Reviewer #1**

**Reviewer #1 comment:** While I appreciate the additional experiments to support MEIS1’s role in the cytoplasm, in particular, the NLS fusion experiment shown in Figure S10, the results are not definitive, as pointed out by the authors. In addition, the relevant control, i.e. the comparison with the non-NLS version of MEIS1HdL, should have been included in the same figure. While I understand that this is a high bar, I think more definitive experiments are required before the paper is suitable for Plos Biology. Because the current set of experiments are not definitive, the text has many qualifications, which ultimately dilute the message and its impact.

Further, regarding a potential role for Pbx factors, the authors only examined Pbx3, even though they say all three Pbx genes are expressed.

**Response:** We thank the reviewer for their careful evaluation of the revised manuscript and for acknowledging the additional experiments supporting the cytoplasmic role of MEIS1-HdL, including the NLS-MEIS1-HdL experiment. We acknowledge that the NLS fusion experiment does not formally exclude all possible nuclear contributions. However, as stated in the manuscript, the cumulative evidence supports a model in which the major ATOH1-stabilizing activity of MEIS1-HdL is mediated through a predominantly cytoplasmic CSN-CUL3 axis.

We also agree that the non-NLS MEIS1-HdL control should be shown in the same figure for direct comparison with NLS-MEIS1-HdL. We have therefore revised Fig. S10B and S10C to include the corresponding non-NLS MEIS1-HdL dose-response data. These data are the same as those shown in Fig. 6H and 6I and are reproduced in Fig. S10 for direct comparison with NLS-MEIS1-HdL. This revision allows readers to directly compare the ATOH1-stabilizing activity of cytoplasm-predominant MEIS1-HdL and nuclear-targeted NLS-MEIS1-HdL within the same figure.

We have also revised the text to focus the conclusion more clearly on what is directly supported by the data. Specifically, we now state that forced nuclear targeting of MEIS1-HdL attenuates its dose-dependent ATOH1-stabilizing activity, supporting the importance of cytoplasmic localization for the full activity of MEIS1-HdL, while minor nuclear contributions cannot be formally excluded.

Regarding PBX factors, we agree with the reviewer that our experimental analysis of PBX3 alone cannot exclude the possible involvement of other PBX family proteins. We have therefore revised the Results and Discussion to clarify that our data support a PBX3-independent mechanism, rather than a general PBX-independent mechanism. We also added text discussing the possible involvement of other PBX family members and stating that their contributions cannot be excluded without direct experimental analysis.

**Changes made:**

1. Fig. S10B and S10C have been revised to include the non-NLS MEIS1-HdL dose-response data as a control for direct comparison with NLS-MEIS1-HdL.
2. The Fig. S10 legend now states that the non-NLS MEIS1-HdL data are reproduced from Fig. 6H and 6I for direct comparison.
3. The title and legend of Fig. S10 have been revised to refer specifically to PBX3, rather than PBX proteins in general.
4. The Results and Discussion have been revised to describe the mechanism as predominantly cytoplasmic and PBX3-independent, while acknowledging that possible contributions from other PBX family proteins or additional nuclear mechanisms cannot be formally excluded.

**Response to Reviewer #3**

**Reviewer #3 comment:** I would like to thank the authors for their comprehensive and detailed responses to my questions and comments. All of my concerns have been addressed.

**Response:** We thank the reviewer for their positive assessment of our revised manuscript. We are pleased that all of the reviewer’s concerns have been addressed.

**Additional Literature**

We considered whether additional relevant literature published since the original submission should be cited in relation to the issues raised in this revision. No additional citations were necessary for the specific editorial and reviewer points addressed in this revision.
