# Supplementary material for: Meis1 isoform diversity orchestrates neural progenitor differentiation by regulating ATOH1 degradation at distinct subcellular compartments
Source: PLoS Biol. 2026 Jul 13;24(7):e3003897. doi: 10.1371/journal.pbio.3003897 (PMC13379096; doi:10.1371/journal.pbio.3003897)
Supplement: Peer review history

# Author comment: Author response to Decision Letter 0

**DOI:** 10.1371/journal.pbio.3003897.r001

4 Jan 2026

Attachment Submitted filename: Point-by-Point Response to Reviewers.docx

# Editor report: Decision Letter 0

**DOI:** 10.1371/journal.pbio.3003897.r002

**Authors:** Lucas Smith

16 Jan 2026

Dear Dr Hoshino,

Thank you for submitting your manuscript entitled "Meis1 isoform diversity orchestrates neural progenitor differentiation by regulating ATOH1 degradation at distinct subcellular compartments", which has been revised in response to a previous round of review, for consideration as a Research Article by PLOS Biology.

Your manuscript has now been evaluated by the PLOS Biology editorial staff and I am writing to let you know that we would like to send your submission back to the original reviewers, for their thoughts on the revision.

However, before we can send your manuscript to reviewers, we need you to complete your submission by providing the metadata that is required for full assessment. To this end, please login to Editorial Manager where you will find the paper in the 'Submissions Needing Revisions' folder on your homepage. Please click 'Revise Submission' from the Action Links and complete all additional questions in the submission questionnaire.

Once your full submission is complete, your paper will undergo a series of checks in preparation for peer review. After your manuscript has passed the checks it will be sent out for review. To provide the metadata for your submission, please Login to Editorial Manager (https://www.editorialmanager.com/pbiology) within two working days, i.e. by Jan 20 2026 11:59PM.

During the process of completing your manuscript submission, you will be invited to opt-in to posting your pre-review manuscript as a bioRxiv preprint. Visit http://journals.plos.org/plosbiology/s/preprints for full details. If you consent to posting your current manuscript as a preprint, please upload a single Preprint PDF.

Feel free to email us at plosbiology@plos.org if you have any queries relating to your submission.

Kind regards,

Luke

Lucas Smith, Ph.D.

Senior Editor

PLOS Biology

lsmith@plos.org

# Author comment: Author response to Decision Letter 1

**DOI:** 10.1371/journal.pbio.3003897.r003

20 Jan 2026

Attachment Submitted filename: Point-by-Point_Response_to_Reviewers_auresp_1.docx

# Peer review: Decision Letter 1

**DOI:** 10.1371/journal.pbio.3003897.r004

**Authors:** Lucas Smith, Lucas Smith

19 Mar 2026

Dear Dr Hoshino,

Thank you for your patience while we considered your revised manuscript "Meis1 isoform diversity orchestrates neural progenitor differentiation by regulating ATOH1 degradation at distinct subcellular compartments" for publication as a Research Article at PLOS Biology. I apologize for the protracted review process in this round of review. One of the reviewers needed a bit of extra time to assess the study, and we felt it was important to hear their feedback, given the substantial revisions that have taken place since the original submission. Your revised study has now been evaluated by the PLOS Biology editors, the Academic Editor and all three original reviewers.

The reviews are appended below. As you will see, the reviewers generally agree that the study has been greatly strengthened in this revision. However reviewers 1 and 3 have a number of important lingering concerns. After careful discussion, we think their concerns will need to be thoroughly addressed before we can consider your study for publication, and so we would like to invite you to revise the work to address the reviewers' reports.

Reviewer 1 has highlighted that more work is needed to strengthen the claims about the cytoplasmic function of Meis1, and we agree with this reviewer that more experiments are needed to rigorously test and bolster those claims. We also think that addressing Reviewer 3's comments will be essential to enhance the rigor of the study.

Given the extent of revision needed, we cannot make a decision about publication until we have seen the revised manuscript and your response to the reviewers' comments. Your revised manuscript may be sent for further evaluation by all or a subset of the reviewers.

In addition to these revisions, you will need to complete some formatting changes, which you will receive in a follow up email. A member of our team will be in touch with a set of requests shortly.

We expect to receive your revised manuscript within 3 months. Please email us (plosbiology@plos.org) if you have any questions or concerns, or would like to request an extension.

At this stage, your manuscript remains formally under active consideration at our journal; please notify us by email if you do not intend to submit a revision so that we may withdraw it.

**IMPORTANT - SUBMITTING YOUR REVISION**

Your revisions should address the specific points made by each reviewer. Please submit the following files along with your revised manuscript:

1. A 'Response to Reviewers' file - this should detail your responses to the editorial requests, present a point-by-point response to all of the reviewers' comments, and indicate the changes made to the manuscript.

*NOTE: In your point-by-point response to the reviewers, please provide the full context of each review. Do not selectively quote paragraphs or sentences to reply to. The entire set of reviewer comments should be present in full and each specific point should be responded to individually, point by point.

You should also cite any additional relevant literature that has been published since the original submission and mention any additional citations in your response.

2. In addition to a clean copy of the manuscript, please also upload a 'track-changes' version of your manuscript that specifies the edits made. This should be uploaded as a "Revised Article with Changes Highlighted" file type.

*Re-submission Checklist*

When you are ready to resubmit your revised manuscript, please refer to this re-submission checklist: https://plos.io/Biology_Checklist

To submit a revised version of your manuscript, please go to https://www.editorialmanager.com/pbiology/ and log in as an Author. Click the link labelled 'Submissions Needing Revision' where you will find your submission record.

Please make sure to read the following important policies and guidelines while preparing your revision:

*Published Peer Review*

Please note while forming your response, if your article is accepted, you may have the opportunity to make the peer review history publicly available. The record will include editor decision letters (with reviews) and your responses to reviewer comments. If eligible, we will contact you to opt in or out. Please see here for more details:

https://blogs.plos.org/plos/2019/05/plos-journals-now-open-for-published-peer-review/

*PLOS Data Policy*

Please note that as a condition of publication PLOS' data policy (http://journals.plos.org/plosbiology/s/data-availability) requires that you make available all data used to draw the conclusions arrived at in your manuscript. If you have not already done so, you must include any data used in your manuscript either in appropriate repositories, within the body of the manuscript, or as supporting information (N.B. this includes any numerical values that were used to generate graphs, histograms etc.). For an example see here: http://www.plosbiology.org/article/info%3Adoi%2F10.1371%2Fjournal.pbio.1001908#s5

*Blot and Gel Data Policy*

We require the original, uncropped and minimally adjusted images supporting all blot and gel results reported in an article's figures or Supporting Information files. We will require these files before a manuscript can be accepted so please prepare them now, if you have not already uploaded them. Please carefully read our guidelines for how to prepare and upload this data: https://journals.plos.org/plosbiology/s/figures#loc-blot-and-gel-reporting-requirements

*Protocols deposition*

To enhance the reproducibility of your results, we recommend that if applicable you deposit your laboratory protocols in protocols.io, where a protocol can be assigned its own identifier (DOI) such that it can be cited independently in the future. Additionally, PLOS ONE offers an option for publishing peer-reviewed Lab Protocol articles, which describe protocols hosted on protocols.io. Read more information on sharing protocols at https://plos.org/protocols?utm_medium=editorial-email&utm_source=authorletters&utm_campaign=protocols

Thank you again for your submission to our journal. We hope that our editorial process has been constructive thus far, and we welcome your feedback at any time. Please don't hesitate to contact us if you have any questions or comments.

Sincerely,

Luke

Lucas Smith, Ph.D.

Senior Editor

PLOS Biology

lsmith@plos.org

------------------------------------

REVIEWS:

Reviewer #1: The revised manuscript is greatly improved compared to the original submission, and I appreciate the detailed responses to the reviewers comments, the inclusion of many new experiments, and the more thoughtful interpretations of the data. My one remaining concern is how definitively the authors have demonstrated that MEIS1 functions outside of the nucleus, and not as a transcription factor. Subcellular localization studies are not definitive, especially in cases like this where the localization is not black and white -- there always seems to be some signal in the nucleus making it impossible to rule out a nuclear (transcriptional) function. Similarly, protein-protein interactions with other cytoplacmic proteins are also not definitive since, even if they exist, they do not prove that these interactions are required for MEIS1 function. What data can the authors point to that definitively rule out a nuclear function for the affects on ATOH1 stability? For example, can the authors disrupt a key protein interaction with a cytoplasmic protein and show that this abolishes MEIS1 function? Along the same lines, the authors do not consider interactions between MEIS1 and its well-known transcriptional partner PBX as a potential mechanism for either FL or HdL isoform function. Are PBX isoforms expressed in these cells? Can the authors demonstrate that PBX has no function here?

Once the authors address this one remaining concern and discuss alternative mechanisms that are not definitively ruled out by their experiments, this paper will make a valuable contribution to the field.

Reviewer #2: The authors have addressed all of this reviewer's comments, conducted additional experiments, and added key new data that consolidate the findings and strengthen their interpretations. These efforts have substantially improved the quality of the study and provide much stronger support for the authors' conclusions.

Reviewer #3: Manuscript PBIOLOGY-D-26-00016_R1, titled "Meis1 isoform diversity orchestrates neural progenitor differentiation by regulating ATOH1 degradation at distinct subcellular compartments" by Owa et al., builds on manuscript PBIOLOGY-D-22-02495R1, but represents a significant advancement and improvement. In particular, the manuscript benefits from the long-range sequencing performed by the authors, as well as the MEIS1-HdL-specific antibody. Overall, the statements in the manuscript are now much better supported by experimental evidence. However, some inconsistencies remain. These should, however, be easy to resolve.

1.Evidence for the existence of an HD-less Meis1 transcript variant: The authors performed nanopore sequencing to demonstrate that multiple, previously unknown transcript variants exist. This is very interesting but information on their frequency is missing. Please provide the number of times each of the transcript variants depicted in Fig. 1F were detected. This is important because very low-frequency splice junctions can be nonfunctional. For instance, it has been estimated that splicing errors occur at a rate of approx. 0.7% per intron. Novel isoforms detected at 0-1 TRM can therefore result from 'noisy splicing'. This concern also applies to the argument made in lines 131-133 ("To focus on robustly expressed isoforms, we applied a stringent filter, retaining only those transcripts with a TPM value greater than 1 in each of our three replicates"). Greater than 1TPM is still a relatively mild cut-off, given that on average 2% of transcripts from an average gene can be mis-spliced. It would strengthen the authors' argument if there was some evolutionary conservation, e.g. with the isoform described for hth by Noto et al., Genes & Dev. 2006 (see also argument 14 below).

2.Please provide the sequence of the transcript variants identified and the aa sequence of the isoforms translated from them shown in Fig. 1F, G.

3.Lines 193-194: "To test this, we generated a specific antibody against the unique 5-amino acid C-terminus of the predicted homeodomain-less isoform." Without sequence comparison between MEIS1-FL and MEIS1-HdL as well as information on how the MEIS1-HdL-specific antibody was generated, it remains obscure why the a-MEIS1 antibody (directed against the FL protein) and the a-MEIS1-HdL antibody (according to the authors distinct from MEIS1-FL due to a frameshift, line 564) recognize similar size bands in P7 GCPs (arrows in Fig. 2A). Where do the two proteins differ? Which antigens were used to generate the antibodies? Antibody production, including procedure and antigene used, is not mentioned in the Mat&Meth chapter.

4.Fig.2A does not proof beyond doubt that the MEIS1-HdL antibodies do not recognize any unrelated protein(s) in the extracts. Where does the second band come from that is recognized by MEIS1-FL and MEIS1-HdL antibodies? Validating antibodies with the overexpressed polypeptide that was used to immunize the rabbits is also a circular argument (Fig. S2A). Can the authors provide better proof of the specificity of their MEIS1-HdL abs? A simple approach would be to re-probe the blot shown in Fig. S2B with their custom-made antibody.

5.Lines 585/586: The authors state "Nanopore analysis definitively identified the exact full-length transcript corresponding to the MEIS1-HdL cDNA we cloned and used for overexpression." How does this relate to the statement that MEIS1-HdL contains a frameshift, which enabled the authors to generate isoform-specific antibodies? Is the frameshift contained in the overexpressed cDNA?

6.Line 263: "Overexpression of Meis1-FL significantly decreased the proportion of GCPs". How was the quantification done?

7.Lines 332/333 and lane 439: MEIS1(1-130) and MEIS1-HdL must be more clearly distinguished from one another. What was the rationale of including MEIS1(1-130)?

8.Line 380, Fig. 5G: The graph in Fig. 5H contains data for GST-ATOH1 w/o CHX, but the respective blot is not shown in Fig. 5G.

9.Line 403, Fig. S8D-F: The values for GST-ATOH1S238D (without co-transfection of MEIS1-FL or MEIS1-HdL) are not included in the graph in Fig.S8F. Conversely, the blots for GST-ATOH1 are not shown. The latter is important, because the biological relevance of the data in Fig. S8F depends on the comparison with the baseline values of GST-ATOH1 (black).

10.Line 435, Fig. 6B: Please comment on why COPS5 was tested in GST-PD assays when it was not identified my LC-MS/MS. Is this a sign that all COP9 signalosome components bind to MEIS1 or that they are generally 'sticky' towards the MEINOX homology domain? Can the authors provide a control TF, fused to GST, that does not bind to one of the COP9 complex subunits?

11.Reference to figure S9D is missing from the text

12.Lines 493/494: The authors have softened their claim that "MEIS1-HdL suppresses ATOH1 degradation more effectively than MEIS1-FL" to "MEIS1-HdL is capable of suppressing ATOH1 degradation even at low plasmid dosages". However, this does not fully alleviate the concern that the observed difference may be largely technical. This concern also applies to the statement "However, it was shown that MEIS1-HdL has a much stronger ATOH1-stabilization ability than MEIS1-FL." in the Discussion (lines 548/549).

13.Lines 503-503: "This difference in intracellular localization between MEIS1-FL and MEIS1-HdL probably accounts for the differential efficacy in suppressing CUL3-dependent ATOH1 degradation." The authors previously argue that "ATOH1 is polyubiquitinated by CUL3 in the cytoplasm, probably just after ATOH1 protein synthesis, while ATOH1 polyubiquitination in nuclei is elicited by other E3 ligases." This is a plausible argument for the idea that ATOH1 degradation in the cytoplasm and nucleus is driven by different processes, but it does not explain why degradation should be more efficient one subcellular compartment than the other. To answer that, one would need to know which E3 ligase regulates ATOH1 ubiquitination in the nucleus and how that differs from CUL3.

14.Line 551: If the authors want to include the publication by Hyman-Walsh into their argument, they need to consider that MEIS-autoinhibition is regulated by heterodimerization by PBX1. When the contribution of PBX1 to GC differentiation is unknown, this must be stated or the argument dropped. Other published work which the authors may want to consider: 1. Noro et al., Genes & Dev 2006, report a homeodomain-less isoform of hth that is functionally different from the full-length protein, as well as a HD-less isoform of Meis1 from mouse. It would be informative if the MEIS1-HdL isoform described here was compared against the isoform of Noro et al.. 2. MEIS2 is proteolytically cleaved by calpain proteases resulting in an HD-less cleavage product (Muller et al., JCS 2025).

15.Lines 571/572, temporal expression of MEIS1-FL during GC development: Please give citation.

16.Line 599, MEIS1 enhances the proliferation of hematopoietic stem cells: This sentence is oversimplified. Leukemia arises from differentiation arrest and self-renewal, not simply increased proliferation.

17.Lines 604-617: Glutamate receptor genes are a good example of how differential splicing of a gene produces functionally distinct isoforms, but this is somewhat of a stretch in the present context. Examples of differential splicing of transcription factors would be more appropriate. For instance, there is extensive literature on alternative splicing of Pax6, which the authors mention at the beginning of their study. There are also examples for functional differences between MEIS-family proteins, such as Noro et al. mentioned above, or MEIS2 in neuroblastoma (Gross et al., Cancer Res 2018).

18.The graphs look different. Some show individual data points, others show only error bars; sometimes statistical significance is indicated by a horizontal line, and sometimes only by asterisks above the bar. Please unify this.

Minor:

19.Fig. R1 was intended for the review process only, but there are still two points that need to be noted: 1. Panel A: Why does lane 2, but not lane 1 show a band at approximately 50 kDa, even though the same plasmids were transfected? 2. Panel B: Where does the GFP fluorescence come from? None of this is clear from the figure legend. This reflects an issue that was of considerable concern in the initial submission, has improved in the current submission, but has still not been fully resolved: The experimental approach is described so briefly and, in some cases, so superficially that the text becomes difficult to understand.

20.Reference numbers are listed after the period at the end of each sentence throughout.

21.Lines 54-55 of the manuscript: Better say: "Each isoform has a different molecular structure and is produced at different developmental stages, in different cell types, or present in different subcellular locations." Isoforms are protein products, so strictly spoken they are not themselves expressed but generated by expression of a gene and its transcript variants.

22.Transcript variants in Fig. 1F are shown in antisense / genomic orientation, which makes it difficult to match the exons shown as boxes with the protein domains they encode. Please indicate which exon codes for which domain or use the same color scheme for the exons in Fig.1F (Fig. S1E, G) and the protein domains in Fig. 1G (Fig. S2F, H).

23.Better use the terms Table S1, S2. "Supplementary File for list of…." is unnecessarily complicated.

# Author comment: Author response to Decision Letter 2

**DOI:** 10.1371/journal.pbio.3003897.r005

13 Apr 2026

Attachment Submitted filename: Response-to-Reviewes_Revise_v2.docx

# Peer review: Decision Letter 2

**DOI:** 10.1371/journal.pbio.3003897.r006

**Authors:** Lucas Smith, Lucas Smith, Lucas Smith

28 May 2026

Dear Dr Hoshino,

Thank you for your patience while we considered your revised manuscript "Meis1 isoform diversity orchestrates neural progenitor differentiation by regulating ATOH1 degradation at distinct subcellular compartments" for consideration as a Research Article at PLOS Biology. Your revised study has now been evaluated by the PLOS Biology editors, the Academic Editor and two of the original reviewers.

In light of the reviews, which you will find at the end of this email, we are pleased to offer you the opportunity to address the remaining points from the reviewers in a revision that we anticipate should not take you very long. We will then assess your revised manuscript and your response to the reviewers' comments with our Academic Editor aiming to avoid further rounds of peer-review, although we might need to consult with the reviewers, depending on the nature of the revisions.

You will see, below, that reviewer 3 is now fully satisfied by the revision. Reviewer 1 continues to have concerns about whether the new data are sufficiently definitive to support claims that the effects of the MEIS1-HdL isoform are mediated outside of the nucleus. While we appreciate reviewer 1's reservations, after careful discussion with the Academic Editor, we think that the cumulative weight of the evidence is adequate to support your model, and we think the paper is sufficiently interesting for PLOS Biology even with the qualifications and caveats that you discuss. So we would not require you to provide additional lines of evidence to bolster those claims.

With that said, we agree with reviewer 1 that it would be important to provide the data from the non-NLS version of MEIS1HDL as a control in figure S10. We think reviewer 1's comments about looking at other Pbx genes can be addressed with textual changes, and by discussing the other Pbx genes.

In addition to addressing these last points from the reviewers, I am also including a number of data and policy related requests in this email and we ask that you address them as well. These are detailed below.

**IMPORTANT - Please address the following editorial requests:

1) ABSTRACT: Please note that per journal policy, the model system/species studied should be clearly stated in the abstract of your manuscript.

2) ETHICS STATEMENT: Please update your ethics statement, in your methods section to include the specific national or international regulations/guidelines to which your animal care and use protocol adhered. Please note that institutional or accreditation organization guidelines (such as AAALAC) do not meet this requirement.

3) DATA: Thank you for providing the underlying data for your study as a supplementary excel file (S5_data). Please add a sentence to every figure legend point readers to this file (ex, you can say 'The data underlying this figure can be found in S5_data')

4) BLOT AND GEL REPORTING: Thank you for providing a file with the western blot images related to your study (S1_raw images). It looked to me that some of the images are slightly cropped (although I understand that in some instances you cut the membrane before imaging, which is fine). Please update this file to provide the fully uncropped version of your blot images. Please also take a moment to read our reporting requirements, and to update the images to include relevant information such as loading order, identity of experimental samples, etc. The full details of our policy can be found here: https://journals.plos.org/plosbiology/s/figures#loc-blot-and-gel-reporting-requirements

5) FIG S10 - I was wondering if there is a typo in Figure s10B. Should NLS-Meis1-fl be NLS-meis1-HdL?

6) CODE: Per journal policy, if you have generated any custom code during the course of this investigation, please make it available without restrictions. Please ensure that the code is sufficiently well documented and reusable, and that your Data Statement in the Editorial Manager submission system accurately describes where your code can be found. More information on our Code Policy, what and how to share can be found here: https://journals.plos.org/plosbiology/s/code-availability

Please note that we cannot accept sole deposition of code in GitHub, as this could be changed after publication. However, you can archive this version of your publicly available GitHub code to Zenodo. Once you do this, it will generate a DOI number, which you will need to provide in the Data Accessibility Statement (you are welcome to also provide the GitHub access information). See the process for doing this here: https://docs.github.com/en/repositories/archiving-a-github-repository/referencing-and-citing-content

In addition to these revisions, you may need to complete some formatting changes, which you will receive in a follow up email. A member of our team will be in touch with a set of requests shortly. If you do not receive a separate email within a few days, please assume that checks have been completed, and no additional changes are required.

We expect to receive your revised manuscript within 1 month. Please email us (plosbiology@plos.org) if you have any questions or concerns, or would like to request an extension.

At this stage, your manuscript remains formally under active consideration at our journal; please notify us by email if you do not intend to submit a revision so that we withdraw the manuscript.

**IMPORTANT - SUBMITTING YOUR REVISION**

Your revisions should address the specific points made by each reviewer. Please submit the following files along with your revised manuscript:

1. A 'Response to Reviewers' file - this should detail your responses to the editorial requests, present a point-by-point response to all of the reviewers' comments, and indicate the changes made to the manuscript.

*NOTE: In your point-by-point response to the reviewers, please provide the full context of each review. Do not selectively quote paragraphs or sentences to reply to. The entire set of reviewer comments should be present in full and each specific point should be responded to individually.

You should also cite any additional relevant literature that has been published since the original submission and mention any additional citations in your response.

2. In addition to a clean copy of the manuscript, please also upload a 'track-changes' version of your manuscript that specifies the edits made. This should be uploaded as a "Revised Article with Changes Highlighted " file type.

*Resubmission Checklist*

When you are ready to resubmit your revised manuscript, please refer to this resubmission checklist: https://plos.io/Biology_Checklist

To submit a revised version of your manuscript, please go to https://www.editorialmanager.com/pbiology/ and log in as an Author. Click the link labelled 'Submissions Needing Revision' where you will find your submission record.

Please make sure to read the following important policies and guidelines while preparing your revision:

*Published Peer Review*

Please note while forming your response, if your article is accepted, you may have the opportunity to make the peer review history publicly available. The record will include editor decision letters (with reviews) and your responses to reviewer comments. If eligible, we will contact you to opt in or out. Please see here for more details:

https://blogs.plos.org/plos/2019/05/plos-journals-now-open-for-published-peer-review/

*PLOS Data Policy*

Please note that as a condition of publication PLOS' data policy (http://journals.plos.org/plosbiology/s/data-availability) requires that you make available all data used to draw the conclusions arrived at in your manuscript. If you have not already done so, you must include any data used in your manuscript either in appropriate repositories, within the body of the manuscript, or as supporting information (N.B. this includes any numerical values that were used to generate graphs, histograms etc.). For an example see here: http://www.plosbiology.org/article/info%3Adoi%2F10.1371%2Fjournal.pbio.1001908#s5

*Blot and Gel Data Policy*

We require the original, uncropped and minimally adjusted images supporting all blot and gel results reported in an article's figures or Supporting Information files. We will require these files before a manuscript can be accepted so please prepare them now, if you have not already uploaded them. Please carefully read our guidelines for how to prepare and upload this data: https://journals.plos.org/plosbiology/s/figures#loc-blot-and-gel-reporting-requirements

*Protocols deposition*

To enhance the reproducibility of your results, we recommend that if applicable you deposit your laboratory protocols in protocols.io, where a protocol can be assigned its own identifier (DOI) such that it can be cited independently in the future. Additionally, PLOS ONE offers an option for publishing peer-reviewed Lab Protocol articles, which describe protocols hosted on protocols.io. Read more information on sharing protocols at https://plos.org/protocols?utm_medium=editorial-email&utm_source=authorletters&utm_campaign=protocols

Thank you again for your submission to our journal. We hope that our editorial process has been constructive thus far, and we welcome your feedback at any time. Please don't hesitate to contact us if you have any questions or comments.

Sincerely,

Luke

Lucas Smith, Ph.D.

Senior Editor

PLOS Biology

lsmith@plos.org

----------------------------------------------------------------

REVIEWS:

Reviewer #1: While I appreciate the additional experiments to support MEIS1's role in the cytoplasm, in particular, the NLS fusion experiment shown in Figure S10, the results are not definitive, as pointed out by the authors. In addition, the relevant control, i.e. the comparison with the non-NLS version of MEIS1HdL, should have been included in the same figure. While I understand that this is a high bar, I think more definitive experiments are required before the paper is suitable for Plos Biology. Because the current set of experiments are not definitive, the text has many qualifications, which ultimately dilute the message and its impact.

Further, regarding a potential role for Pbx factors, the authors only examined Pbx3, even though they say all three Pbx genes are expressed.

Reviewer #3: I would like to thank the authors for their comprehensive and detailed responses to my questions and comments. All of my concerns have been addressed.

# Author comment: Author response to Decision Letter 3

**DOI:** 10.1371/journal.pbio.3003897.r007

7 Jun 2026

Attachment Submitted filename: Response-to-Reviewers_Revise_v3.docx

# Editor report: Decision Letter 3

**DOI:** 10.1371/journal.pbio.3003897.r008

**Authors:** Lucas Smith, Lucas Smith, Lucas Smith, Lucas Smith

24 Jun 2026

Dear Dr Hoshino,

Thank you for the submission of your revised Research Article "Meis1 isoform diversity orchestrates neural progenitor differentiation by regulating ATOH1 degradation at distinct subcellular compartments" for publication in PLOS Biology and thank you for addressing the last reviewer and editorial requests in this revision. Please also accept my apologies for the delay in sending this decision - I have been working through a bit of a backlog after taking some sick time earlier this month. On behalf of my colleagues and the Academic Editor,Claude Desplan, I am pleased to say that we are satisfied with the revision and that we can in principle accept your manuscript for publication, provided you address any remaining formatting and reporting issues. These will be detailed in an email you should receive within 2-3 business days from our colleagues in the journal operations team; no action is required from you until then. Please note that we will not be able to formally accept your manuscript and schedule it for publication until you have completed any requested changes.

Please take a minute to log into Editorial Manager at http://www.editorialmanager.com/pbiology/, click the "Update My Information" link at the top of the page, and update your user information to ensure an efficient production process.

PRESS

We frequently collaborate with press offices. If your institution or institutions have a press office, please notify them about your upcoming paper at this point, to enable them to help maximise its impact. If the press office is planning to promote your findings, we would be grateful if they could coordinate with biologypress@plos.org. If you have previously opted in to the early version process, we ask that you notify us immediately of any press plans so that we may opt out on your behalf.

We also ask that you take this opportunity to read our Embargo Policy regarding the discussion, promotion and media coverage of work that is yet to be published by PLOS. As your manuscript is not yet published, it is bound by the conditions of our Embargo Policy. Please be aware that this policy is in place both to ensure that any press coverage of your article is fully substantiated and to provide a direct link between such coverage and the published work. For full details of our Embargo Policy, please visit http://www.plos.org/about/media-inquiries/embargo-policy/.

Thank you again for choosing PLOS Biology for publication and supporting Open Access publishing. We look forward to publishing your study.

Sincerely,

Luke

Lucas Smith, Ph.D.

Senior Editor

PLOS Biology

lsmith@plos.org
